# Catalytic Synthesis of Oligosiloxanes Mediated by an Air Stable Catalyst, (C_6_F_5_)_3_B(OH_2_)

**DOI:** 10.3389/fchem.2020.00477

**Published:** 2020-06-23

**Authors:** Kristel M. Rabanzo-Castillo, Vipin B. Kumar, Tilo Söhnel, Erin M. Leitao

**Affiliations:** ^1^School of Chemical Sciences, University of Auckland, Auckland, New Zealand; ^2^The MacDiarmid Institute for Advanced Materials and Nanotechnology, Auckland, New Zealand

**Keywords:** siloxane, lewis acid catalysis, dehydrocoupling, catalyst recycling, silane, competing mechanisms

## Abstract

The utility of (C_6_F_5_)_3_B(OH_2_) as catalyst for the simple and environmentally benign synthesis of oligosiloxanes directly from hydrosilanes, is reported. This protocol offers several advantages compared to other methods of synthesizing siloxanes, such as mild reaction conditions, low catalyst loading, and a short reaction time with high yields and purity. The considerable H_2_O-tolerance of (C_6_F_5_)_3_B(OH_2_) promoted a catalytic route to disiloxanes which showed >99% conversion of three tertiary silanes, Et_3_SiH, PhMe_2_SiH, and Ph_3_SiH. Preliminary data on the synthesis of unsymmetrical disiloxanes (Si-O-Si') suggests that by modifying the reaction conditions and/or using a 1:1 combination of silane to silanol the cross-product can be favored. Intramolecular reactions of disilyl compounds with catalytic (C_6_F_5_)_3_B(OH_2_) led to the formation of novel bridged siloxanes, containing a Si-O-Si linkage within a cyclic structure, as the major product. Moreover, the reaction conditions enabled recovery and recycling of the catalyst. The catalyst was re-used 5 times and demonstrated excellent conversion for each substrate at 1.0 mol% catalyst loading. This seemingly simple reaction has a rather complicated mechanism. With the hydrosilane (R_3_SiH) as the sole starting material, the fate of the reaction largely depends on the creation of silanol (R_3_SiOH) from R_3_SiH as these two undergo dehydrocoupling to yield a disiloxane product. Generation of the silanol is based on a modified Piers-Rubinsztajn reaction. Once the silanol has been produced, the mechanism involves a series of competitive reactions with multiple catalytically relevant species involving water, silane, and silanol interacting with the Lewis acid and the favored reaction cycle depends on the concentration of various species in solution.

## Introduction

**Scheme 1:**
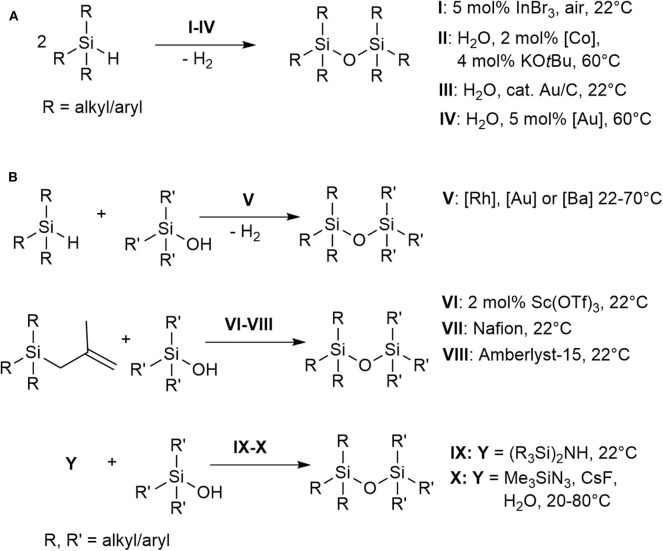
Selected examples of the catalytic synthesis of disiloxanes.

Organo(poly)siloxanes (silicones), bearing the repeating Si-O bond motif, are considered one of the most important classes of functional materials that have influenced many technological industries (Sawama et al., 2016; Wang et al., 2017). Industrially, polysiloxanes are generated by acid- or base-catalyzed ring opening polymerization of cyclic siloxanes, or by hydrolysis of chlorosilanes (Grubb, 1954; Brinker and Scherer, 1990). However, these methods have limited control over the oligomeric or polymeric siloxane sequence being formed. The substitution process is catalyzed either by an acid or a base under equilibrium in which the polysiloxanes can also be degraded (Brinker and Scherer, 1990). Although a step-wise synthesis of oligosiloxane has been reported, it is still based on conventional condensation of silanols with chlorosilanes (Uchida et al., 1990; Matsumoto et al., 2018, 2019). Other methods available in literature for the preparation of oligosiloxanes involve catalytic cross-coupling reactions of oxygen nucleophiles (e.g., alkoxysilanes, silanols, phenols, and ethers) with hydrosilanes (Brook, 2018; Zhang et al., 2020). The synthesis of symmetrical disiloxanes directly from hydrosilanes have been reported using InBr_3_, a reaction which involves Lewis acid-catalyzed air oxidation of hydrosilanes (Scheme 1, **A**, conditions **I**) (Sridhar et al., 2009). Reduction of CO_2_ with hydrosilanes and a zirconium complex/B(C_6_F_5_)_3_ as the catalyst have also been reported to produce methane and oligosiloxanes as products (Matsuo and Kawaguchi, 2006). Other direct syntheses reported the use of H_2_O as the solvent and oxidant, however, generally these involved expensive transition metal catalysts and are conducted at elevated temperature (Scheme 1, **A**, conditions **II–IV**) (Lee et al., 2000, 2004; Ison et al., 2005; Mitsudome et al., 2008, 2009; Chauhan et al., 2009; Asao et al., 2010; John et al., 2011; Tan et al., 2011; Jeon et al., 2012; Shimizu et al., 2012a,b; Liu et al., 2014; Sawama et al., 2016; Tsuchido et al., 2020). The development of synthetic methods catalyzed by abundant and cheap base-metal complexes then emerged to form symmetrical and unsymmetrical disiloxanes and other oligosiloxanes (Pattanaik and Gunanathan, 2019) (Scheme 1, **B**, conditions **V)**. Catalytic routes toward unsymmetrical siloxanes (Si-O-Si') have also been demonstrated (Scheme 1, **B**, conditions **VI–VIII**) using Sc(OTf)_3_ (Hreczycho et al., 2013; Hreczycho, 2015), Nafion (Kaźmierczak and Hreczycho, 2018), or Amberlyst-15 (Kuciński and Hreczycho, 2019a) with silanols and alkylsilanes at room temperature. Transition metal catalyzed routes to unsymmetrical disiloxanes have been achieved through Pd-catalyzed arylation of hydroxysiloxanes (Kurihara et al., 2013), nonhydrolytic Pd/C-catalyzed cross-coupling reactions (Igarashi et al., 2014), Ba-mediated dehydrocoupling of hydrosilanes and silanols (Le Coz et al., 2019) and silylation of silanols catalyzed by a ruthenium complex (Marciniec et al., 2008). Other routes to form Si-O-Si' bonds include the use of fluoride and azidosilanes (Abele et al., 2003) as well as a catalyst-free, chlorine-free option using disilazanes (Kuciński and Hreczycho, 2019b) (Scheme 1, **B**, conditions **IX–X**).

Main group (and metal-free) catalysts such as B(C_6_F_5_)_3_ have gained great interest as a catalyst activator and as strong Lewis acid for many purposes (Piers, 2004; Lawson and Melen, 2017; Brook, 2018). Several studies have been published for the preparation of siloxanes and hyperbranched siloxanes involving catalytic cross-coupling reactions of oxygen nucleophiles (e.g., alkoxysilanes, silanols, phenols, and ethers) with hydrosilanes (Kawakami et al., 2004; Chojnowski et al., 2005, 2006, 2008; Zhou and Kawakami, 2005; Shinke et al., 2007; Thompson and Davies, 2007; Cella and Rubinsztajn, 2008; Kurjata et al., 2009; Feghali and Cantat, 2014; Madsen et al., 2014; Zhang et al., 2014, 2020; Feghali et al., 2015; Laengert et al., 2017; Szawiola et al., 2017; Wu et al., 2017; Brook, 2018; Ai et al., 2019). Recently, Matsumoto and co-workers reported a sequence-controlled synthesis of oligosiloxanes via dehydrocarbonative coupling of alkoxysilanes and hydrosilanes, named the Piers-Rubinsztajn reaction, and also via hydrosilylation of carbonyl compounds using B(C_6_F_5_)_3_ (Matsumoto et al., 2018). Several mechanistic studies have been demonstrated for the formation of siloxanes depending on the type of substrates and catalysts used (Brook, 2018; Pattanaik and Gunanathan, 2019). The standard Piers-Rubinsztajn reaction involves the condensation of a hydrosilane (R_3_SiH) and alkoxysilane (R_3_SiOR) to form a siloxane, with subsequent removal of an alkane, RH (R = alkyl/aryl). Knowledge of this mechanism has allowed for the design of controlled synthetic routes to polysiloxanes using B(C_6_F_5_)_3_ as the catalyst (Chojnowski et al., 2005; Rubinsztajn and Cella, 2005; Cella and Rubinsztajn, 2008; Yi et al., 2018; Schneider et al., 2019). The strong affinity of B(C_6_F_5_)_3_ with H_2_O can lead to the formation of Brønsted acids which seems to not be a problem in terms of siloxane or polymer synthesis (Neumann et al., 2004; Chojnowski et al., 2005; Longuet et al., 2007). However, B(C_6_F_5_)_3_ can form several complexes with H_2_O which serve to remove the active catalyst from the reaction (Brook, 2018). In the presence of excess H_2_O therefore, the Piers-Rubinsztajn reaction is not observed or leads to lower rate of reaction.

In this article, we report the synthesis of oligomeric siloxanes starting from hydrosilanes and tethered hydrosilanes using controlled amounts of H_2_O. Specifically, we explored the possibility of replacing the B(C_6_F_5_)_3_ catalyst with (C_6_F_5_)_3_B(OH_2_) aimed at understanding the effect of the moisture stable (C_6_F_5_)_3_B(OH_2_) catalyst on the selectivity, the recyclability and the mechanism of siloxane formation.

## Materials and Methods

All reactions and manipulations were performed under a nitrogen atmosphere in an MBraun Unilab 1200/780 glovebox or using conventional Schlenk techniques, unless otherwise specified. Dry solvents were obtained using a solvent purification system. Reagents were purchased from Sigma-Aldrich, AK Scientific, Arcos and TCI and used as received. (C_6_F_5_)_3_B(OH_2_) (Beringhelli et al., 2001) was made by adding a stoichiometric amount of H_2_O to B(C_6_F_5_)_3_ followed by purification by sublimation. ^1^H, ^13^C, and ^29^Si NMR spectra were recorded on a Bruker DPX-400 (400MHz) spectrometer. Chemical shifts for protons are reported in parts per million (ppm) downfield from tetramethylsilane and are referenced to residual protium in the NMR solvent (e.g., CHCl_3_ = 7.26 ppm). Chemical shifts for carbon are reported in ppm downfield from CDCl_3_ (77.3 ppm). Chemical shifts for silicon are reported in ppm downfield to the silicon resonance of tetramethylsilane (TMS δ 0.0). The silicon NMR resonances were determined with a DEPT pulse sequence. Data are represented as follows: chemical shift, multiplicity (app = apparent, br = broad, s = singlet, d = doublet, t = triplet, q = quartet, m = multiplet), coupling constants in Hertz (Hz), and integration. High resolution mass spectrometry measurements were made on a Bruker microTOF-QII mass spectrometer, equipped with a KD Scientific syringe pump, in positive ion ESI mode. Hard ionization mass spectrometry analysis was done on Agilent 7890A GC + 5975C EI-MS with Agilent auto-sampler. X-ray diffraction analysis of single crystals of **7a** and **8a** were performed on a Rigaku Oxford Diffraction XtaLAB-Synergy-S single crystal diffractometer with a PILATUS 200 K hybrid pixel array detector using Cu Kα radiation (Supplementary Table 1). The data was processed with the SHELX2016 (Sheldrick, 2015) and Olex2 (Dolomanov et al., 2009) software packages. All non-hydrogen atoms were refined anisotropically. Hydrogen atoms were inserted at calculated positions and refined with a riding model or without restrictions. **8a** was refined on a HKL5 dataset extracted from PLATON (Spek, 2003). Mercury 4.2.0 (Macrae et al., 2006) was used to visualize the molecular structures.

### Intermolecular Reactions

#### Synthesis of Tertiary Disiloxanes, 3a-3c

##### Et_3_SiOEt_3_ (3a) and PhMe_2_SiOSiMe_2_Ph (3b)

To a mixture of hydrosilane (Et_3_SiH, **1a**, 5.0 mmol, 0.80 mL or PhMe_2_SiH, **1b**, 5.0 mmol, 0.77 mL) and 0.1–5.0 mol% (C_6_F_5_)_3_B(OH_2_), was added H_2_O (2.5 mmol, 0.050 mL) while stirring at room temperature (22°C). The reaction was monitored using ^1^H and ^29^Si{^1^H} NMR spectroscopy at specific time interval using an insert containing deuterated solvent. Yield: **3a**: 70.6%, **3b**: 94.0%.

##### 3a (Sridhar et al., 2009; Jorapur and Shimada, 2012)

^1^H NMR (400 MHz CDCl_3_): δ 0.95–0.91 (t, CH_3_, 6H), δ 0.55–0.49 (q, CH_2_, 4H). ^13^C{^1^H} NMR (100.6 MHz, CDCl_3_): δ 6.7 (s, CH_2_), δ 6.4 (s, CH_3_). ^29^Si{^1^H} NMR (79.5 MHz, CDCl_3_): δ 8.9 ppm (s). GC-MS: cal'd: 246.1835 *m/z;* observed 246.1900 *m/z*.

##### 3b (Jorapur and Shimada, 2012; Sawama et al., 2016)

^1^H NMR (400 MHz CDCl_3_): δ 7.57–7.36 (m, Ph, 10H), δ 0.35 (s, CH_3_, 12H). ^13^C{^1^H} NMR (100.6 MHz, CDCl_3_): δ 139.8, δ 133.0, δ 129.4, δ 127.7, δ 0.85 (s, CH_3_). ^29^Si{^1^H} NMR (79.5 MHz, CDCl_3_): δ 0.01 ppm (s). HRMS-ESI: [C_16_H_22_OSi_2_Na]^+^ = cal'd: 309.1101 *m/z;* observed 309.1091 *m/z*.

##### Ph_3_SiOSiPh_3_, 3c

To a mixture of Ph_3_SiH (**1c**; 2.0 mmol, 0.26 g) and 0.1–5.0 mol% (C_6_F_5_)_3_B(OH_2_) dissolved in 0.50 mL toluene-d_8_, was added H_2_O (1.0 mmol, 0.02 mL) while stirring at room temperature (22°C). The reaction was monitored using ^1^H and ^29^Si{^1^H} NMR spectroscopy at specific time interval. Yield: 98.0%.

##### 3c (Jorapur and Shimada, 2012)

^1^H NMR (400 MHz CDCl_3_): δ 7.49–7.24 (m, Ph, 30H). ^13^C{^1^H} NMR (100.6 MHz, CDCl_3_): δ 135.5, δ 135.2, δ 129.8, δ 127.7. ^29^Si{^1^H} NMR (79.5 MHz, CDCl_3_): δ −18.6 ppm (s). HRMS-ESI: [C_36_H_30_OSi_2_Na]^+^ cal'd: 557.1733 *m/z;* observed 557.1701 *m/z*.

#### Synthesis of Secondary Oligosiloxanes, (Cyclo) 3d-3f, (Cyclo) 4e-4f, 5e

To a mixture of 4.0 mmol hydrosilane (0.52 mL Et_2_SiH_2_, **1d**; 0.55 mL PhMeSiH_2_, **1e**; 0.76 mL Ph_2_SiH_2_, **1f**) and 0.1–5.0 mol% (C_6_F_5_)_3_B(OH_2_), was added 2.0 mmol, 0.04 mL H_2_O while stirring at room temperature (22°C). The reaction was monitored using ESI-MS at specific time interval. All products were filtered through Florisil to remove the catalyst using *n*-pentane or hexanes (10 ml) as eluent. ESI-MS: **3d (trimer)**: [C_14_H_36_O_4_Si_3_Na]^+^ cal'd: 375.1819 *m/z*; observed 375.1812 *m/z*, **cyclo-3d (cyclic trimer)**: [C_13_H_33_O_3_Si_3_Na]^+^ cal'd: 321.1737 *m/z*; observed 321.1731 *m/z*, **3e (trimer)**: [C_23_H_30_O_4_Si_3_Na]^+^ cal'd: 477.1350 *m/z*; observed 477.1337 *m/z*, **4e (tetramer)**: [C_28_H_34_O_5_Si_4_Na]^+^ cal'd: 585.1381 *m/z*; observed 585.1357 *m/z*, **5e (pentamer)**: [C_35_H_42_O_6_Si_5_Na]^+^ cal'd: 721.1725 *m/z*; observed 721.1657 *m/z*, **cyclo-3f (cyclic trimer)**: [C_36_H_30_O_3_Si_3_Na]^+^ cal'd: 617.1400 *m/z*; observed 617.1356 *m/z*, **cyclo-4f (cyclic tetramer)**: [C_48_H_40_O_4_Si_4_Na]^+^ cal'd: 815.1901 *m/z*; observed 815.1837 *m/z*.

### Intramolecular Reactions

#### Synthesis of Disilyl Precursors, 7a-7c

To an oven dried two neck round bottom flask purged with nitrogen was added magnesium turnings (12.0 mmol, 0.29 g), dry THF (5 mL) and diphenylchlorosilane (**7a**; 12.0 mmol, 2.3 mL) or dimethylchlorosilane (**7b, 7c**; 12.0 mmol, 1.3 mL). To this suspension was added 2-bromobenzylbromide (**6a;** 3.0 mmol, 0.75 g) or α,α'-dibromo-o-xylene (**6b**; 3.0 mmol, 0.79 g) dissolved in dry THF (10 mL) dropwise over a period of 15 min. The mixture was refluxed for 1 h and then stirred at 22°C overnight. The reaction mixture was quenched with saturated solution of NaHCO_3_ (5 mL) and the aqueous layer was extracted with diethyl ether (3 ×10 mL). All the organic layers were combined and dried over Na_2_SO_4_. The crude product was obtained upon removal of solvents under vacuum.

##### (2-(diphenylsilyl)benzyl)diphenylsilane (7a)

The crude product was dissolved in *n*-pentane (20 mL) and the mixture was stored at −20°C overnight. Colorless crystals were filtered off and were washed with cold pentane (2 ×5 mL) to obtain the pure product in 72.3% yield.

^1^H NMR (400 MHz CDCl_3_): δ 7.45–6.97 (m, Ph, 24H), δ 5.53 (s, SiH, 1H), δ 4.8 (t, SiH, 1H), 2.8 (d, CH_2_, 2H). ^13^C{^1^H} NMR (100.6 MHz, CDCl_3_): δ 145.67, δ 137.16, δ 135.95, δ 135.38, δ 134.32, δ 133.58, δ 133.39, δ 131.36, δ 129.98, δ 129.66, δ 128.01, δ 127.89, δ 124.28, δ 23.06. ^29^Si{^1^H} NMR (79.5 MHz, CDCl_3_): δ −13.70, δ −22.48 ppm. HRMS-ESI: [C_31_H_28_Si_2_Na]^+^ cal'd: 479.1626 *m/z*; observed: 479.1615 *m/z*.

##### (2-(dimethylsilyl)benzyl)diphenylsilane (7b)

The crude product was purified over silica gel column chromatography using hexanes as the eluent and was obtained as a colorless oil in 80.8% yield.

^1^H NMR (400 MHz CDCl_3_): δ 7.45–7.05 (m, Ph, 4H), δ 4.5 (sep, SiH, 1H), δ 3.9 (sep, SiH, 1H), 2.3 (d, CH_2_, 2H), δ 0.3 (d, CH_3_, 6H), δ 0.1 (d, CH_3_, 6H). ^13^C{^1^H} NMR (100.6 MHz, CDCl_3_): δ 146.04, δ 134.77, δ 129.34, δ 128.32, δ 123.85, δ 24.39, δ −3.06, δ −4.26. ^29^Si{^1^H} NMR (79.5 MHz, CDCl_3_): δ −11.04, δ −21.63 ppm. HRMS-ESI: [C_11_H_20_Si_2_H]^+^ cal'd: 209.1181 *m/z*; observed: 209.1171 *m/z*.

##### 1,2-bis((dimethylsilyl)methyl)benzene (7c)

The crude product was purified over silica gel column chromatography using hexanes as the eluent and was obtained as colorless oil in 52.5% yield.

^1^H NMR (400 MHz CDCl_3_): δ 6.99 (s, Ph, 4H), δ 4.0 (sep, SiH, 1H), δ 2.1 (d, CH_2_, 2H), δ 0.1 (d, CH_3_, 12H). ^13^C{^1^H} NMR (100.6 MHz, CDCl_3_): δ 136.71, δ 129.19, δ 124.47, δ 21.61, δ −4.21. ^29^Si{^1^H} NMR (79.5 MHz, CDCl_3_): δ−13.28 ppm. GC-MS: cal'd: 222.1260 *m/z;* observed: 222.1000 *m/z*.

#### Synthesis of Bridged Siloxanes, 8a-8c

##### 1,1,3,3-tetraphenyl-3,4-dihydro-1H-2,1,3-benzoxadisiline (8a)

To a round bottom flask was added **7a** (1.0 mmol, 0.45 g) and 5.0 mol% (C_6_F_5_)_3_B(OH_2_). The solids were dissolved in toluene (10 mL) and the reaction mixture was stirred at 90°C for 24 h. Toluene was removed under vacuum and the crude product was dissolved in hexanes (10 mL) which was then filtered through a Florisil pad with hexanes as eluent. Upon removal of volatiles the product was obtained as white solids. After recrystallization in ethyl acetate a pure crystalline product with 45.2% yield was obtained.

^1^H NMR (400 MHz CDCl_3_): δ 7.57–7.06 (m, Ph, 24H), δ 2.64 (s, CH_2_, 2H). ^13^C{^1^H} NMR (100.6 MHz, CDCl_3_): δ 145.04, δ 135.42, δ 135.14, δ 134.97, δ 134.31, δ 130.56, δ 130.48, δ 130.13, δ 130.02, δ 127.86, δ 124.63, δ 23.66. ^29^Si{^1^H} NMR (79.5 MHz, CDCl_3_): δ −9.91, −14.11 ppm. HRMS-ESI: [C_31_H_26_OSi_2_Na]^+^ cal'd: 493.1419 *m/z*; observed: 493.1421 *m/z*.

##### 1,1,3,3-tetramethyl-3,4-dihydro-1H-2,1,3-benzoxadisiline (8b)

To a vial was added 1.0 mol% (C_6_F_5_)_3_B(OH_2_), 0.5 mL toluene and **7b** (1.0 mmol, 0.21 g). The reaction mixture was stirred for 3 h at 22°C. Toluene was then removed under reduced pressure and hexanes (3 mL) was added to the mixture. Upon Florisil filtration with hexanes as eluent and removal of volatiles the product was obtained as clear oil with 61.8% yield.

^1^H NMR (400 MHz CDCl_3_): δ 7.40–7.12 (m, Ph, 4H), δ 2.19 (s, CH_2_, 2H), δ 0.38 (s, CH_3_, 6H), δ 0.16 (s, CH_3_, 6H). ^29^Si{^1^H} NMR (79.5 MHz, CDCl_3_): δ 10.85, δ 3.66 ppm. HRMS-ESI: [C_11_H_18_OSi_2_H]^+^ cal'd: 223.0974 *m/z*; observed: 223.0925 *m/z*.

##### 2,2,4,4-tetramethyl-1,2,4,5-tetrahydrobenzoxadisilepine (8c)

To a vial was added 1.0 mol% (C_6_F_5_)_3_B(OH_2_), 0.5 mL toluene and **7c** (1.0 mmol, 0.22 g). The reaction mixture was stirred for 3 h at 22°C. Toluene was then removed under reduced pressure and hexanes (3 mL) was added to the mixture. Upon Florisil filtration with hexanes as eluent and removal of volatiles the product was obtained as clear oil with 30.6% yield.

^1^H NMR (400 MHz CDCl_3_): δ 7.03–6.95 (m, Ph, 4H), δ 2.16 (s, CH_2_, 2H), δ 0.07 (s, CH_3_, 12H). ^13^C{^1^H} NMR (100.6 MHz, CDCl_3_): δ 137.38, δ 129.35, δ 125.04, δ 27.48, δ 0.0. ^29^Si{^1^H} NMR (79.5 MHz, CDCl_3_): δ 7.23 ppm. HRMS-ESI: [C_12_H_20_OSi_2_H]^+^ cal'd: 237.1130 *m/z*; observed: 237.1123 *m/z*.

### Cross-Condensation Reactions

#### 1:1 reaction of 1a and 1c

To a mixture of 1 equiv. **1a**, 1 equiv. **1c** and 5.0 mol% (C_6_F_5_)_3_B(OH_2_) dissolved in 0.5 mL C_6_D_6_, was added 1.0 mmol, 0.02 mL H_2_O. The reaction was stirred for 5 min at 22°C and was analyzed by ^1^H and ^29^Si{^1^H} NMR spectroscopy.

#### 1:1 reaction of 2a and 1b

A mixture of 1 equiv. **2a**, 1 equiv. **1b** and 5.0 mol% (C_6_F_5_)_3_B(OH_2_) was stirred for 1 h at 22°C and was analyzed by ^1^H and ^29^Si{^1^H} NMR spectroscopy.

#### 1:1 reaction of 1b and 2b

A mixture of 1 equiv. **1b**, 1 equiv. **2b** and 5.0 mol% (C_6_F_5_)_3_B(OH_2_) was stirred for 5 min at 22°C and was analyzed by ^1^H and ^29^Si{^1^H} NMR spectroscopy.

### Control Reactions

A mixture of silanol (Et_3_SiOH, **2a**, 2.6 mmol, 0.40 mL or PhMe_2_SiOH, **2b**, 2.6 mmol, 0.40 mL) and 0.1–0.5 mol% (C_6_F_5_)_3_B(OH_2_) was stirred and allowed to react at room temperature (22°C). The reaction was monitored using ^1^H and ^29^Si{^1^H} NMR spectroscopy at a specific time interval.

### Catalyst Recycling Studies

#### 3a

To 0.1–5.0 mol% (C_6_F_5_)_3_B(OH_2_) was added 5.0 mmol, 0.80 mL Et_3_SiH while stirring at room temperature (22°C). The reaction was monitored using ^1^H NMR after 1 and 3 h. At the end of the 3 h period, each product from different catalyst loading was isolated by extraction with pentane while recovering back the (C_6_F_5_)_3_B(OH_2_) catalyst used. The recovered catalyst at 1.0 mol% loading was recycled and re-used 4 times to give a total of 5 cycles and 5 isolated yields for Et_3_SiOSiEt_3_.

#### 3b

To 0.1–5.0 mol% (C_6_F_5_)_3_B(OH_2_) was added 2.0 mmol, 0.31 mL PhMe_2_SiH while stirring at room temperature (22°C). The reaction was monitored using ^1^H NMR after 1 h. At the end of the 1 h period, each product from different catalyst loading was isolated by extraction with pentane. At 1.0 mol% catalyst loading, another 0.31 mL (2.0 mmol) of PhMe_2_SiH was added to the same vial. This procedure was done 4 times at 1 h intervals. The reported % yield at 1.0 mol% catalyst loading was the average of 5 cycles.

#### 3c

To 0.1–5.0 mol% (C_6_F_5_)_3_B(OH_2_) was added 1.0 mmol, 0.26 g Ph_3_SiH, dissolved in 0.50 mL toluene, while stirring at room temperature (22°C). The reaction was monitored using ^1^H NMR after 1 and 2 h. At the end of the 2 h period, each product from different catalyst loading was isolated by extraction with dichloromethane (5 mL). At 1.0 mol% catalyst loading, another 0.26 g (1.0 mmol) of Ph_3_SiH dissolved in 0.50 mL toluene was added to the same vial. This procedure was done 4 times at a 2 h intervals. The reported % yield at 1.0 mol% catalyst loading was the average of 5 cycles.

## Results and Discussion

### Intermolecular Synthesis of Oligosiloxanes Using a (C_6_F_5_)_3_B(OH_2_) Catalyst

**Scheme 2:**
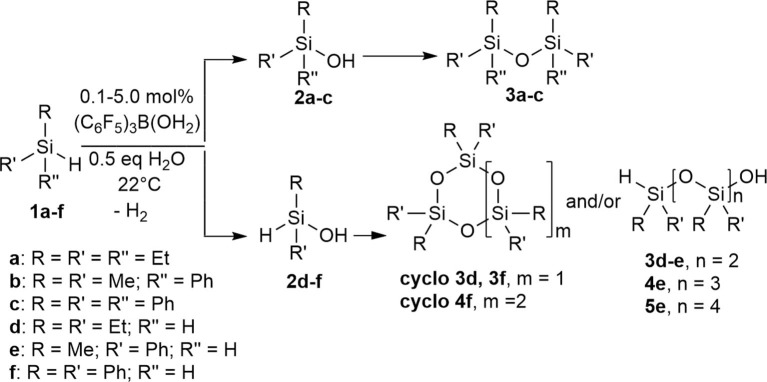
Intermolecular synthesis of siloxanes using a (C_6_F_5_)_3_B(OH_2_) catalyst.

Three different types of tertiary hydrosilanes (Et_3_SiH, **1a**; PhMe_2_SiH, **1b**; and Ph_3_SiH, **1c**) were reacted with varying equivalents of H_2_O (0.0–1.0) and varying concentrations of catalyst (0.1–10.0 mol %), at 22°C to yield the corresponding disiloxane (**3a-c**; Scheme 2). For each completed reaction, the catalyst was removed by Florisil filtration. For **1a** and **1b**, the siloxane product was isolated by dissolving the reaction mixture in *n*-pentane to selectively precipitate out the (C_6_F_5_)_3_B(OH_2_) catalyst. **1c** is a solid so the catalytic reactions were enabled by the addition of minimum amount of dry toluene (~1 mL). The disiloxane products formed (**3a-3c**) from this synthetic route were isolated and confirmed by several characterization techniques in conjunction (Supplementary Figures 1–28) (Jorapur and Shimada, 2012). Generally, the complete conversion to disiloxane is much slower in the absence of H_2_O and no reaction can be observed in the absence of the catalyst (Table 1). The transformation of neat **1b** to the corresponding disiloxane appeared to be the fastest and most facile at 0.1 mol% catalyst loading. This may be attributed to the fact that (C_6_F_5_)_3_B(OH_2_) is quite soluble in **1b**.

**Table 1:** Scope of substrates and products in the synthesis of disiloxanes, **3a-3c**.

**Substrate**	**Catalyst, mol %**	**H_**2**_O**,	**Reaction time, h**	**Major species present**	**Other species present**	**Yield, %**
		**equivalence**				
Et_3_SiH	5.0	0.5	70	**3a**	–	71
**1a**	1.0	0.5	70	**3a**	–	73
	1.0	0.5	24	**3a**	–	72
	1.0	0.5	1	**3a**	–	69
	0.1	0.5	1	**1a**	**3a**, **2a**	*
	0.1	0.5	3	**1a**	**3a**, **2a**	*
	0.1	0.5	24	**1a**, **3a**	**2a**	*
	0.1	0.5	48	**3a**	**2a**	*
	0.0	1.0	24	No reaction
PhMe_2_H	5.0	0.5	70	**3b**	–	78
**1b**	1.0	0.2	1	**3b**	**1b**, **2b**	*
	1.0	0.2	3	**3b**	**1b**, **2b**	*
	1.0	0.2	72	**3b**	**1b**, **2b**	*
	1.0	Excess	1	**3b**	–	*
	1.0	Excess	24	**3b**	–	*
	0.5	0.5	1	**3b**	–	*
	0.5	0.5	3	**3b**	–	76
	0.1	0.5	1	**3b**	–	*
	0.1	0.5	3	**3b**	–	77
Ph_3_SiH	5.0	0.0	70	**3c**	**3b**	*
**1c**	5.0	0.5	70	**3c**	–	75

* *% yield of **3a-3c** was not determined as it was a mixture*.

The reaction of **1a** was slow at a low catalyst loading, 0.1 mol% (C_6_F_5_)_3_B(OH_2_), in the presence of 0.5 eq H_2_O. However, this allowed the for the detection of the silanol intermediate, Et_3_SiOH, **2a**, along with the formation of the disiloxane, Et_3_SiOSiEt_3_, **3a** (Supplementary Figures 6, 7). After 3 h, the formation of both **2a** (δ_Si_ = 19.8 ppm) and **3a** (δ_Si_ = 8.9 ppm) was more evident with a significant amount of unreacted **1a** (δ_Si_ = 0.01 ppm). By contrast, the reaction was almost complete after 1 h when the catalyst loading was increased to 1.0 mol% (C_6_F_5_)_3_B(OH_2_), with >99% conversion and the **3a** as the major product. There was no significant change observed when the same reaction was left to react further for 24 h (Supplementary Figures 8, 9). When the catalyst loading was further increased to 5.0 mol% (C_6_F_5_)_3_B(OH_2_) the results were the same as those found at the 1.0 mol% catalyst loading. As suggested by the Piers-Rubinsztajn mechanism, the condensation of silanols to form oligo- or polysiloxanes occurs at relatively low catalyst concentration (Brook, 2018). A control reaction wherein **1a** was reacted with 1.0 eq H_2_O in the absence of the catalyst gave no reaction, showing only the presence of unreacted **1a** by NMR spectroscopy. Similarly, no reaction was observed when **2a** was reacted with an equivalent of H_2_O without a catalyst. These studies prove that both the conversion of **1a** to **2a** and the condensation step to **3a** require a catalyst (Supplementary Figures 10, 11).

Staring from a low catalyst loading of 0.1 mol% (C_6_F_5_)_3_B(OH_2_) in the presence of 0.5 eq H_2_O, the conversion of PhMe_2_SiH, **1b**, to the corresponding disiloxane, **3b** (δ_Si_ = 0.01 ppm) was already evident after only 1 h reaction time. Even when the catalyst concentration was further increased to 0.5 and 5.0 mol%, the reaction proceeded in the same manner as when using a lower amount of catalyst (Supplementary Figures 17, 18). Decreasing the amount of H_2_O to 0.2 eq at 1.0 mol% (C_6_F_5_)_3_B(OH_2_) left some of the starting material, **1b**, and presence of the silanol, PhMe_2_SiOH (**2b**), was also observed (Supplementary Figures 20, 21). It can be inferred that the addition of H_2_O plays an important role in the reaction, especially, in terms of the length of time needed to complete the reaction. Conversely, using an excess amount of H_2_O hastened the reaction while using the same catalyst concentration of 1.0 mol% (C_6_F_5_)_3_B(OH_2_). The reaction was observed to have been completed after 1 h (Supplementary Figure 22).

To further investigate the effect of reaction time and the necessity of H_2_O, the direct synthesis of Ph_3_SiOSiPh_3_, **3c**, was conducted at 5.0 mol% (C_6_F_5_)_3_B(OH_2_), for 70 h with and without the addition of H_2_O. It was observed that for reactions left for a longer period of time, the addition of H_2_O seemed to be not necessary, and the presence of the catalyst (C_6_F_5_)_3_B(OH_2_), alone is sufficient enough to yield the desired product. As previously observed with the other silanes, a mixture of Ph_3_SiH (**1c**) with H_2_O in the absence of the catalyst did not show any sign of a reaction (Supplementary Figures 27, 28).

Using the same protocol as for the tertiary silanes (**1a-c**), the reaction of secondary hydrosilanes (**1d-f**) was performed and generally resulted to the direct synthesis of oligomeric siloxanes with 3-5 repeat units identified based on ESI-MS. Three different secondary silanes were used: Et_2_SiH_2_, **1d**, PhMeSiH_2_, **1e**, and Ph_2_SiH_2_, **1f**. Depending on the substrate used, the products were observed to be linear and/or cyclic siloxane chains (Scheme 2; Supplementary Figures 29–31). Furthermore, in the case of **1e** there was evidence of siloxanediol formation (Diemoz et al., 2016) indicating its role as an intermediate in the synthesis of the higher siloxanes under these conditions.

Similar to the synthesis of symmetrical disiloxanes, the reaction proceeded at a faster rate in the presence of H_2_O. The reaction with **1e** gave mostly linear oligomers containing 3-5 repeat units, with a minor amount of oligosiloxanes containing 6-7 repeat units. The oligomerization of dialkylsilane, **1d**, resulted to both linear and cyclic products with 3 Si-O units, while the diarylsilane, **1f**, resulted to cyclic oligosiloxanes with *n* = 3 and 4. For **1d**, it is interesting to note that after 48 h it forms a cyclic species but if left for longer reverts back to the linear species (Table 2).

**Table 2:** Scope of substrates and products in the synthesis of oligosiloxanes, **cyclo 3d, 3f**, **4f**, and **linear 3d-3e, 4e, 5e**.

**Substrate**	**Catalyst, mol%**	**H_**2**_O**,	**Reaction time, h**	**Major species present**	**Other (or minor) species present**
		**equivalence**			
Et_2_SiH_2_	2.0	1.0	1	**3d**	**cyclo 3d**
**1d**	2.0	1.0	6	**3d**	**cyclo 3d**
	2.0	1.0	24	**3d**	**cyclo 3d**
	2.0	1.0	48	**cyclo 3d**	**3d**
	2.0	0.0	96	**3d**	–
PhMeSiH_2_	2.0	1.0	1	**3e**	–
**1e**	2.0	1.0	6	**3e**	–
	2.0	1.0	24	**3e, 4e**	**5e**
	2.0	1.0	48	**3e**, **4e**	**5e**
	2.0	0.0	96	**3e**	–
Ph_2_SiH_2_ **1f**	2.0	1.0	70	**cyclo 3f**	**cyclo 4f**

### Intramolecular Synthesis of Tethered Siloxanes Using a (C_6_F_5_)_3_B(OH_2_) Catalyst

We previously reported a preliminary study on the preparation of symmetrical naphthalene bridged disilanes with a Si-O-Si motif (Rabanzo-Castillo et al., 2019). Having success in forming linear siloxanes from tertiary and secondary silanes, it intrigued us to perform intramolecular reactions with catalytic amounts of (C_6_F_5_)_3_B(OH_2_) to produce tethered and unsymmetrical siloxanes. In order to perform such reactions, disilyl precursors (**7a-c**) that resemble tertiary silanes having Si-H bonds were prepared using Grignard reactions from dibrominated bridge precursors (**6a-b**; Scheme 3). The synthesized disilyl tertiary silane precursors were obtained in high yields (Supplementary Figures 32–44) and were later subjected to intramolecular reactions with the catalyst under aerobic conditions to obtain the desired tethered siloxanes (Supplementary Figures 45–57), cyclic structures with 6-7 membered rings (**8a-c**; Scheme 3).

**Scheme 3:**
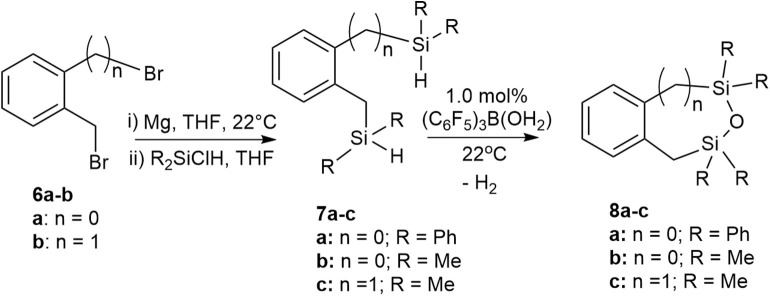
Intramolecular synthesis of symmetric and asymmetric tethered siloxanes using a (C_6_F_5_)_3_B(OH_2_) catalyst.

The intramolecular dehydrocoupling step was optimized for each of the disilyl tertiary silane precursors. **7a** (δ_Si_ = −13.7, −22.5 ppm) is a solid therefore was dissolved in toluene prior to the intramolecular dehydrocoupling using 5.0 mol% (C_6_F_5_)_3_B(OH_2_) at 90°C for 24 h. Under these conditions, the only product observed was **8a** (δ_Si_ = −9.9, −14.1 ppm). The disilyl tertiary silane precursors **7b** (δ_Si_ = −11.0, −21.6 ppm) and **7c** (δ_Si_ = −13.3) are liquids and therefore were able to undergo intramolecular dehydrocoupling reactions without the addition of solvents, but the catalysis was more efficient with the addition of toluene along with 1.0 mol% (C_6_F_5_)_3_B(OH_2_) at 22°C for 3 h, yielding **8b** (δ_Si_ = 10.9, 3.7 ppm) and **8c** (δ_Si_ = 7.2), respectively. The crude siloxanes (**8a-c**) were filtered through a Florisil pad to remove the catalyst. Although cyclic siloxanes are known to degrade when subjected to purification by silica gel column chromatography, compounds **8a-8c** could be purified by column chromatography, using hexanes as an eluent (Blackwell et al., 1999). The ^29^Si{^1^H} NMR chemical shifts of the purified product matched that of the filtered product suggesting that, in this case, the products do not degrade on silica gel (Supplementary Figures 45–57).

**Figure 1:**
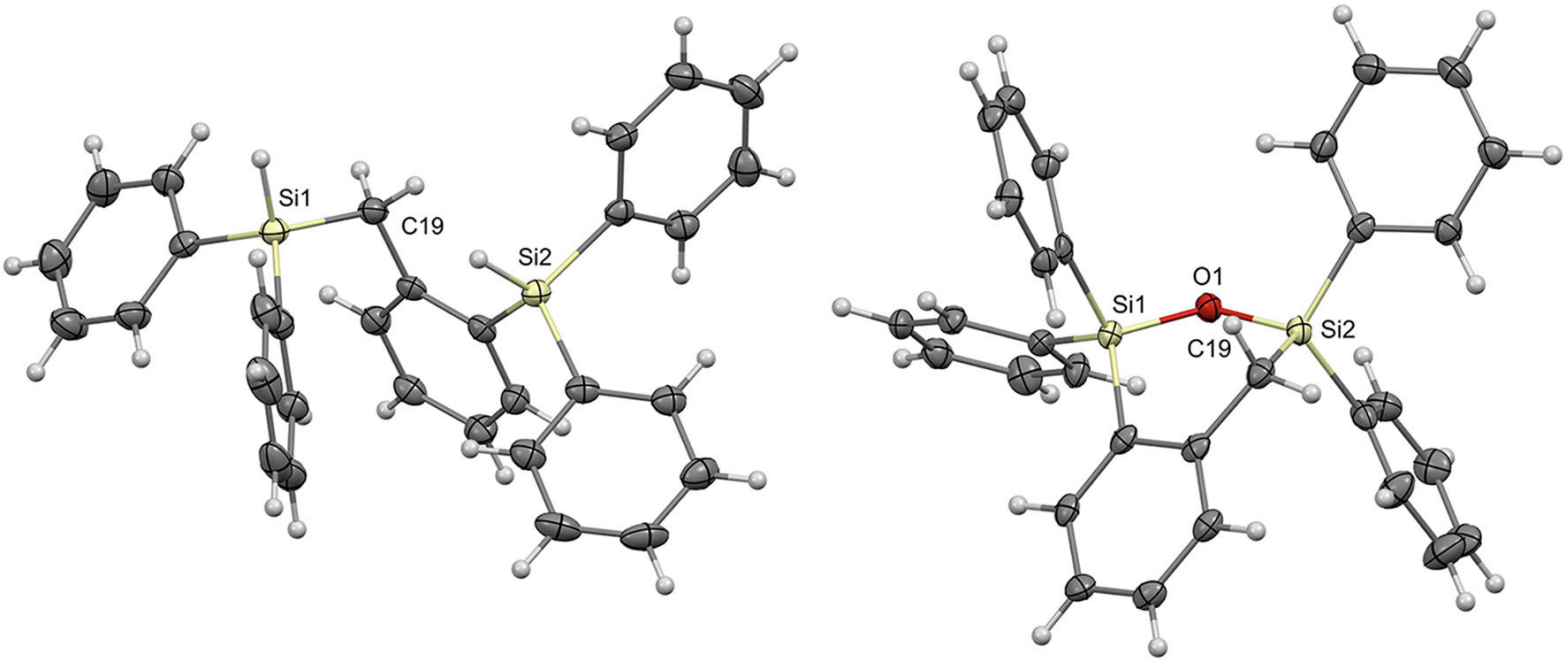
Molecular structures of **7a (Left)** and **8a (Right)** with thermal ellipsoids drawn at the 50% probability level.

Single crystals were grown for compounds **7a** and **8a** by slow evaporation of pentane and ethyl acetate, respectively; the crystal structures were solved by single crystal X-ray diffraction (Figure 1; Supplementary Table 1). From the molecular structure of product **7a** it is evident that due to the steric bulk from the phenyl groups on the silyl substituents and the flexibility at C19, the Si-H bonds are quite far from each other. The two silicon atoms are 4.292 Å apart. The molecular structure of **8a** shows that the newly formed 6-membered cyclic structure, containing the Si1-O1-Si2 bond, results in a slightly distorted boat shaped geometry with Si1 and C19 as the central two atoms. The distortion can be attributed to the presence of the Si-O-Si linkage and phenyl substituents lengthening the bonds through these atoms in addition to the wider Si-O-Si bond angle of 124.41°.

### Mechanistic Considerations

**Scheme 4:**
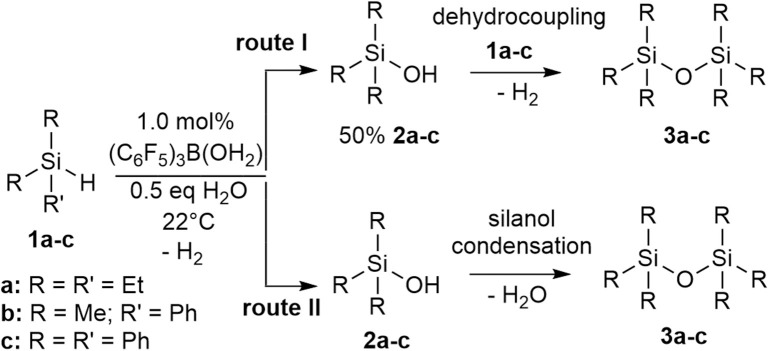
Two potential routes to disiloxane from silane via silanol.

Looking at the simplest system, using the tertiary silanes (**1a-c**), the mechanism of the catalysis was investigated. The reaction process involves the formation of the silanol intermediate and generation of either hydrogen gas or H_2_O to give the disiloxanes, **3a-c**, depending on which pathway is operating (**route I** or **route II**; Scheme 4). It is important to note that the two pathways are experimentally distinct. Creation of **2** from **1** involves the loss of H_2_. Similarly, with a 50% conversion to **2**, the silanol (**2**) can react with the hydrosilane (**1**) to form the disiloxane (**3**) with concomitant loss of H_2_ in a dehydrocoupling reaction (**route I**; Scheme 4). However, if all of **1** becomes **2** (more likely under higher concentrations of H_2_O), then the subsequent condensation of the silanols eliminates H_2_O in a condensation reaction to form a disiloxane (**route II**; Scheme 4).

Kinetic studies were attempted to better probe the postulated mechanism, however, were not possible due to the difficulty in matching the experimental conditions by NMR spectroscopy. Moreover, each individual reactant (**1a-c**) gave reactions that were too fast with (C_6_F_5_)_3_B(OH_2_). To elaborate the routes occurring, neat reactions of individual silanols (**2a**, **2b**), under the same reaction conditions of the hydrosilanes, were also performed. Hydrosilanes (**1**, R_3_SiH) are said to be more susceptible to Brønsted acid-catalyzed reactions (Muzafarov, 2011) which is generally what was observed (Table 3). Interestingly, the addition of 0.5 eq. of H_2_O increased the rate of reaction of **1a** to **3a**, from 50% conversion to >99% after 1 h, while the same addition of H_2_O did not have an impact on the already rapid conversion of **1b** to **3b**. For the case of a catalytic reaction starting with Et_3_SiOH, **2a**, the reaction took longer than the reaction starting with **1a** to reach completion (24 and 3 h, respectively). However, the same reaction starting with PhMe_2_SiOH, **2b**, proceeded to completion within an hour. Limiting air in the reactions starting with the silanol (e.g., **2a** and **2b**) only made a significant difference in the reaction rate for **2a**; **2b** was unaffected. When a 1:1 mixture of PhMe_2_SiH, **1b**, and PhMe_2_SiOH, **2b**, were reacted, a rapid exotherm and build-up of pressure was observed resulting to almost full conversion after only a few seconds (Table 3). These results suggest, not only that the reaction with **1b** is the most rapid in the series, but also, that both routes to form the disiloxane are possible under the catalytic conditions.

**Table 3:** Comparative reactivities of SiH and SiOH groups in the presence of catalytic (C_6_F_5_)_3_B(OH_2_).

**(C_**6**_F_**5**_)_**3**_B(OH_**2**_) +**	**Reaction time, h**	**Conversion, %**
**1a** (in air)	1 h	50
**1a** (in air)	3 h	>99
**1b** (in air)	1 h	>99
**1a** (in air) + 0.5 eq H_2_O	1 h	>99
**1b** (in air) + 0.5 eq H_2_O	1 h	>99
**2a** (in air)	1 h	No reaction
**2a** (in air)	24 h	>99
**2a** (limited air)	24 h	No reaction
**2b** (in air)	1 h	>99
**2b** (limited air)	24 h	>99
**1b** + **2b** (in air)	Immediate	>99
**1b** + **2a** (in air)	1 h	>99

To elaborate on the relative rates of reaction for substrates **1a-c**, and to have a better indication of chemoselectivity, a cross-condensation reaction of a 1:1:1 mixture of Ph_3_SiH (**1c**), Et_3_SiH (**1a**), and H_2_O was performed with 1 mol% catalyst loading. The reaction resulted in the rapid evolution of H_2_ and was observed to reach completion soon after the addition of H_2_O into the reaction mixture (Supplementary Figures 58–61). As expected, the reaction resulted to three different products—two homo-coupling products, Ph_3_SiOSiPh_3_ (**3c**) and Et_3_SiOSiEt_3_ (**3a**) and a cross-coupling product, Ph_3_SiOSiEt_3._ A crude estimate using the ^1^H NMR spectrum of the product mixture revealed a ratio of 2.5 **3c:** 1 Ph_3_SiOSiEt_3:_ 2 **3a**, instead of a 1:1:1 ratio which would be expected if the **1a** and **1c** reacted at the same rate. This deviation can be explained by the difference in kinetics and solubilities of the two reactants. **1c** alone and **1a** alone require 2 and 3 h reaction time, respectively, to reach full conversion, while **1b** is even more rapid at 1 h. To further confirm this claim, cross-condensation reaction between **1b** and **1a** (Supplementary Figure 62) resulted to **3b** and PhMe_2_SiOSiEt_3_ as the major products in a 1:6 ratio and, no **3a** was observed. Therefore, the reactivity to form disiloxanes (**3a-c**) from the three tertiary silane substrates under the catalytic conditions follows the trend **1b** > **1c** > **1a**.

Cross-coupling between PhMe_2_SiH, **1b**, and Et_3_SiOH, **2a**, on the other hand, resulted to a 1:1:1 ratio of **3b** to the cross product, PhMe_2_SiOSiEt_3_ to **3a**. In this case, both homo-coupling products were obtained along with the expected unsymmetrical cross-coupling product (Supplementary Figures 63, 64) and the ratio suggests that the difference in the aforementioned reactivity trend is mostly likely due to the rate determining formation of the silanol (**2**) from the silane (**1**), which is most sluggish for the conversion of **1a** to **2a**.

Monitoring the catalytic intermediates and the formation of silanol experimentally *in situ* in order to capture the impact of ppm-level changes of the substrates was challenging. Nevertheless, NMR tube reactions wherein 2.6 mmol each of **1b** and **2b** were reacted, separately, with 0.1 mol% of (C_6_F_5_)_3_B(OH_2_). Experimentally, partial conversion to **2b** and **3b** after 4 h was observed with **1b**, which is significantly slower compared to the reaction performed with constant stirring. It is interesting to note that when using a hydrosilane (e.g., **1b**) as the starting material, the products that have been formed (i.e., **2b** and **3b**) after 4 h tend to revert back to PhMe_2_SiH after letting the reaction stand for a total of 96 h (Supplementary Figure 65). This indicates that the reaction is not quite as simple as implied, with reversibility at play along with the direct siloxane formation. In contrast, using the silanol (**2b**) as the starting material, full conversion was observed after 24 h and the product (**3b**) did not convert back to the silanol substrate after 96 h (Supplementary Figure 66).

**Scheme 5:**
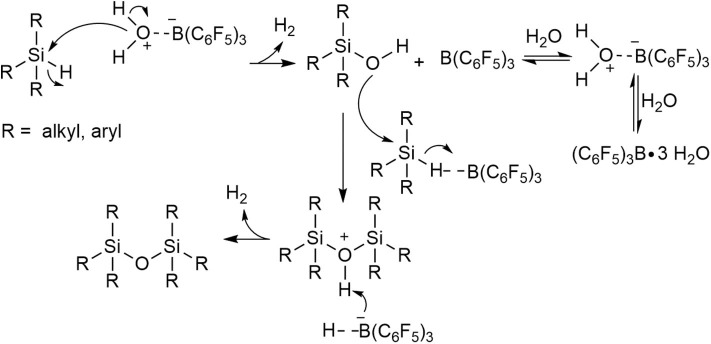
Proposed mechanism for the catalytic formation of disiloxanes using (C_6_F_5_)_3_B(OH_2_).

In summary, the results obtained from cross-coupling and control reactions indicate that the prevalent mechanism is where the hydrosilane (R_3_SiH) and silanol (R_3_SiOH) undergo dehydrocoupling to yield the disiloxane product (**3**), but under high concentration regimes of some silanol substrates (e.g., as confirmed for **2b**) the silanol condensation route is possible. The postulated reaction mechanism, therefore, for the transformation is a modified Pier-Rubinsztajn reaction (Scheme 5). The process involves: i) the hydrolysis of Si-H to Si-OH using (C_6_F_5_)_3_B(OH_2_) with subsequent release of H_2_ gas, followed by ii) nucleophilic attack of silanol (**2**, R_3_SiOH) to the hydrosilane (**1**, R_3_SiH) to form a disiloxane and another equivalent of H_2_ gas (Scheme 5).

In addition to the conversion of the three components (**1**, **2**, **3**) over time, the catalyst can have different interactions with the substrates (**1**, **2**) and H_2_O that are present during the reaction. Decomposition of (C_6_F_5_)_3_B(OH_2_) catalyst via B-C bond protonolysis is highly probable in strongly basic conditions and high temperatures (Bradley et al., 1996; Ashley et al., 2009; Scott et al., 2015). Generally, Frustrated Lewis Pairs (FLP's) demonstrate high sensitivity to moisture. The high Lewis acidity of B(C_6_F_5_)_3_ leads to strong complexation with H_2_O and deprotonation even with moderately strong bases can occur irreversibly (Bergquist et al., 2000). However, the method described in this article demonstrated moisture tolerance of the (C_6_F_5_)_3_B(OH_2_) catalyst as it was conducted at room temperature, and due to the lack of any strong base in a reaction with a hydrosilane as the sole substrate.

**Scheme 6:**
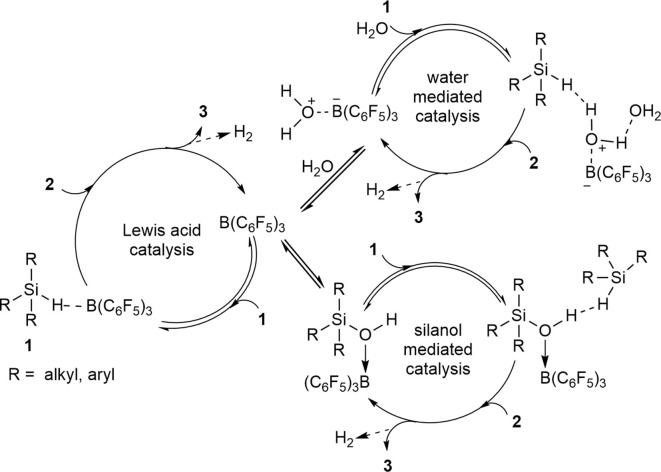
A catalytic mechanism with multiple competing catalytic pathways to form disiloxanes.

Another way to view the direct synthesis of disiloxanes is through a non-conventional mechanism which features multiple catalytically relevant species and series of competitive reactions (Scheme 6). The formation of disiloxanes can be regarded as a Lewis acid-catalyzed reaction, a water-mediated or a silanol-mediated type of catalysis (Yu et al., 2018). In the Lewis acid catalysis (Scheme 6), the Si-H bond of the silane is activated by B(C_6_F_5_)_3_ and then will further react with a silanol to produce the disiloxane with concomitant release of H_2_. By contrast, hydrosilylation reactions catalyzed by B(C_6_F_5_)_3_ are generally characterized by the formation of borane-silane complex (Parks and Piers, 1996; Parks et al., 2000; Rendler and Oestreich, 2008; Sakata and Fujimoto, 2013; Zhang et al., 2016; Cheng et al., 2018). Furthermore, several experimental and theoretical mechanistic studies have suggested that borane-catalyzed reactions of siloxanes are initiated by activation of the silane Si-H bond (Parks et al., 2000; Hog and Oestreich, 2009; Mewald and Oestreich, 2012; Sakata and Fujimoto, 2013; Mathew et al., 2017).

Since the B(C_6_F_5_)_3_ catalyst has strong affinity with H_2_O, the Lewis-acid mediated pathway (Scheme 6) is not enough on its own to describe the formation of the disiloxane. The reaction could also possibly proceed via a water-mediated catalytic cycle (Scheme 6). For this reaction pathway, R_3_SiH is initially activated by the (C_6_F_5_)_3_B(OH_2_) catalyst, which is in equilibrium with B(C_6_F_5_)_3_ in the presence of H_2_O. While B(C_6_F_5_)_3_ is a potent Lewis acid (C_6_F_5_)_3_B(OH_2_), can be regarded as a strong Brønsted acid (Bergquist et al., 2000). The Si-H activation step can lead to Lewis acidic silicon atom, which for this case then interacts with oxygen lone pair of the pre-formed silanol to generate **3** and eliminate H_2_. One final catalytic possibility, under higher concentrations of silanol (**2**) is that it can mediate the catalysis. Here the silanol (**2**) interacts with (C_6_F_5_)_3_B(OH_2_) catalyst via the oxygen atom which activates it toward a reaction with silane (**1**) to produce **3** and H_2_ while regenerating the catalyst/silanol adduct (Scheme 6).

The observations from the control and competition reactions suggest that the water-mediated catalysis (Scheme 6) is the most probable catalytic pathway which will lead to the formation of disiloxane. Nonetheless, there also might be a competition between more than one catalytic cycle happening simultaneously or under different concentration regimes of substrates **1** (e.g., toward the start of the reaction) and **2** (e.g., toward full conversion to **3**). Therefore, this seemingly simple reaction may have an incredibly complex mechanism.

### Catalyst Recycling Studies

Catalyst recycling, that is the reuse of the (C_6_F_5_)_3_B(OH_2_) catalyst, was considered to demonstrate the efficiency and sustainability of the direct synthetic route to oligosiloxanes. For this purpose, all experiments were conducted with constant stirring to allow efficient removal of hydrogen gas and favor the formation of disiloxane product. The reactions were monitored by ^1^H NMR spectroscopy to estimate the reaction time needed for each substrate to reach completion. From these studies, 3, 1 and 2 h reaction time was applied for Et_3_SiH (**1a**), PhMe_2_SiH (**1b**) and Ph_3_SiH (**1c**), respectively.

**Figure 2:**
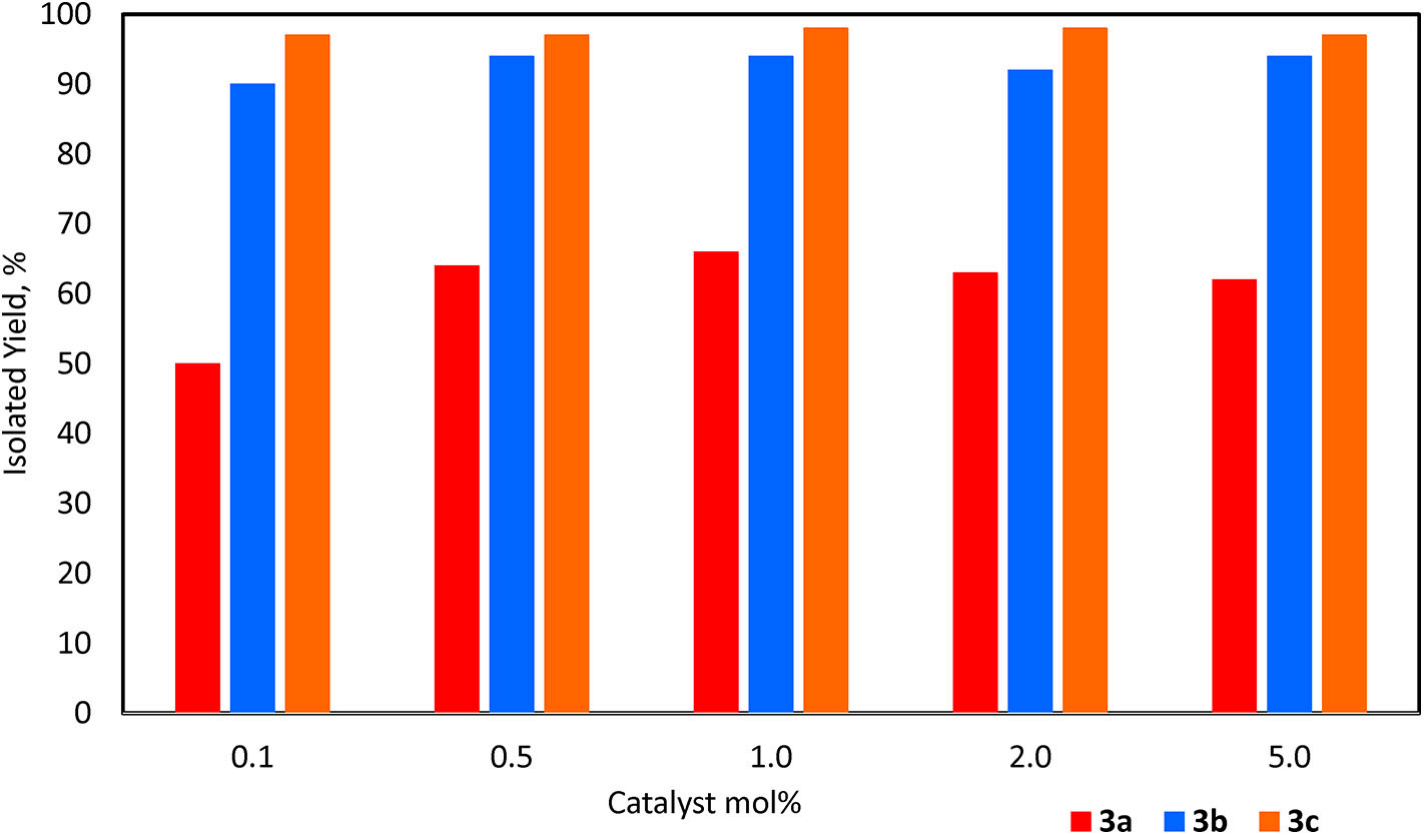
Yields for **3a-c** with catalyst loadings 0.1–5.0 mol%.

For Et_3_SiOSiEt_3_, **3a**, the catalyst was recovered conveniently as it settled at the bottom of the flask after the stirring was stopped. The product was isolated by addition of *n*-pentane followed by decantation using a cannula. Looking at catalyst loading (0.1–5.0 mol%) there was very little discrepancy in isolated yield over this range and the best TOF achieved was 167 h^−1^ with 0.1 mol% catalyst loading (Figure 2; Supplementary Figure 67; Supplementary Table 2). The dependence on catalyst loading for the reaction of **1b** to form PhMe_2_SiOSiMe_2_Ph, **3b**, is far less pronounced with the given experimental conditions allowing for an increase in TOF with a decrease in catalyst loading; 900 h^−1^ TOF was achieved at 0.1 mol% loading (**3b**, Figure 2; Supplementary Figure 68; Supplementary Table 3). The reaction was completed after 1 h as observed by ^1^H NMR spectroscopy. In this case, the (C_6_F_5_)_3_B(OH_2_) remained soluble even after the formation of **3b**. Therefore, due to the challenges of directly testing the catalyst recyclability on such a small scale (e.g., 0.1 mol% loading), the activity of the catalyst (at 1.0 mol% loading) was demonstrated by adding a constant amount of **1b** to the same flask every hour after testing the degree of completion by ^1^H and ^29^Si NMR spectroscopy. After 5 cycles, **3b** was isolated and the % yield for each trial was reported as the average of 5 cycles (94%). Similarly, using **1c** as the substrate, the reaction rate exhibited independence of catalyst loading with a TOF of 485 h^−1^ at 0.1 mol% loading (**3c**, Figure 2; Supplementary Figure 69; Supplementary Table 4). However, the reaction required the addition of toluene to dissolve the two solids (**1c** and catalyst). Similar to the reaction with **1b**, given that the reaction was performed in a mmol scale, a constant volume of 3.0 M solution of **1c** in toluene was added sequentially to the same flask at 1.0 mol% catalyst loading. The product Ph_3_SiOSiPh_3_, **3c**, was again isolated collectively at the end of the fifth cycle (98%).

**Figure 3:**
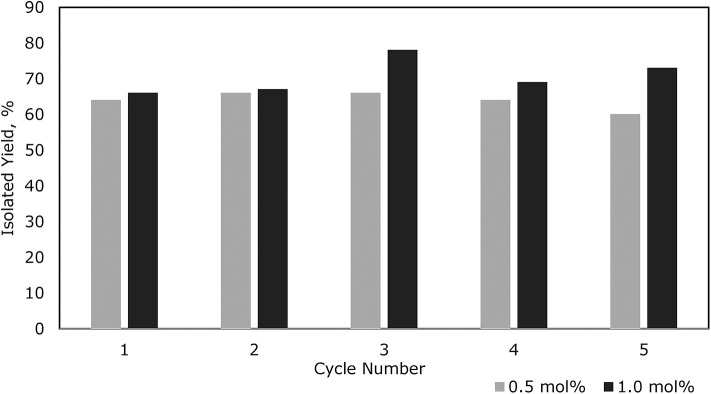
Yields for **3a** over 5 cycles of recycling using 0.5 and 1.0 mol% catalyst loading.

To demonstrate the recycling by separating the catalyst and product after each cycle, both 0.5 mol% and 1.0 mol% catalyst loadings of (C_6_F_5_)_3_B(OH_2_) were chosen. The results showed that the activity of the catalyst for each cycle is almost the same (within experimental error) for up to five repeats (Figure 3). The volatility and solubility of the substrate plays an important role in facilitating the formation of the disiloxane product in excellent yields. Since the reactions were performed with constant stirring, it can be assumed that some of the Et_3_SiH may have been lost prior to its conversion to product (see lower yield for **3a**, Figure 2). In addition, the solubility of the catalyst was enhanced in the presence of a solvent as compared to a neat reaction. Overall, these experiments successfully demonstrated the longevity of the (C_6_F_5_)_3_B(OH_2_) catalyst and that it is possible to recycle it at least five times in reactions with the given substrates.

## Conclusions

The study presented in this article has demonstrated an efficient route from secondary and tertiary hydrosilanes into the corresponding oligosiloxanes using a moisture-stable and recyclable (C_6_F_5_)_3_B(OH_2_) catalyst through either an intermolecular or intramolecular reaction depending on the substrate. Multiple catalytically relevant species and a series of competitive reactions are probable when considering the mechanism involved for the direct synthesis of siloxanes from only hydrosilanes and water. A single catalytic pathway is simply not enough to describe the formation of the disiloxane. The experimental results gathered from control studies suggest that the most feasible route to disiloxanes is where the Si-H is partially converted to Si-OH followed by a heterodehydrocoupling reaction of Si-H/Si-OH. Moreover, the cross-coupling results show promise for the selective synthesis of unsymmetrical siloxanes under controlled conditions directly from silanes or using a mixture of silanes and silanols or alcohols and this will be the subject of future studies in our group. Nonetheless, given the wide range of hydrosilanes that are commercially available, this protocol should provide an easy route to a large variety of oligosiloxanes.

## Data Availability Statement

The datasets generated for this study can be found in the CCDC repository (1987430, 1987431) or are included in article/Supplementary Material.

## Author Contributions

KR-C and VK designed and performed the experiments under the supervision of EL. TS refined the crystal structures. KR-C and VK wrote the paper with editorial support from EL.

## Conflict of Interest

The authors declare that the research was conducted in the absence of any commercial or financial relationships that could be construed as a potential conflict of interest.
