# Supplementary material for: Catalytic Synthesis of Oligosiloxanes Mediated by an Air Stable Catalyst, (C6F5)3B(OH2)
Source: Front Chem. 2020 Jun 23;8:477. doi: 10.3389/fchem.2020.00477 (PMC7325218; doi:10.3389/fchem.2020.00477)
Supplement: Supplementary file 1 [file Data_Sheet_1.pdf]

Supplementary Material

Catalytic synthesis of oligosiloxanes mediated by an air stable catalyst, $(C_6F_5)_3B(OH_2)$

Kristel M. Rabanzo-Castillo,[1,2] Vipin B. Kumar,[1,2] Tilo Söhnel,[1,2] and Erin M. Leitao*[1,2]

[1] School of Chemical Sciences, University of Auckland, Private Bag, 92019, Auckland 1142, New Zealand

[2] The MacDiarmid Institute for Advanced Materials and Nanotechnology, New Zealand

1.1 Supplementary Figures: spectra of synthesized compounds

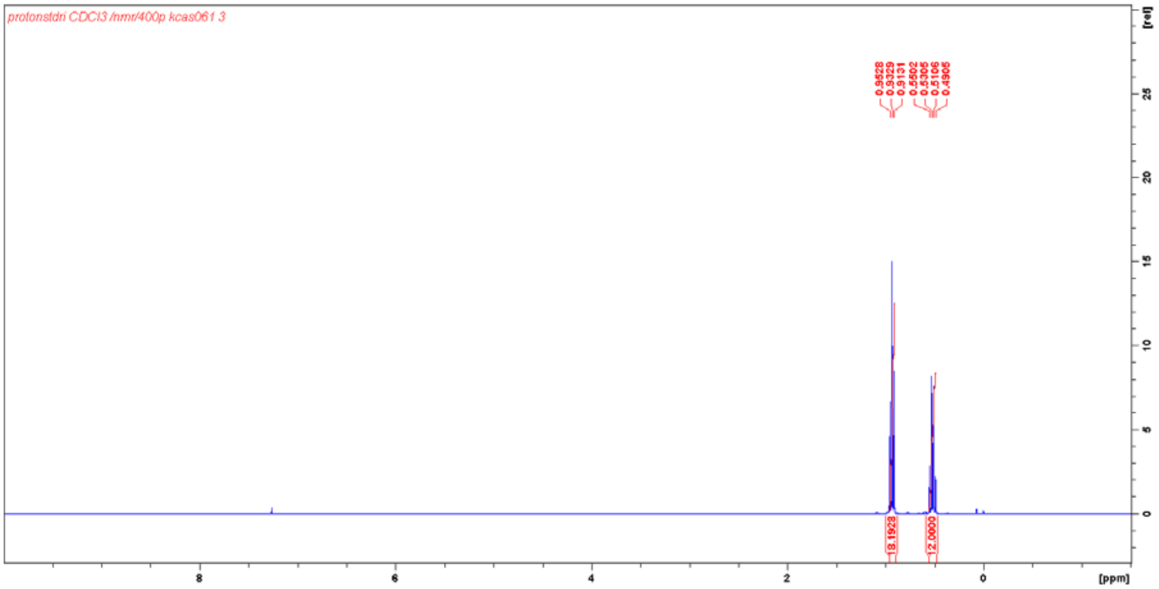

Supplementary Figure 1. ^{1}H NMR spectrum of $Et_3SiOSiEt_3$ (**3a**) in $CDCl_3$

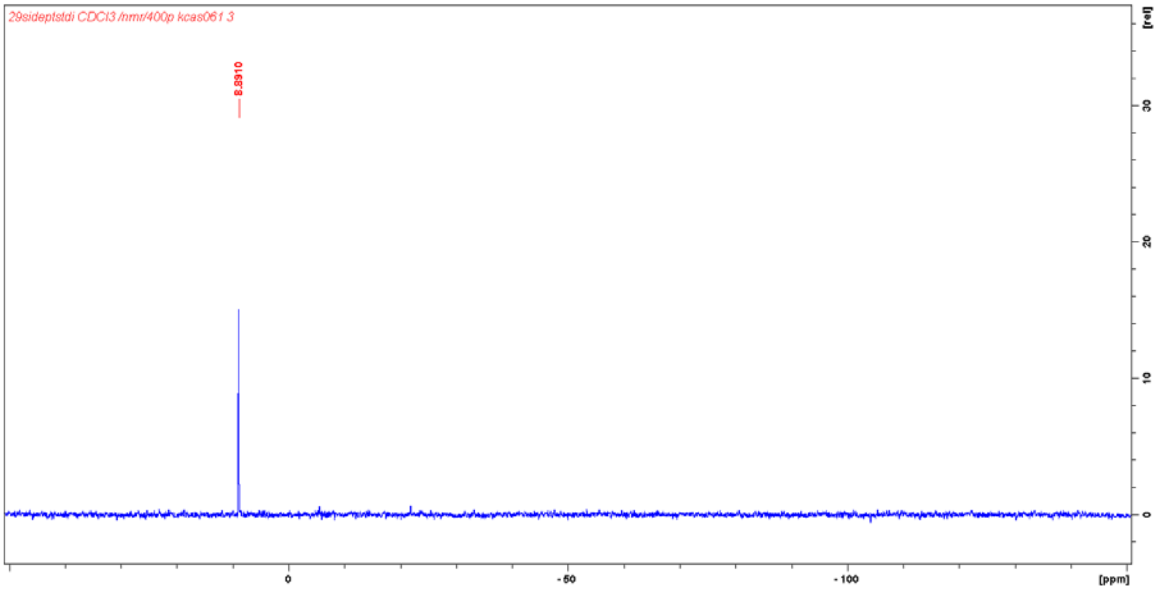

Supplementary Figure 2. $^{29}Si\{^{1}H\}$ NMR spectrum of $Et_3SiOSiEt_3$ (**3a**) in $CDCl_3$

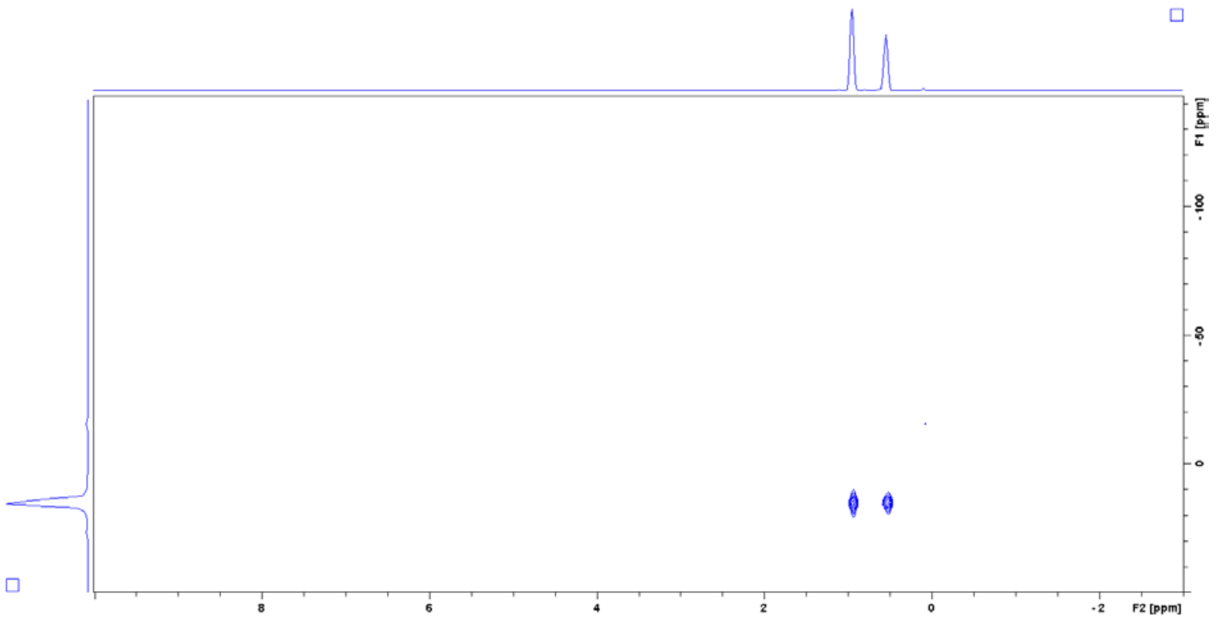

Supplementary Figure 3. $^{29}Si\{^1H\}$-HMBC NMR spectrum of $Et_3SiOSiEt_3$ (**3a**) in $CDCl_3$

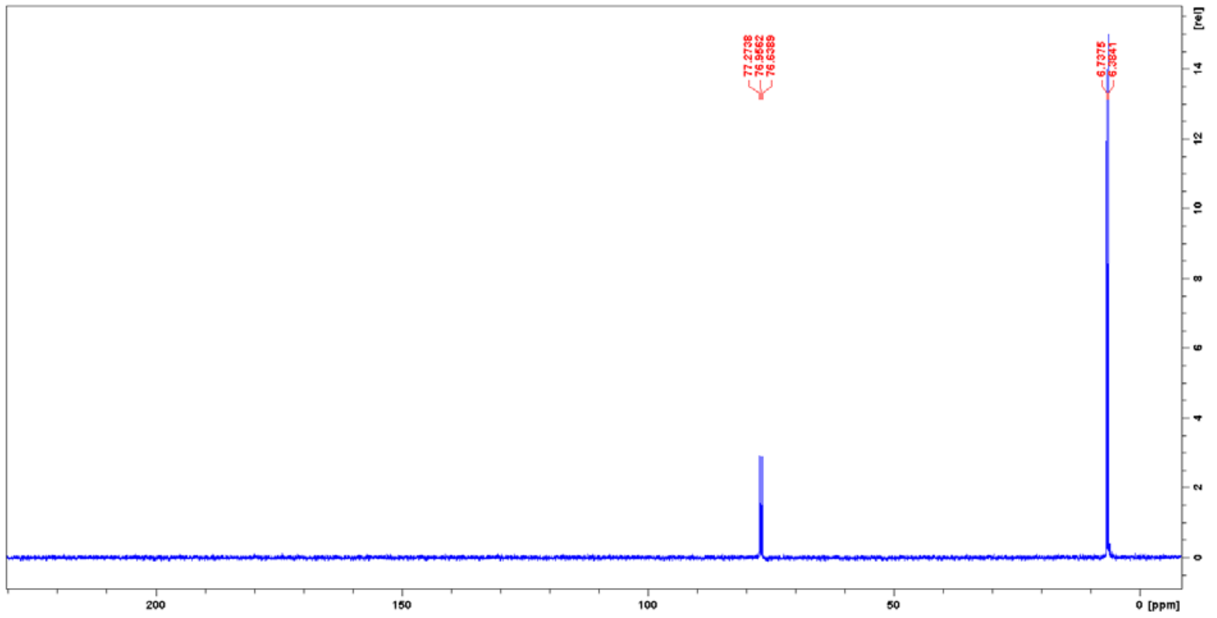

Supplementary Figure 4. $^{13}C\{^1H\}$ NMR spectrum of $Et_3SiOSiEt_3$ (**3a**) in $CDCl_3$

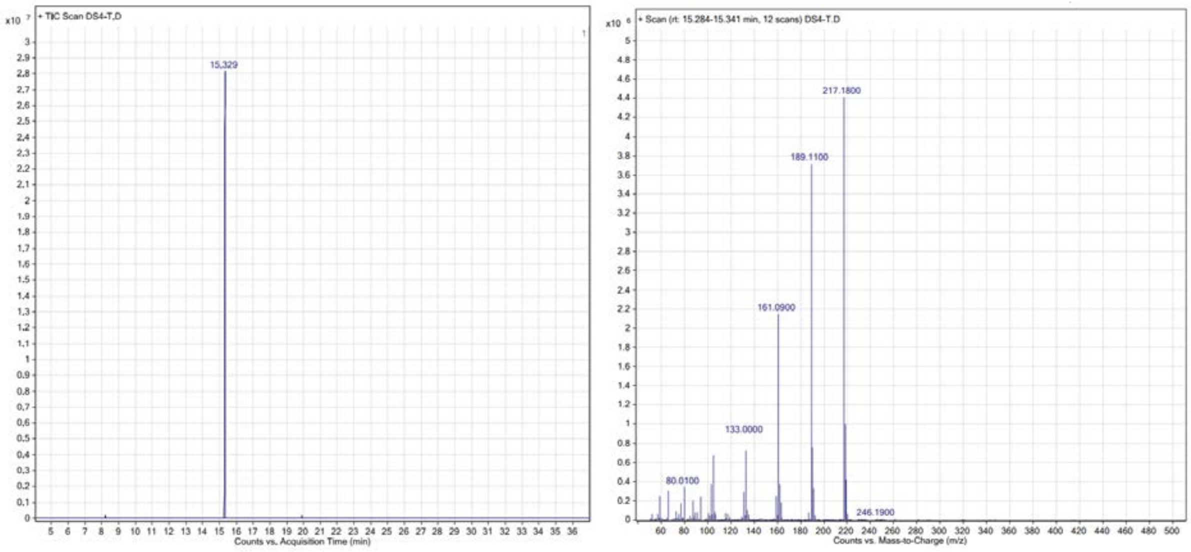

(a) (b)

Supplementary Figure 5. GC-MS of $Et_3SiOSiEt_3$ (**3a**) in $CDCl_3$ (a) counts vs acquisition, min (b) counts vs mass-to-charge, *m/z*

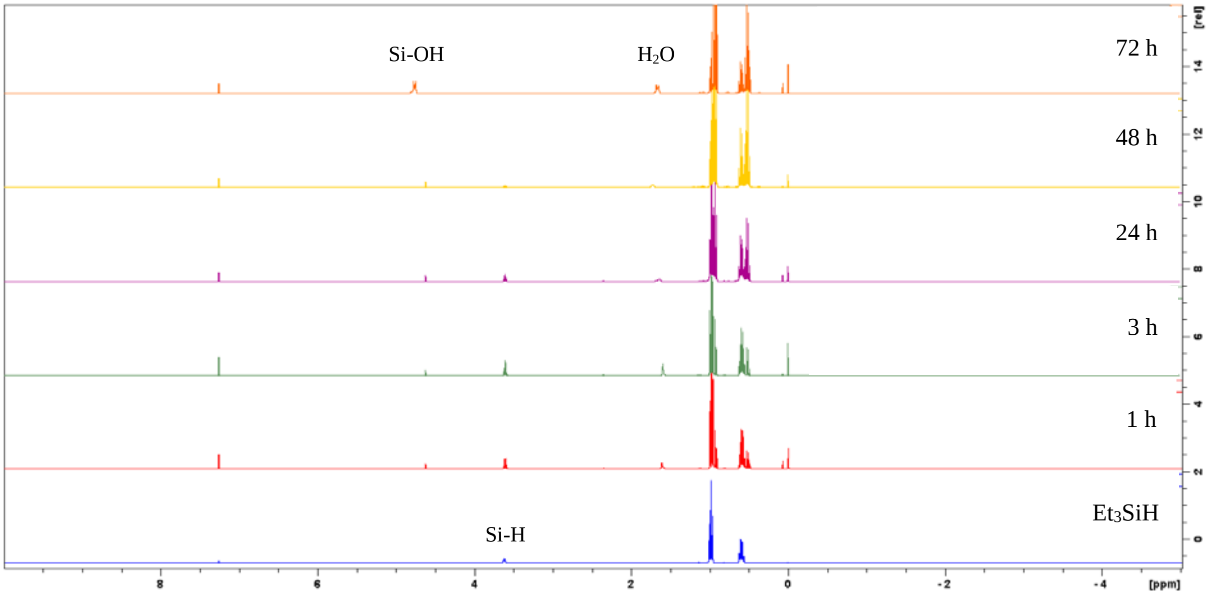

Supplementary Figure 6. ^{1}H NMR spectra of Et_3SiH (**1a**) after a reaction with 0.1 mol% $(C_6F_5)_3B(OH_2)$ and 0.5 eq H_2O in $CDCl_3$

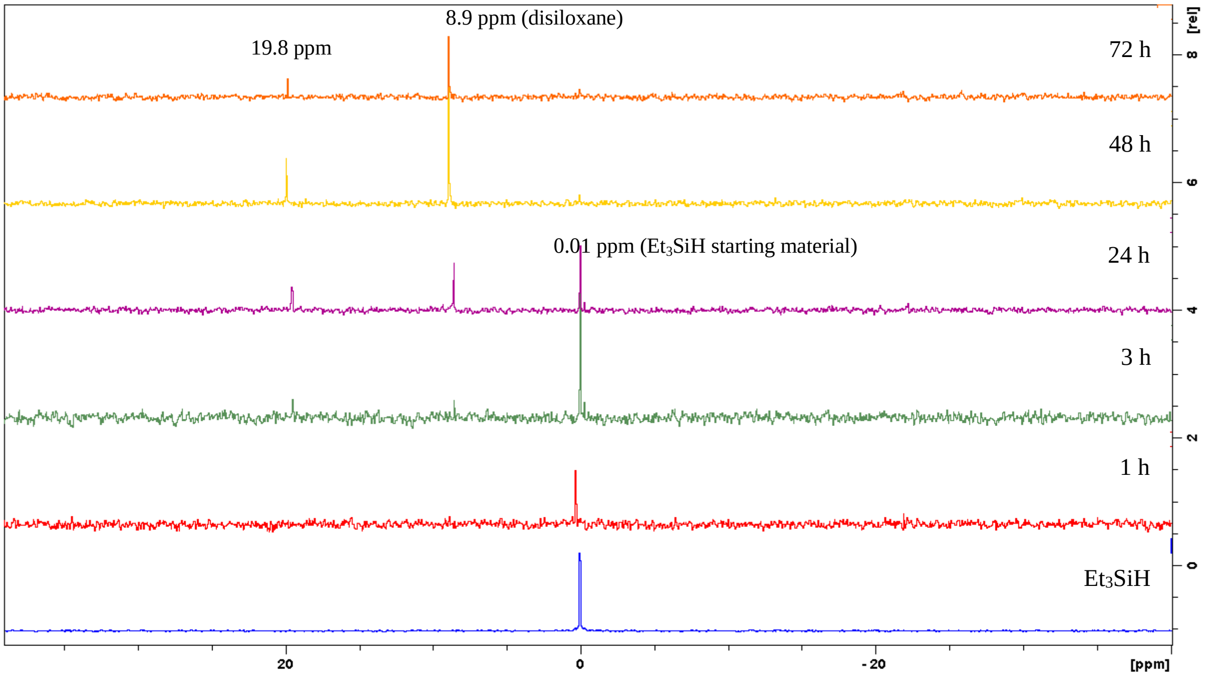

Supplementary Figure 7. $^{29}Si\{^{1}H\}$ NMR spectra of Et_3SiH (**1a**) after a reaction with 0.1 mol% $(C_6F_5)_3B(OH_2)$ and 0.5 eq H_2O in $CDCl_3$

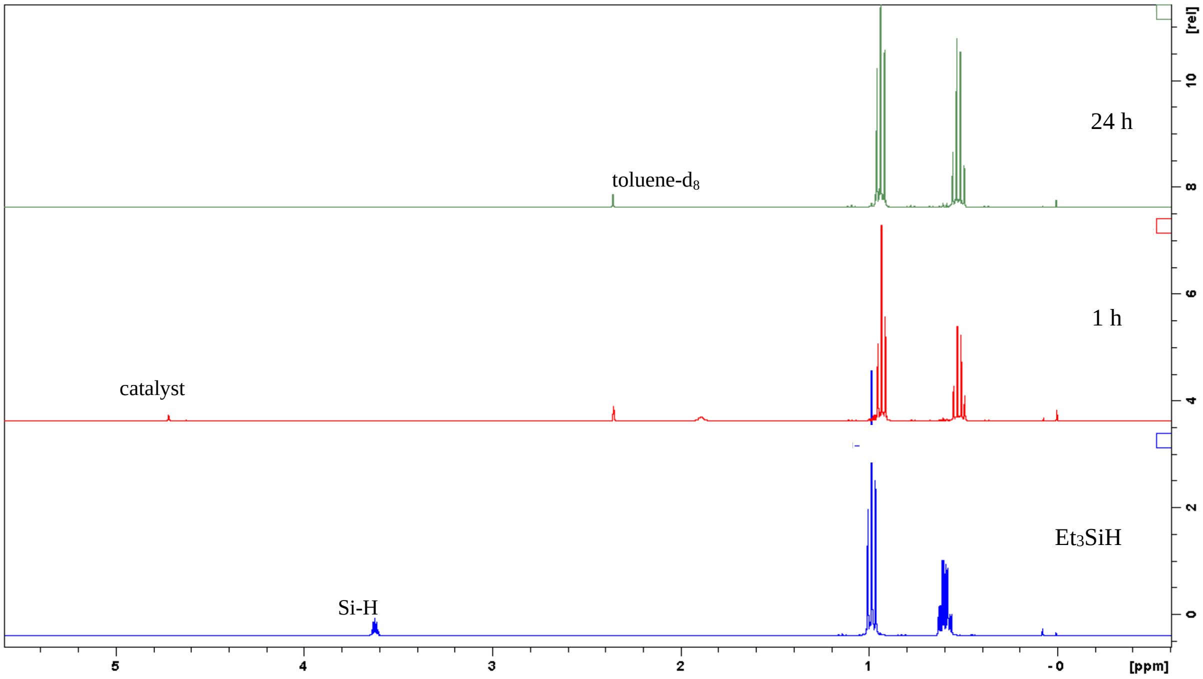

Supplementary Figure 8. ^{1}H NMR spectra of Et_3SiH (**1a**) (bottom, blue) after reacting with 1.0 mol% $(C_6F_5)_3B(OH_2)$ and 0.5 eq H_2O for 1 h (middle, red) and 24 h (top, green) in toluene-d_8

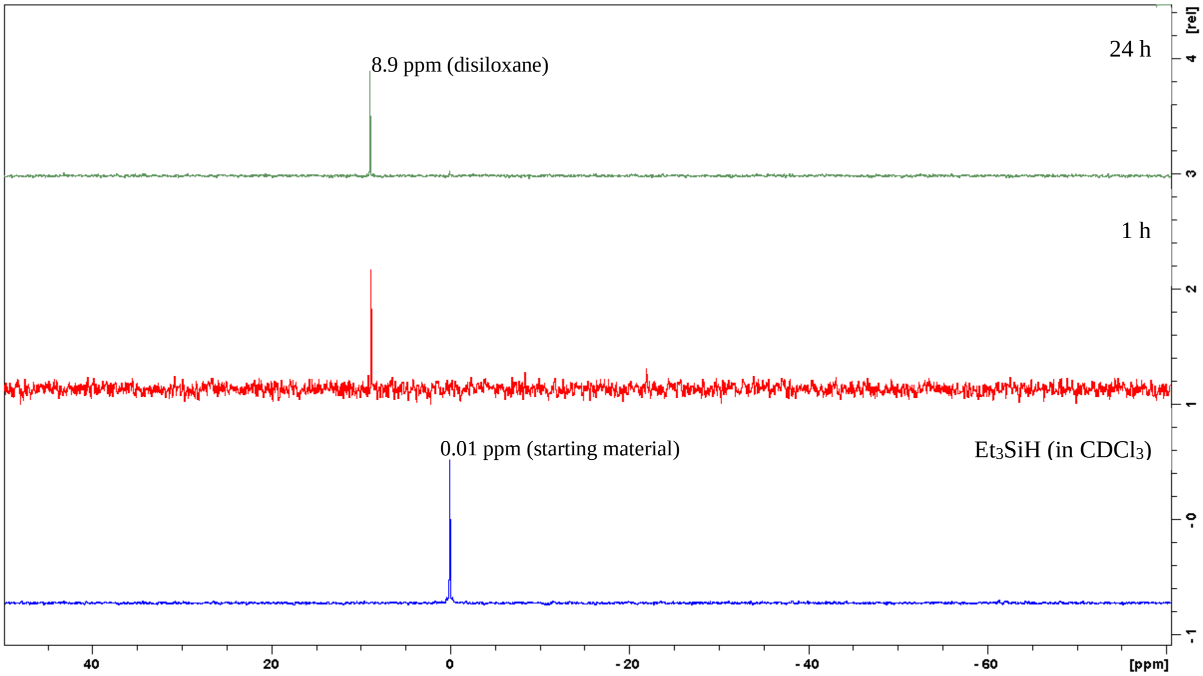

Supplementary Figure 9. $^{29}Si\{^1H\}$ NMR spectra of Et_3SiH (**1a**) (bottom, blue) after reacting with 1.0 mol% $(C_6F_5)_3B(OH_2)$ and 0.5 eq H_2O for 1 h (middle, red) and 24 h (top, green) in toluene-d_8

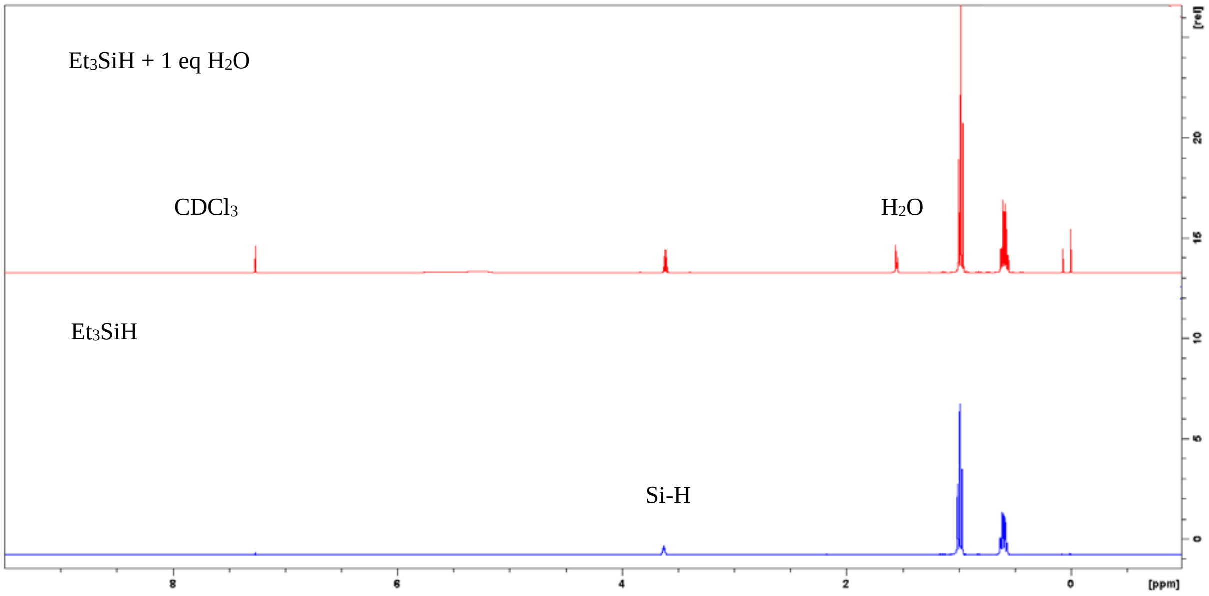

Supplementary Figure 10. ^1H NMR spectra of Et_3SiH (**1a**) after a reaction with 1 eq H_2O in $CDCl_3$

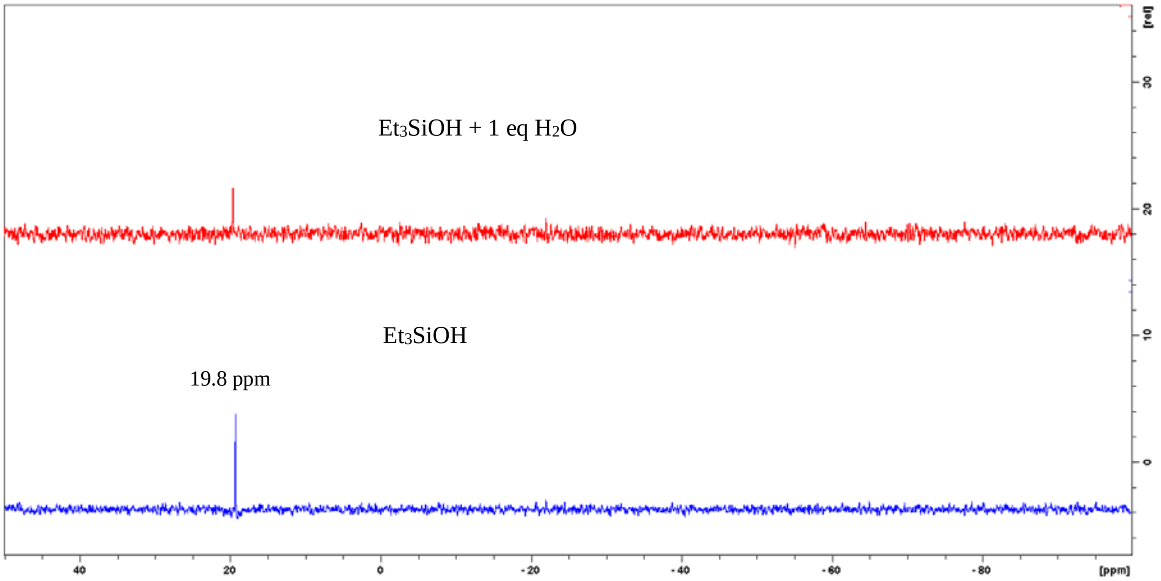

Supplementary Figure 11. $^{29}Si\{^1H\}$ NMR spectra of Et_3SiOH (**2a**) after a reaction with 1 eq H_2O in $CDCl_3$

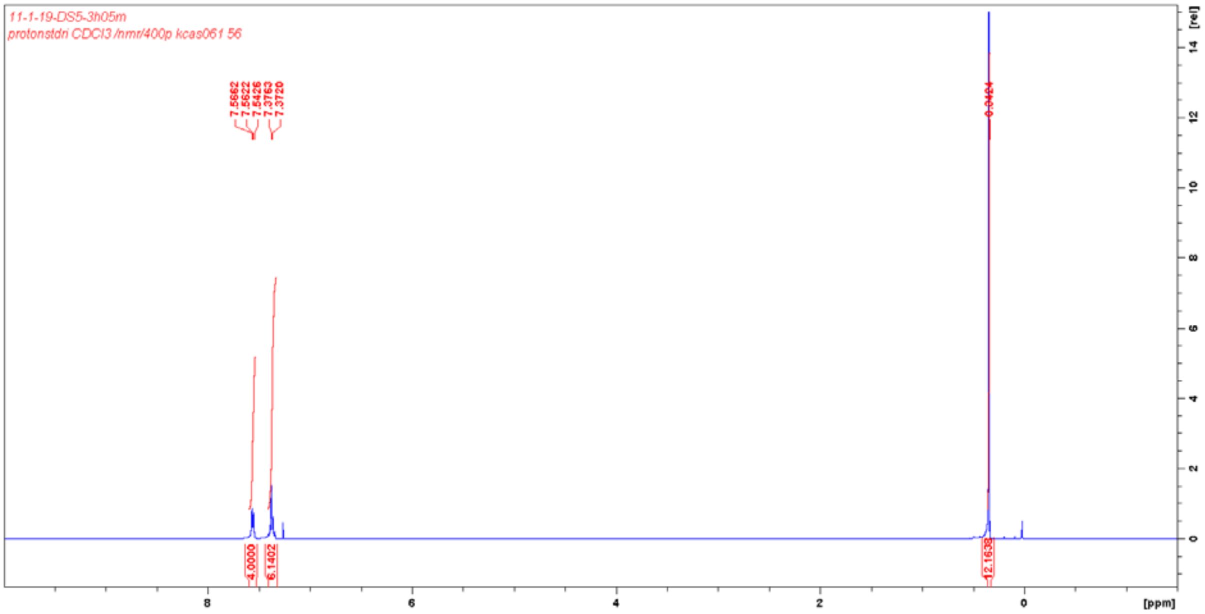

Supplementary Figure 12. ^1H NMR spectrum of $PhMe_2SiOSiMe_2Ph$ (**3b**) in $CDCl_3$

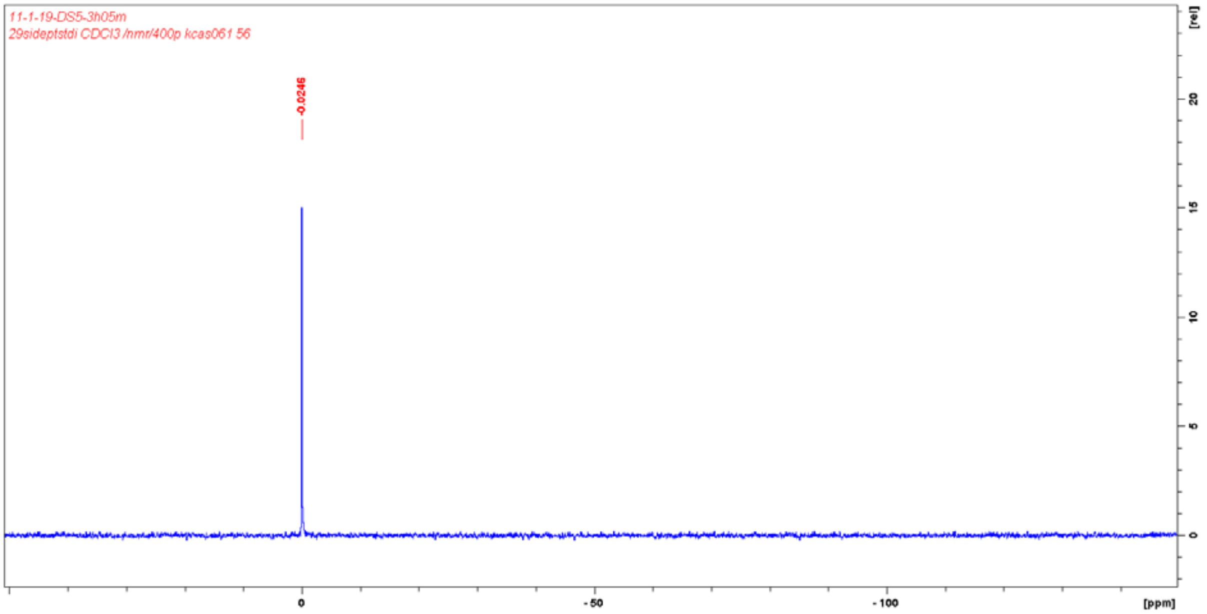

Supplementary Figure 13. $^{29}Si\{^{1}H\}$ NMR spectrum of $PhMe_2SiOSiMe_2Ph$ (**3b**) in $CDCl_3$

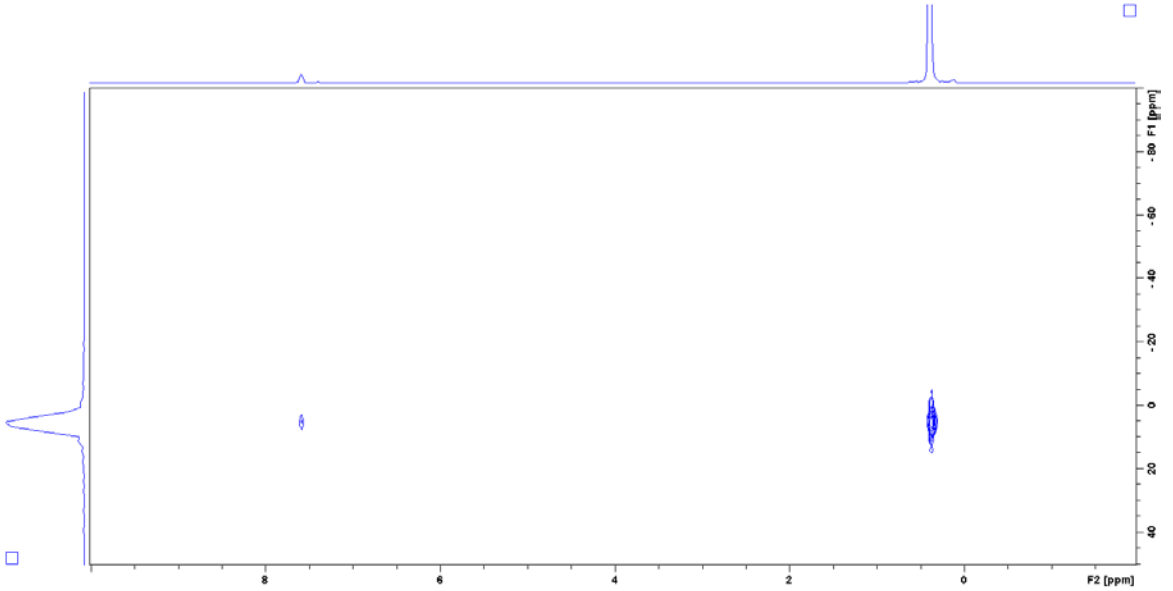

Supplementary Figure 14. $^{29}Si\{^{1}H\}$-HMBC NMR spectrum of $PhMe_2SiOSiMe_2Ph$ (**3b**) in $CDCl_3$

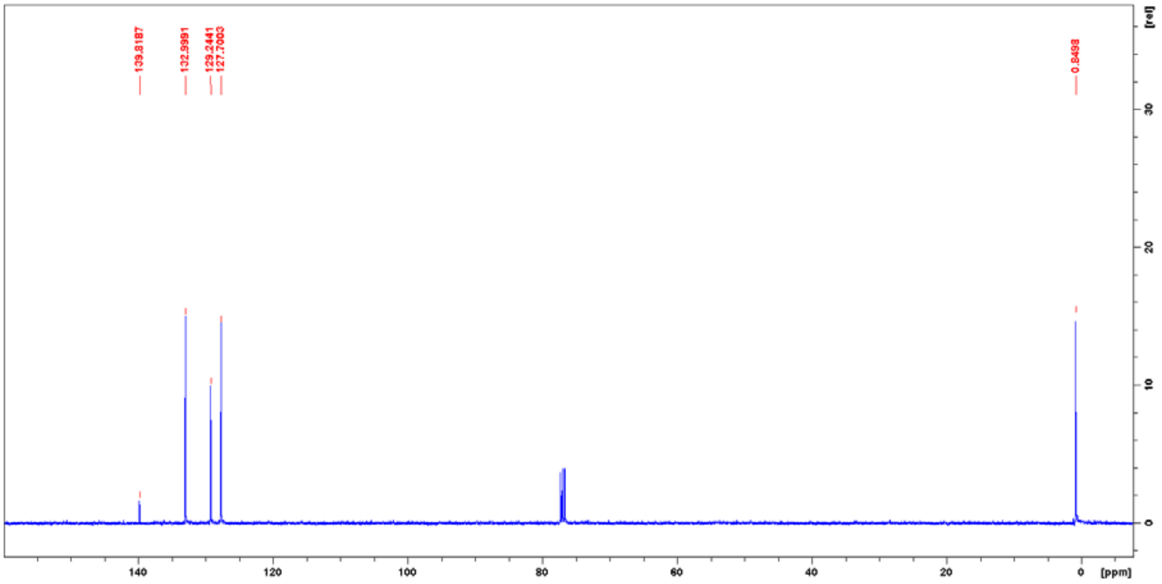

Supplementary Figure 15. $^{13}C\{^{1}H\}$ NMR spectrum of $PhMe_2SiOSiMe_2Ph$ (**3b**) in $CDCl_3$

Method	Pos_low_infusion.m	Operator	Admin	
Sample Name	DS5	Instrument / Ser#	micrOTOF-Q	228888.10191
Comment	Sample disovled in toluene<br>Sample diluted 5ul in 1ml MeOH			

Acquisition Parameter

Source Type	ESI	Ion Polarity	Positive	Set Nebulizer	0.4 Bar
Focus	Not active	Set Capillary	4500 V	Set Dry Heater	180 °C
Scan Begin	50 m/z	Set End Plate Offset	-500 V	Set Dry Gas	4.0 l/min
Scan End	1000 m/z	Set Collision Cell RF	100.0 Vpp	Set Divert Valve	Waste

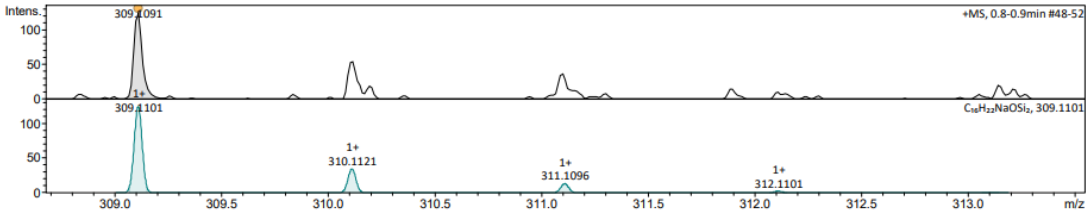

Meas. m/z	#	Ion Formula	m/z	err [ppm]	mSigma	# Sigma	Score	rdb	e⁻ Conf	N-Rule
309.1091	1	C16H22NaOSi2	309.1101	3.3	756.3	1	-1.#J	7.5	even	ok

Supplementary Figure 16. ESI-MS $PhMe_2SiOSiMe_2Ph$ (**3b**)

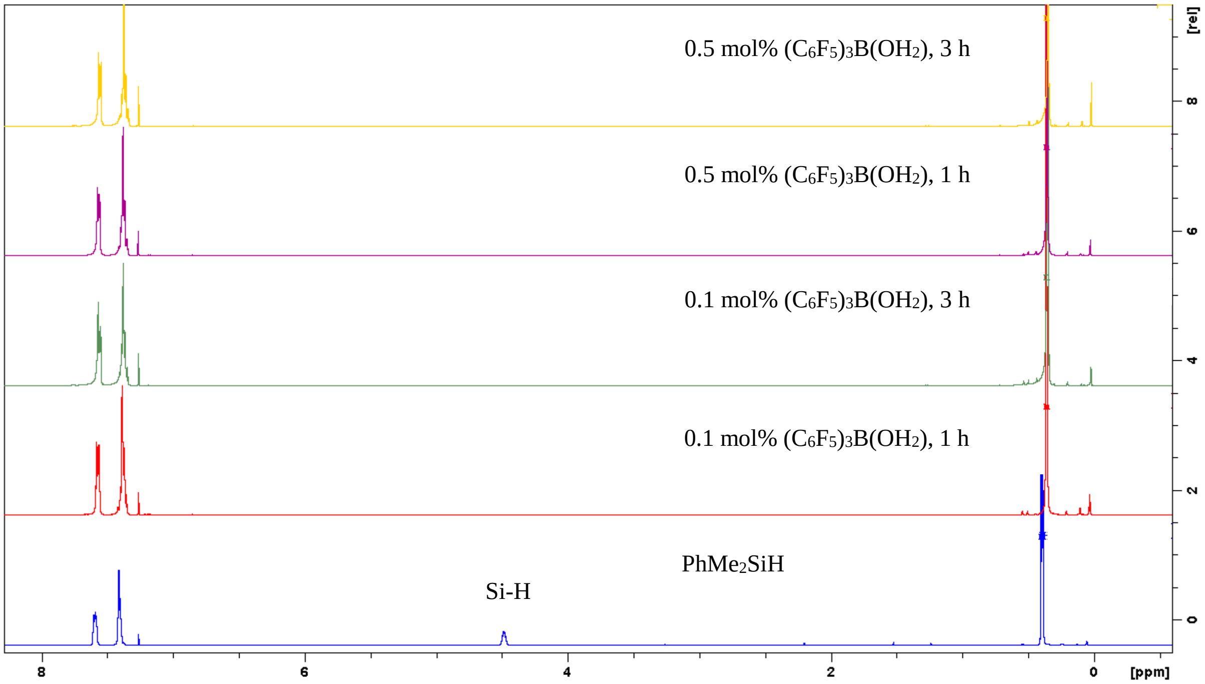

Supplementary Figure 17. ^{1}H NMR spectra of $PhMe_2SiH$ (**1b**) after a reaction with 0.1 and 0.5 mol% $(C_6F_5)_3B(OH_2)$ at 1 h and 3 h with 0.5 eq H_2O in $CDCl_3$

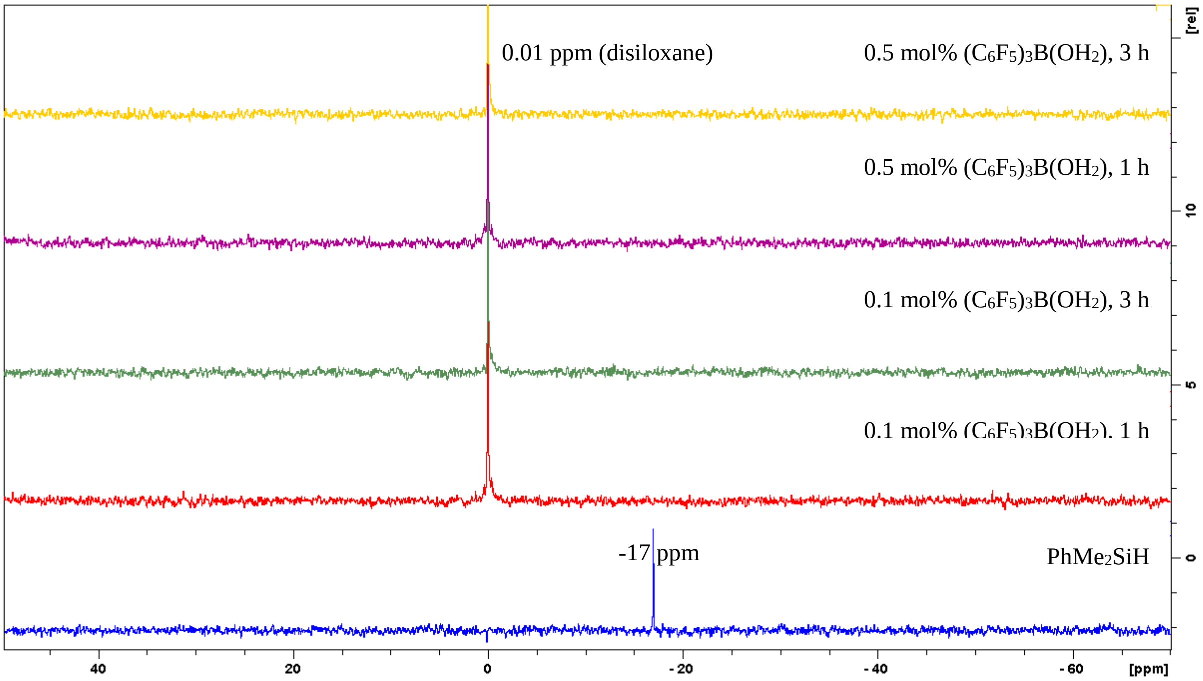

Supplementary Figure 18. $^{29}Si\{^{1}H\}$ NMR spectra of $PhMe_2SiH$ (**1b**) after a reaction with 0.1 and 0.5 mol% $(C_6F_5)_3B(OH_2)$ at 1 h and 3 h with 0.5 eq H_2O in $CDCl_3$

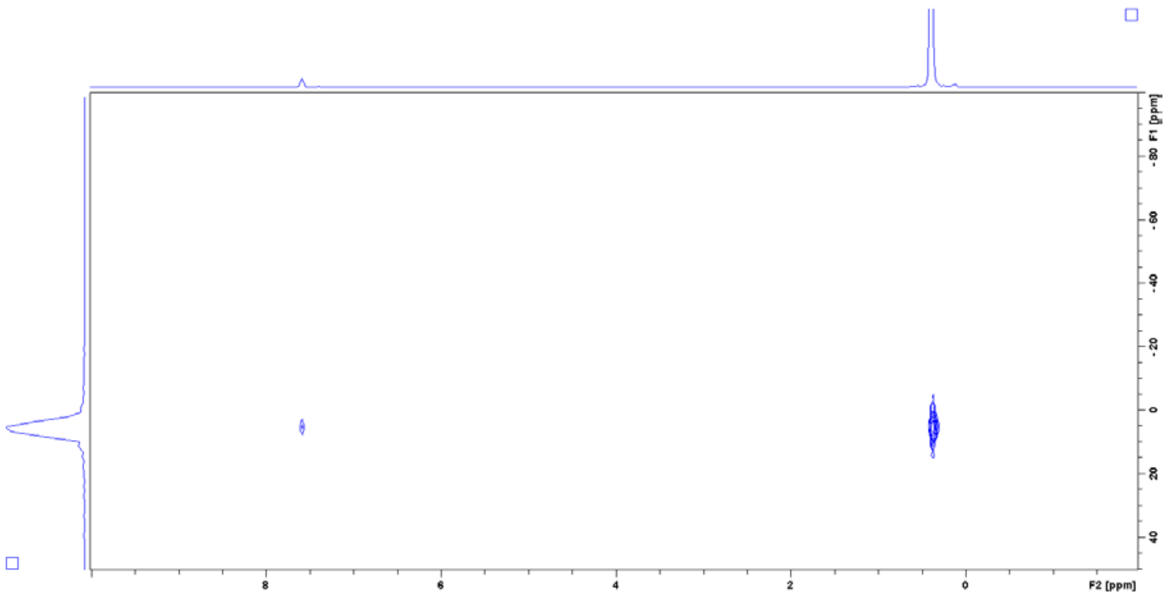

Supplementary Figure 19. $^{29}Si\{^1H\}$-HMBC NMR spectrum of $PhMe_2SiH$ (**1b**) after a reaction with 0.1 and 0.5 mol% $(C_6F_5)_3B(OH_2)$ with 0.5 eq H_2O in $CDCl_3$ ($PhMe_2SiOSiMe_2Ph$, **3b**)

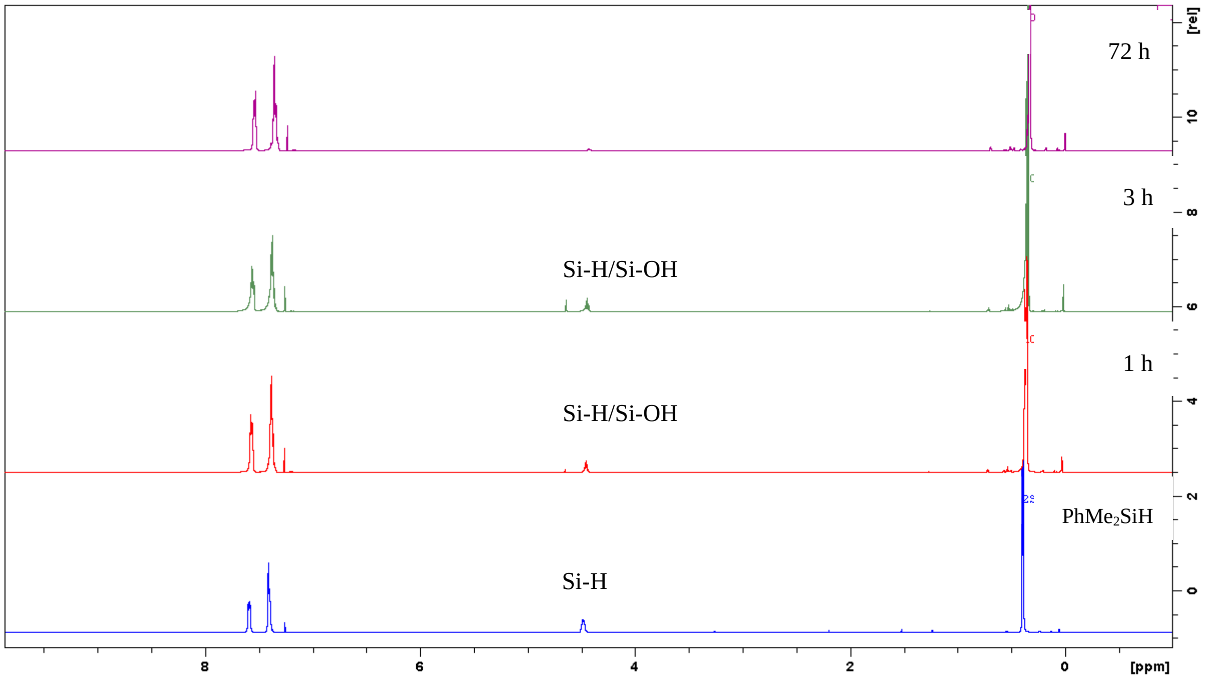

Supplementary Figure 20. ^1H NMR spectra of $PhMe_2SiH$ (**1b**) after a reaction with 1.0 mol% $(C_6F_5)_3B(OH_2)$ and 0.2 eq H_2O over time in $CDCl_3$

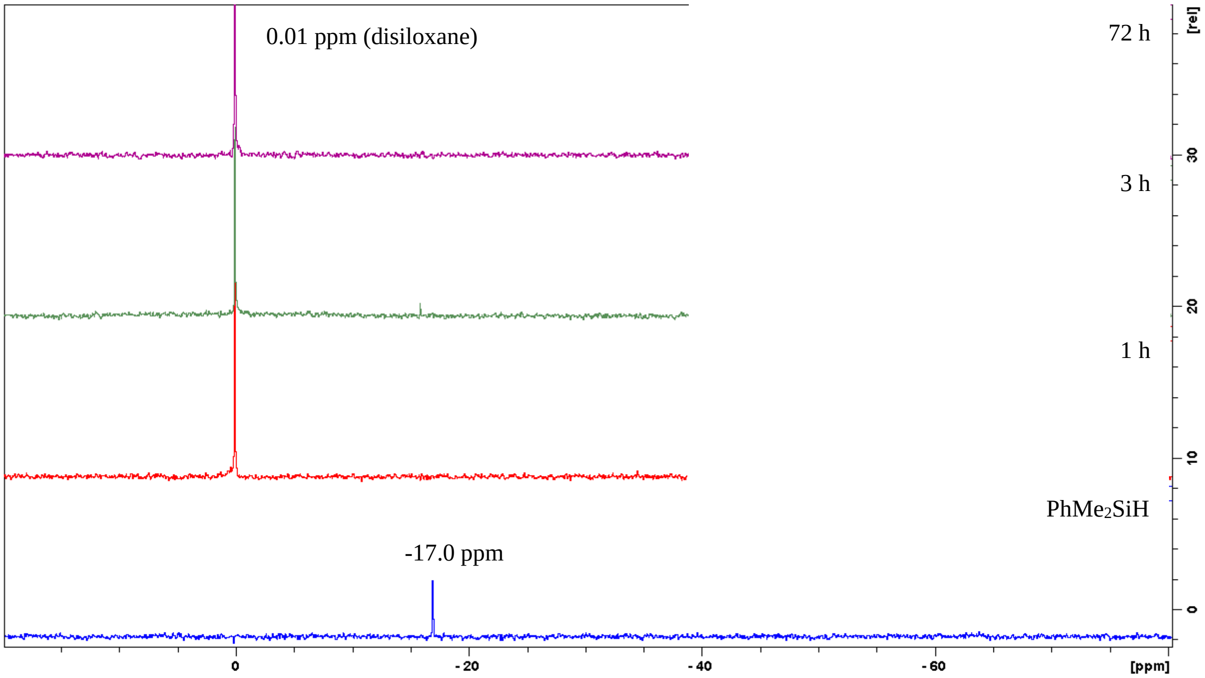

Supplementary Figure 21. $^{29}Si\{^1H\}$ NMR spectra of $PhMe_2SiH$ (**1b**) after a reaction with 1.0 mol% $(C_6F_5)_3B(OH_2)$ and 0.2 eq H_2O over time in $CDCl_3$ ($PhMe_2SiOSiMe_2Ph$, **3b**)

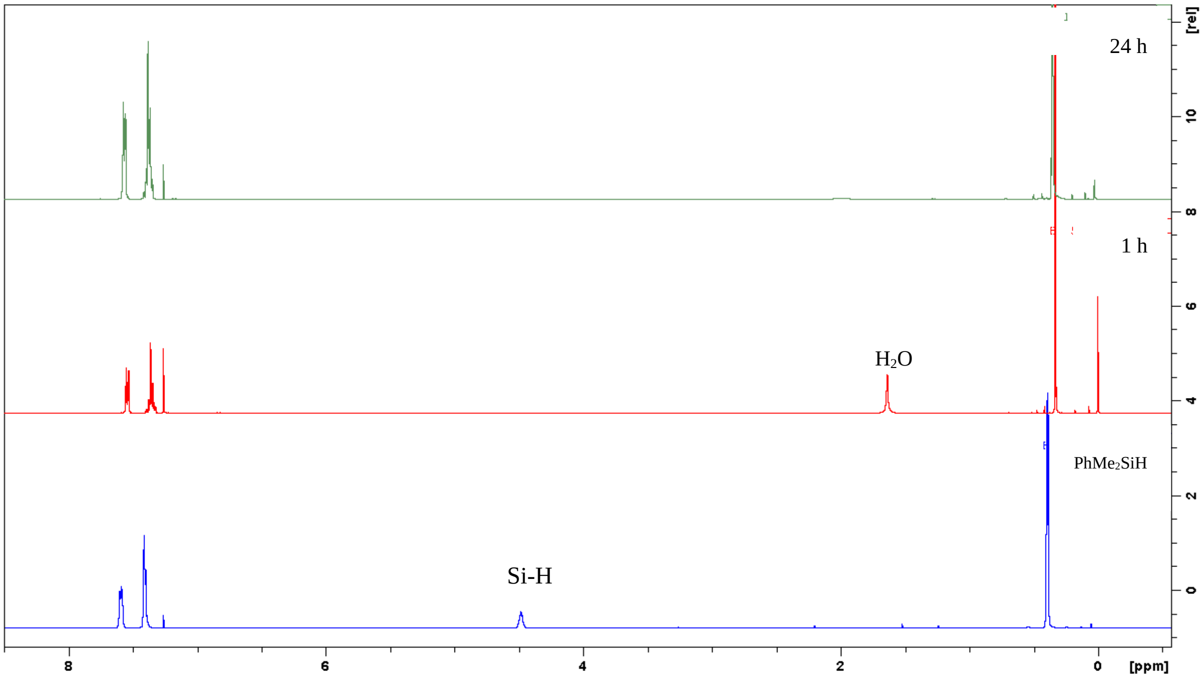

Supplementary Figure 22. ^1H NMR spectra of $PhMe_2SiH$ (**1b**) after a reaction with 1.0 mol% $(C_6F_5)_3B(OH_2)$ with excess H_2O over time in $CDCl_3$ ($PhMe_2SiOSiMe_2Ph$, **3b**)

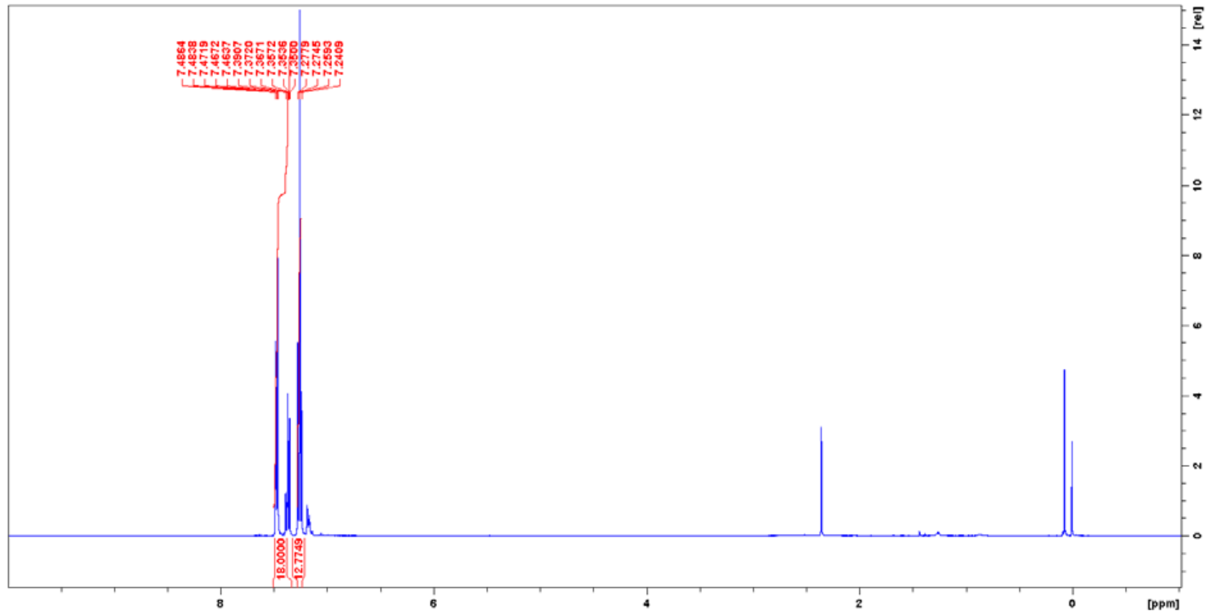

Supplementary Figure 23. ^{1}H NMR spectrum of $Ph_3SiOSiPh_3$ (**3c**) in toluene-d_8

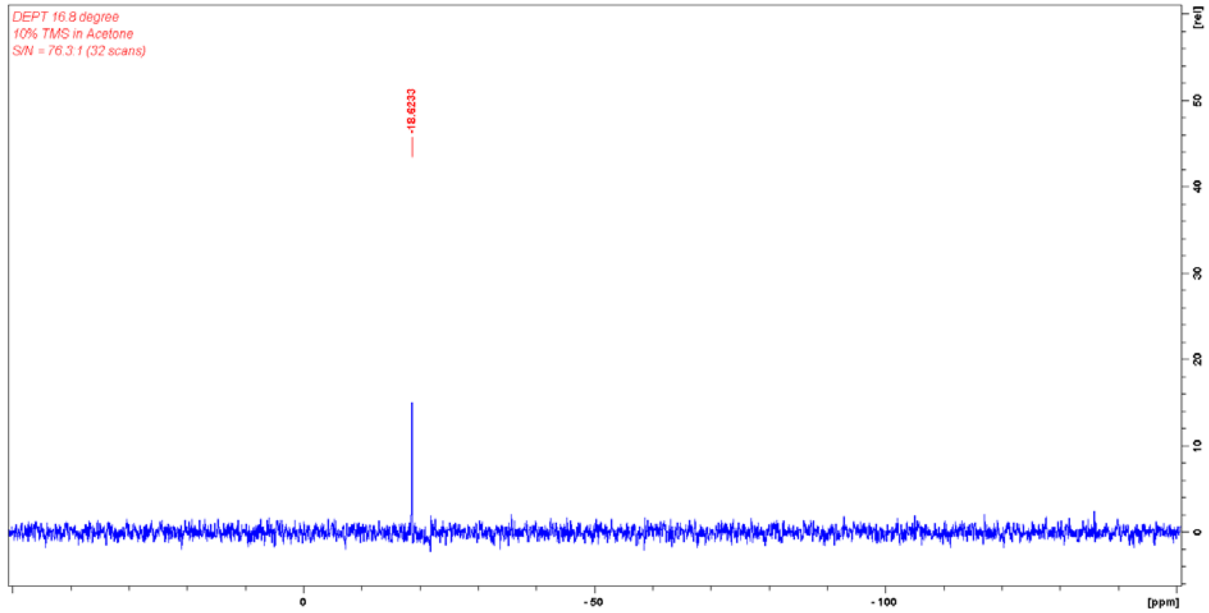

Supplementary Figure 24. $^{29}Si\{^{1}H\}$ NMR spectrum of $Ph_3SiOSiPh_3$ (**3c**) in toluene-d_8

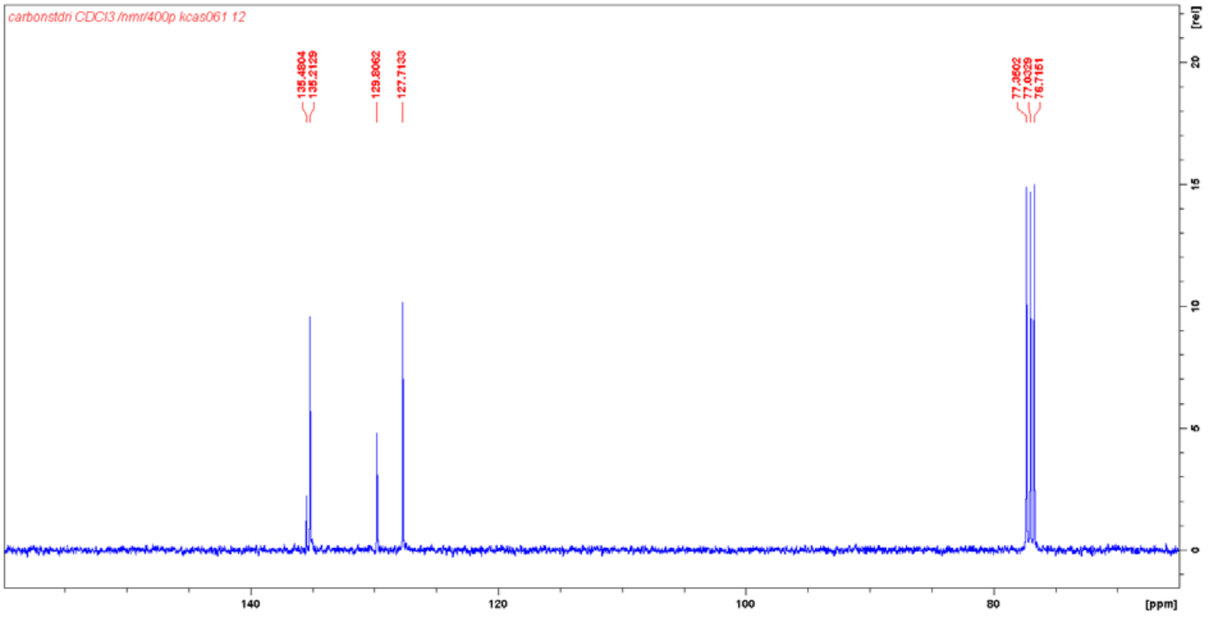

Supplementary Figure 25. $^{13}C\{^{1}H\}$ NMR spectrum of $Ph_3SiOSiPh_3$ (**3c**) in $CDCl_3$

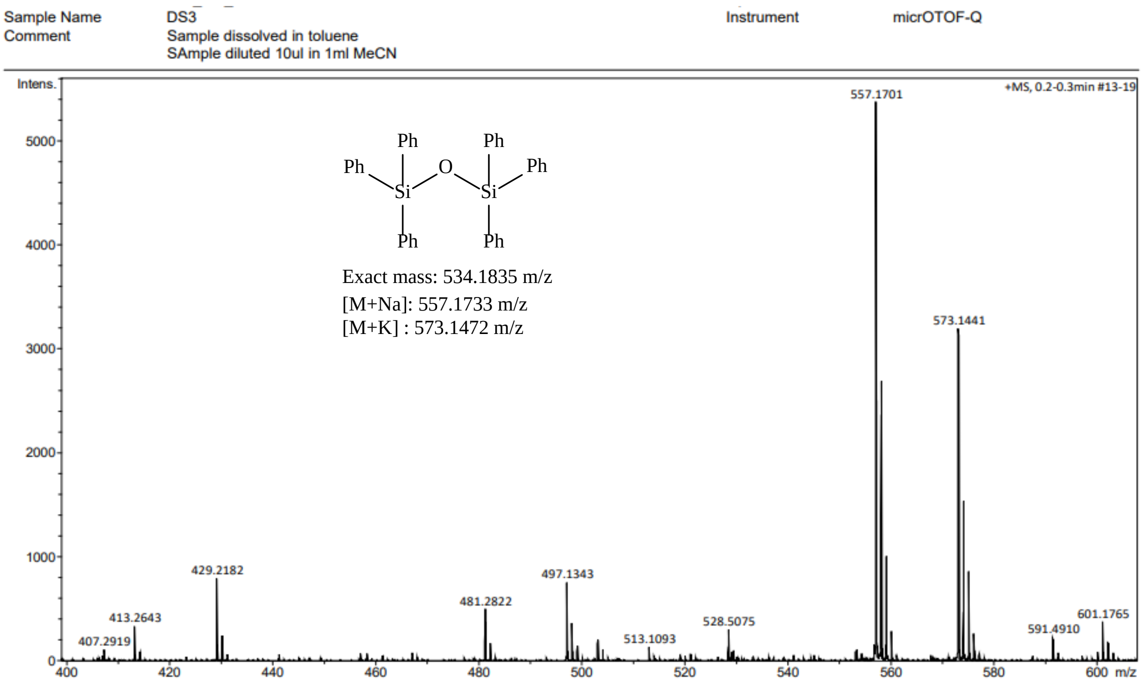

Supplementary Figure 26. ESI-MS of $Ph_3SiOSiPh_3$ (**3c**)

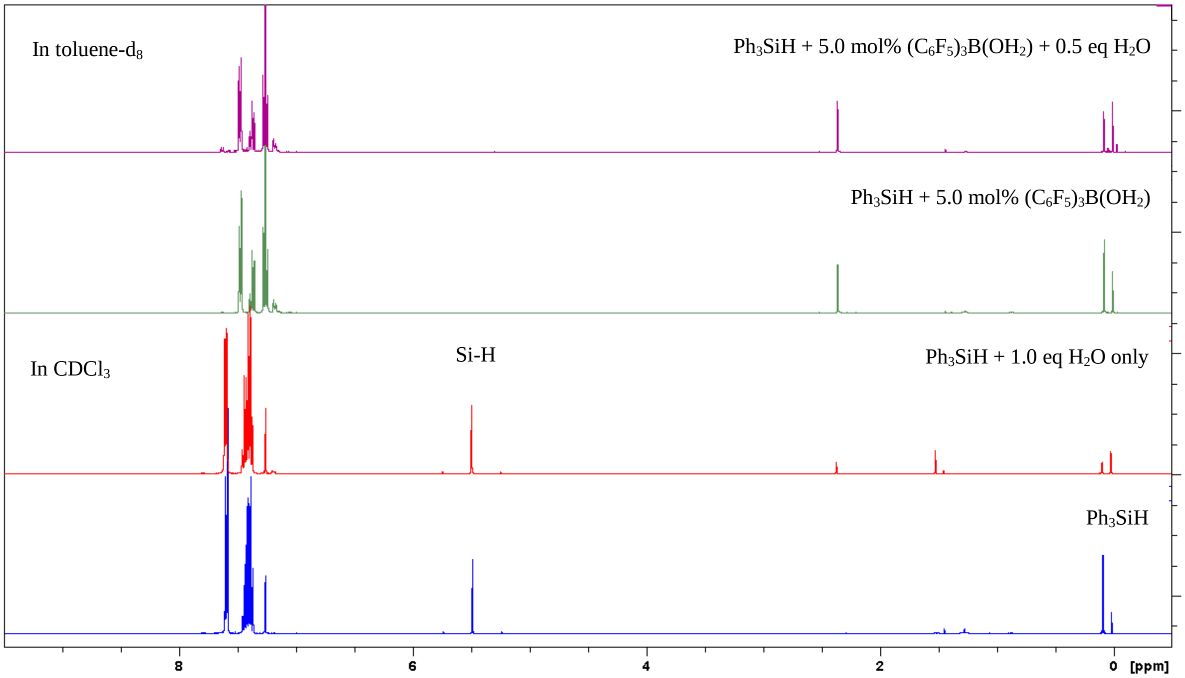

Supplementary Figure 27. ^{1}H NMR spectra of Ph_3SiH (**1c**) after a reaction with 5.0 mol% $(C_6F_5)_3B(OH_2)$ and 0.5 eq H_2O in toluene-d_8 to form $Ph_3SiOSiPh_3$ (**3c**)

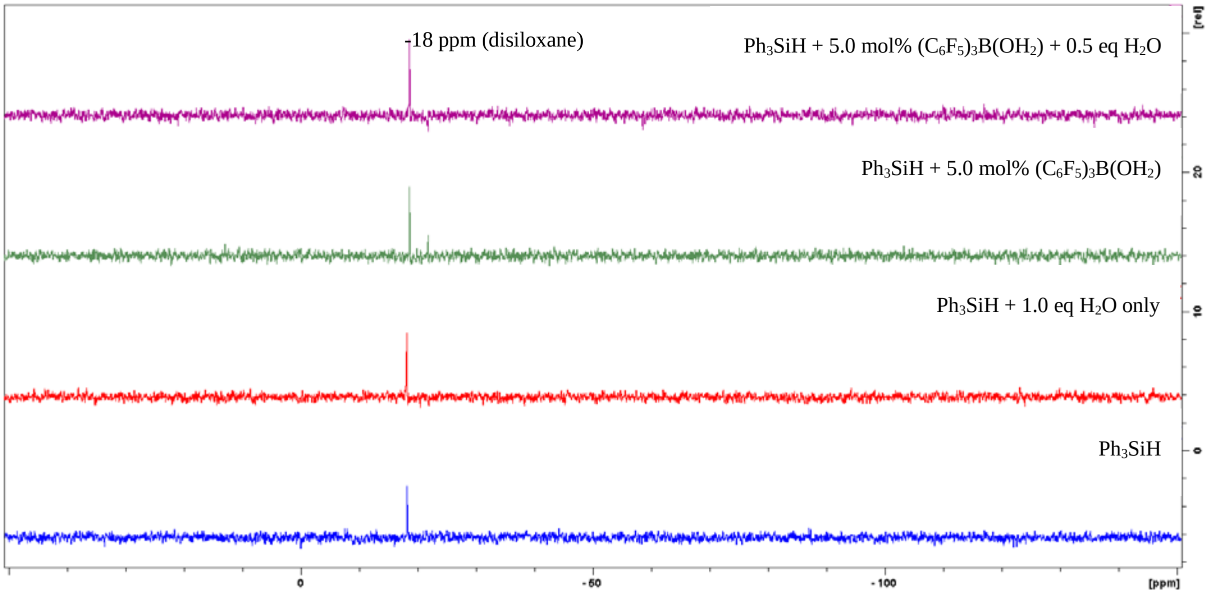

Supplementary Figure 28. ^{29}Si{^{1}H} NMR spectra of Ph_3SiH (**1c**) after a reaction with 5.0 mol% $(C_6F_5)_3B(OH_2)$ and 0.5 eq H_2O in toluene-d_8 to form $Ph_3SiOSiPh_3$ (**3c**)

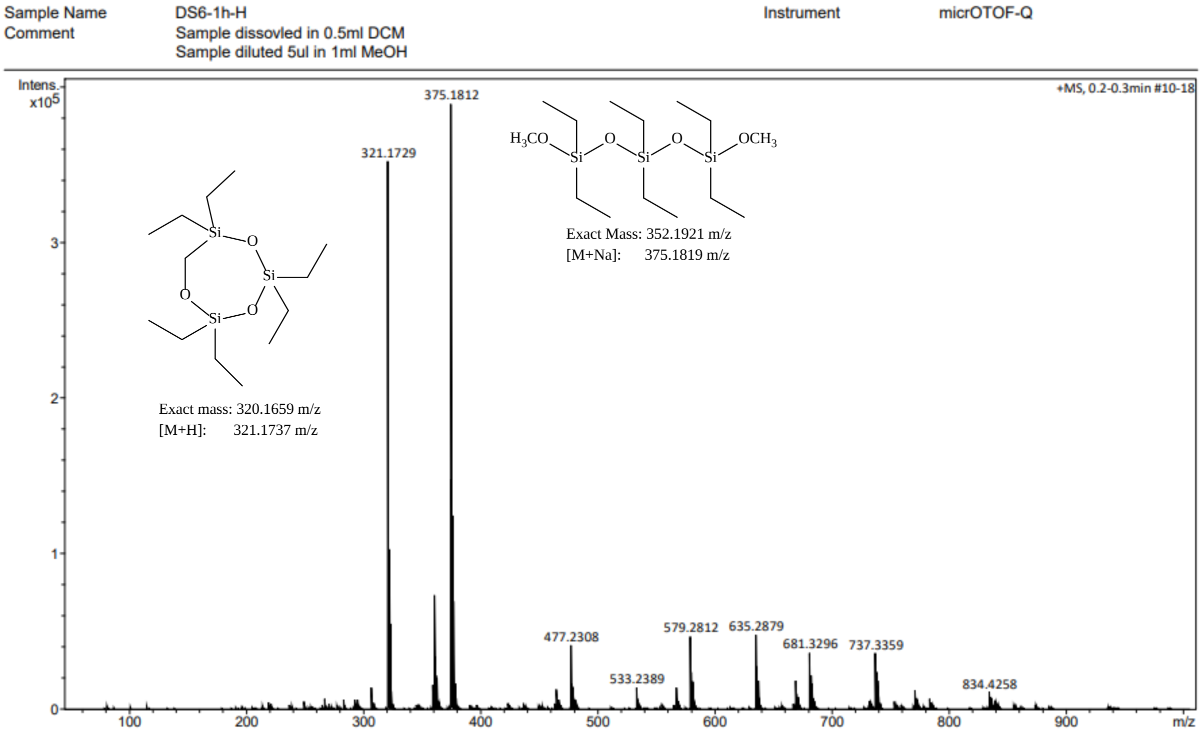

Supplementary Figure 29. ESI-MS of Et_2SiH_2 (**1d**) after a reaction with 2.0 mol% $(C_6F_5)_3B(OH_2)$ and 0.5 eq H_2O to form **3d** and **cyclo-3d**

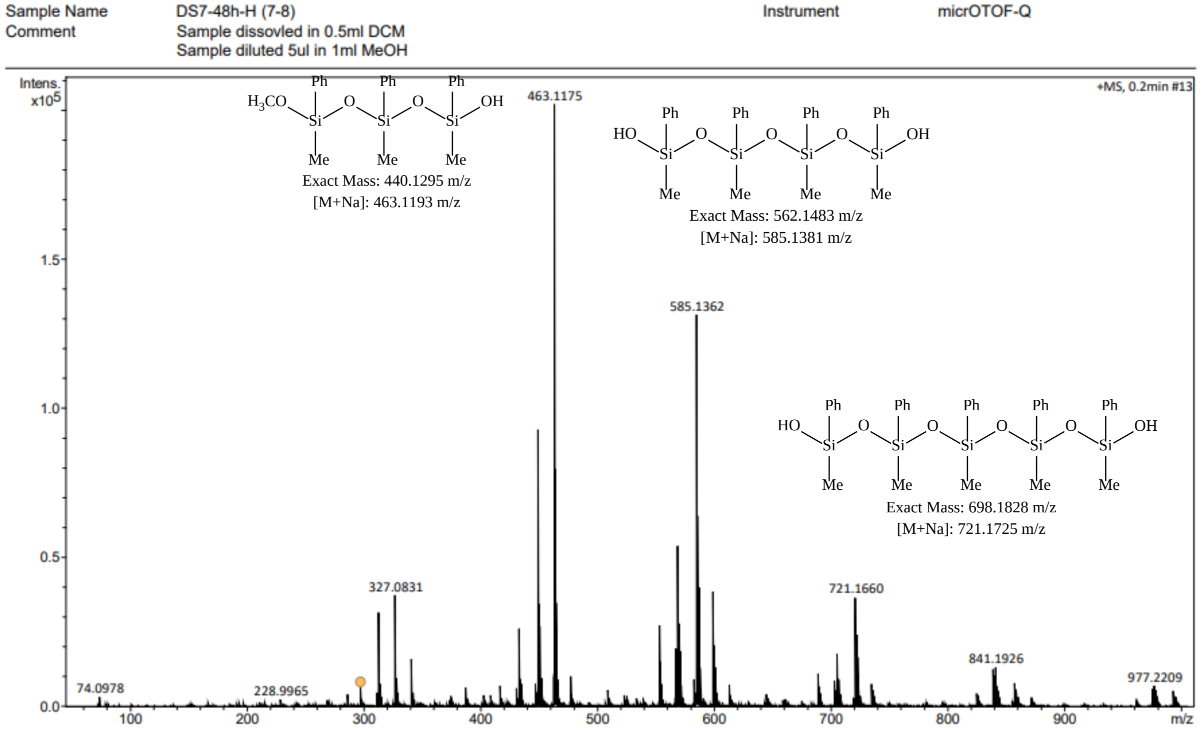

Supplementary Figure 30. ESI-MS of $PhMeSiH_2$ (**1e**) after a reaction with 2.0 mol% $(C_6F_5)_3B(OH_2)$ and 0.5 eq H_2O to form **3e**, **4e**, and **5e**.

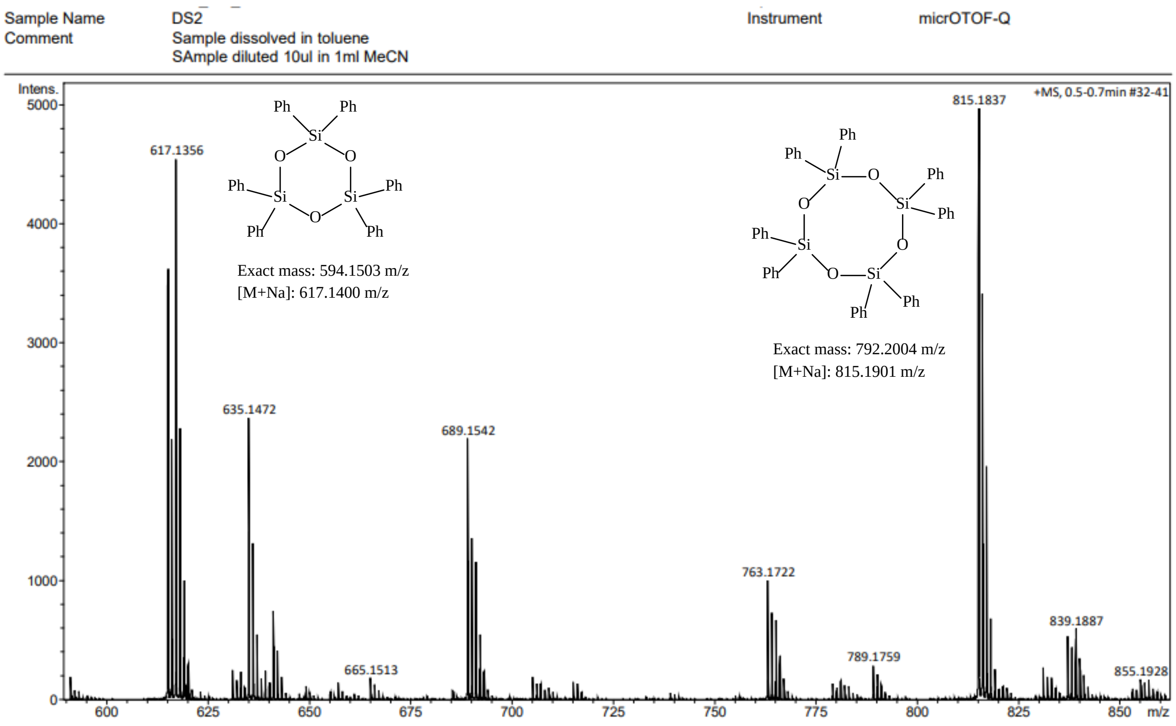

Supplementary Figure 31. ESI-MS of Ph_2SiH_2 (**1f**) after a reaction with 5.0 mol% $(C_6F_5)_3B(OH_2)$ and 0.5 eq H_2O to form **cyclo-3f** and **cyclo-4f**.

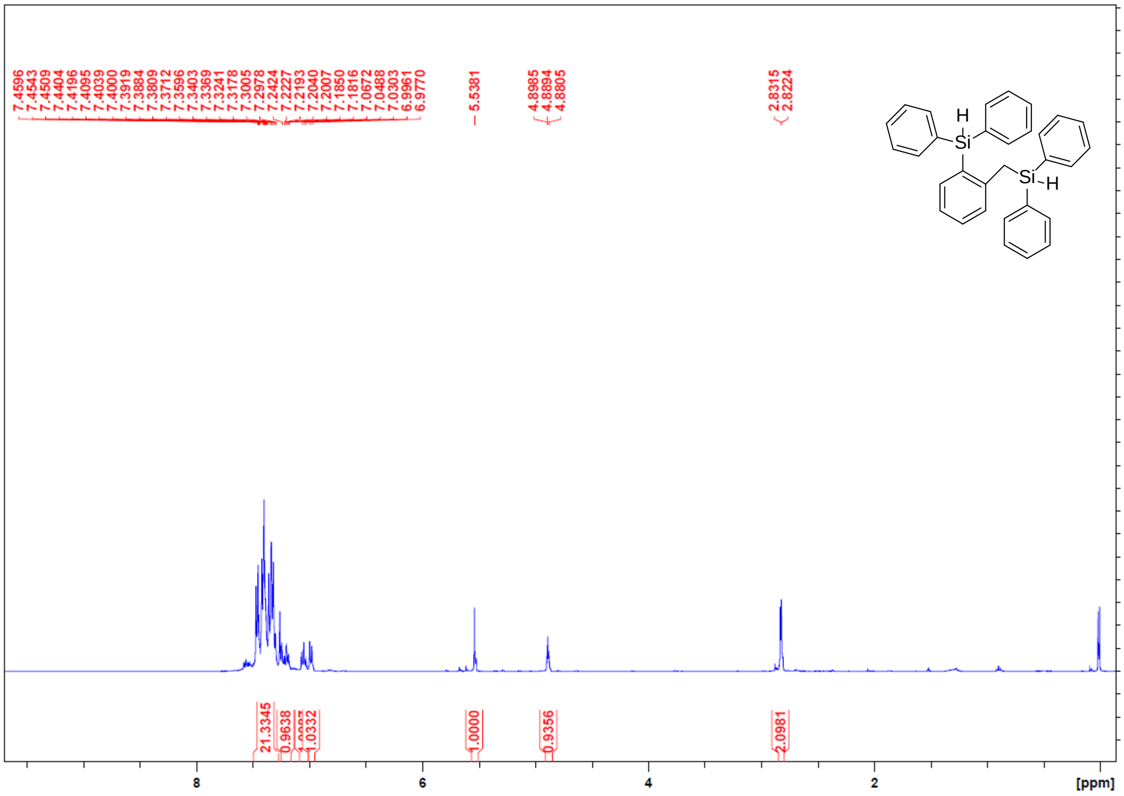

Supplementary Figure 32. ^{1}H NMR spectrum of **7a** in $CDCl_3$.

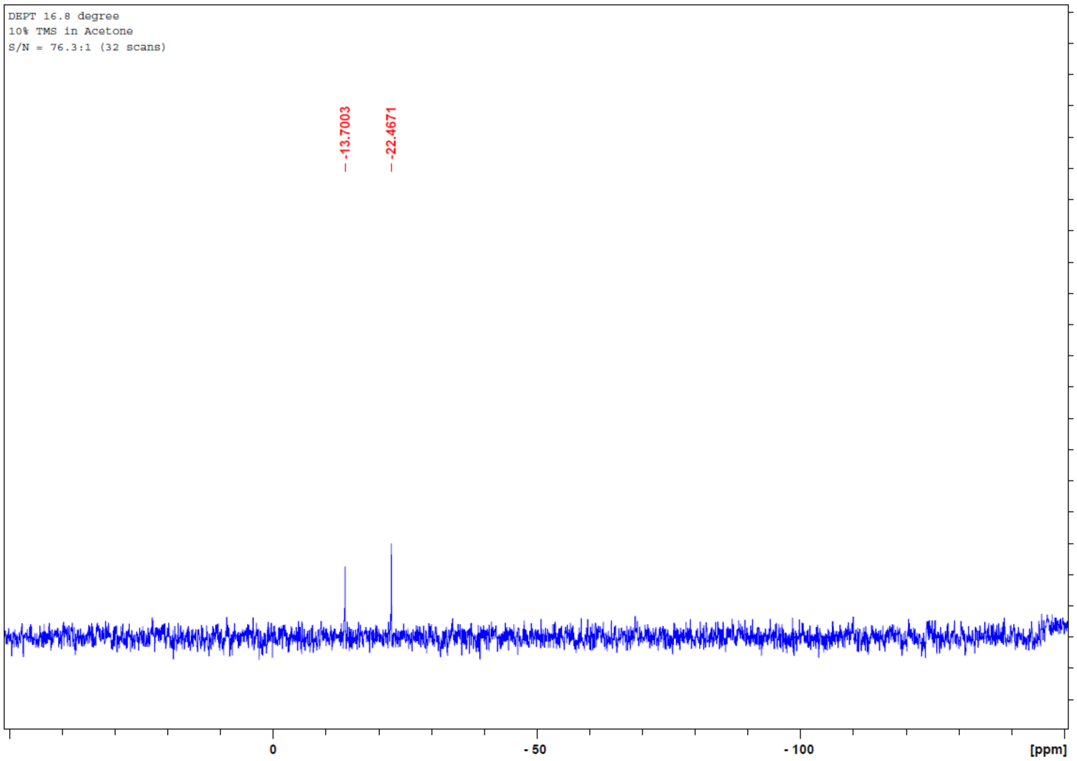

Supplementary Figure 33. ^{29}Si NMR spectrum of **7a** in $CDCl_3$.

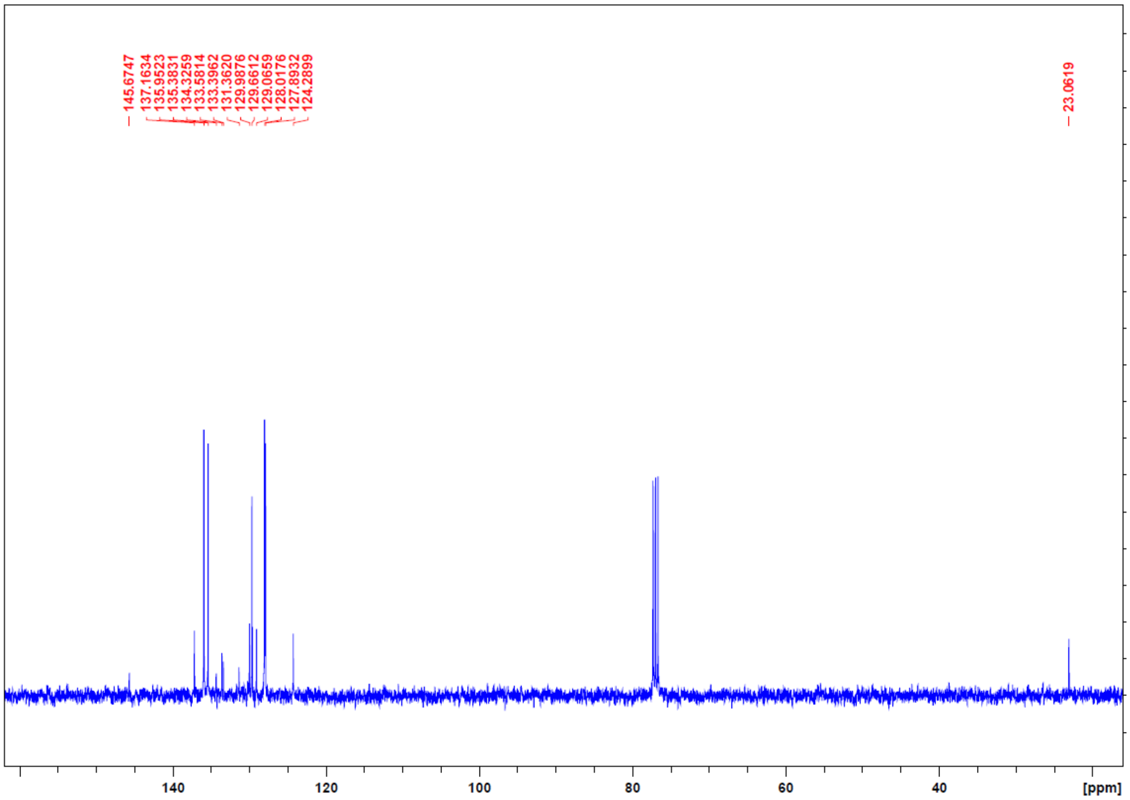

Supplementary Figure 34. ^{13}C NMR spectrum of **7a** in $CDCl_3$.

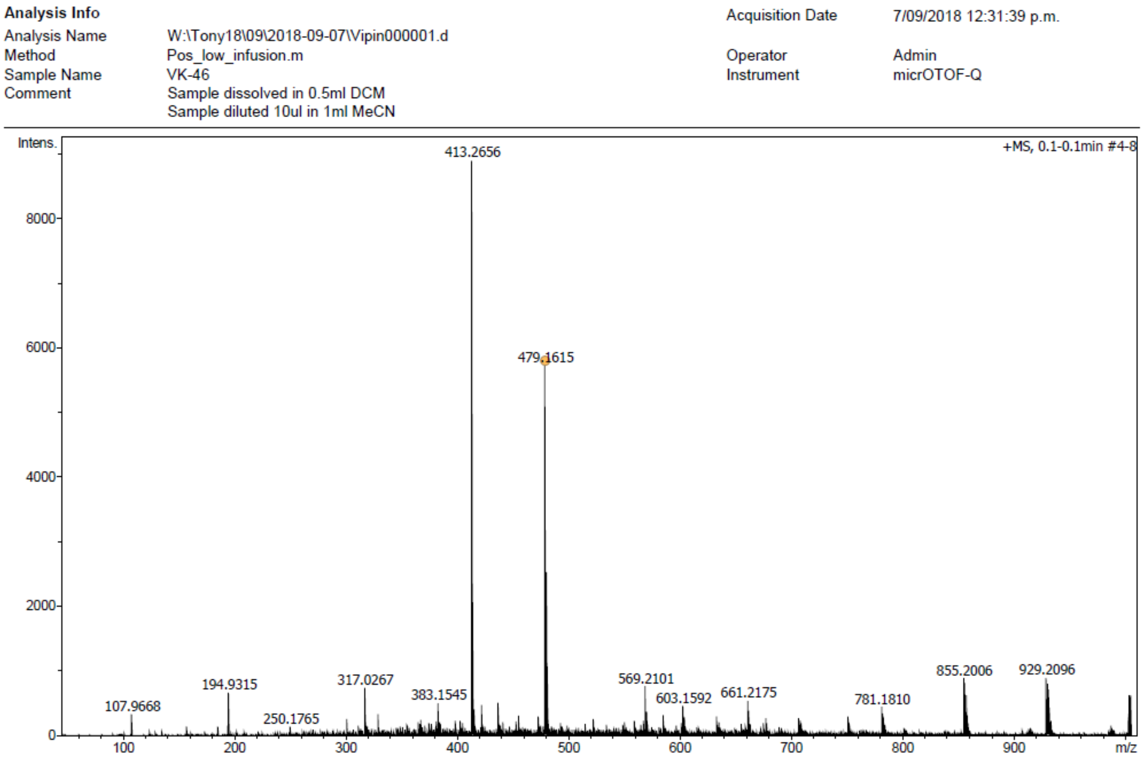

Supplementary Figure 35. ESI-MS of **7a**.

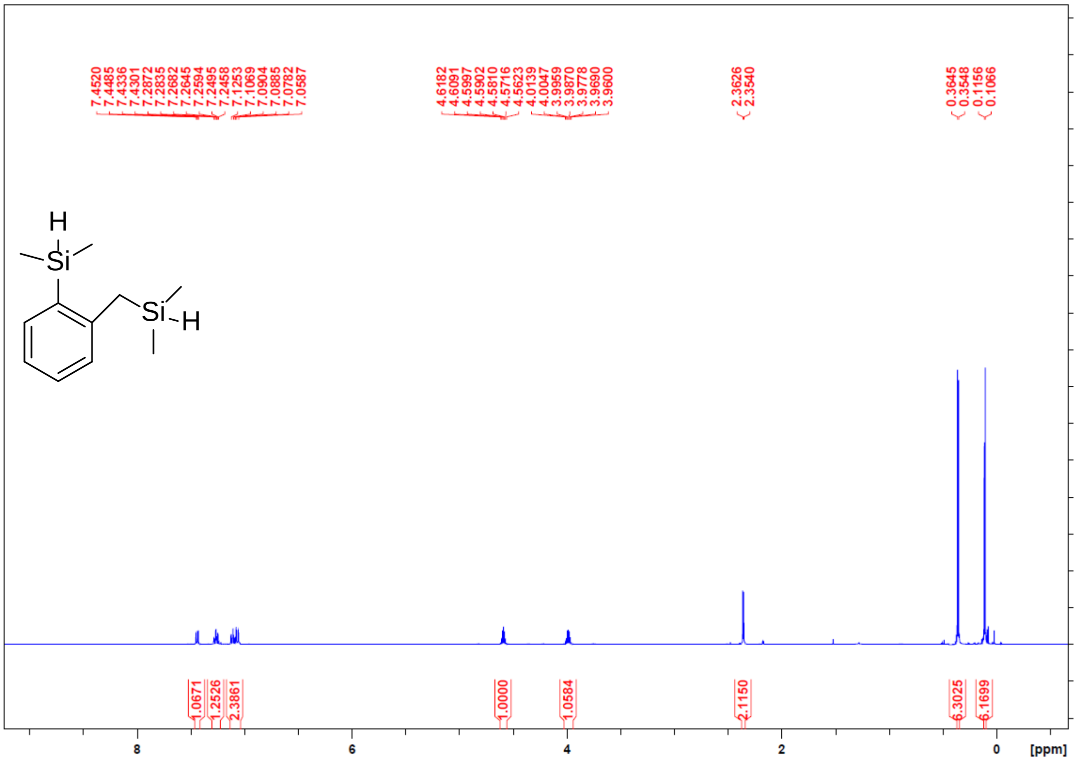

Supplementary Figure 36. ^{1}H NMR spectrum of **7b** in $CDCl_3$.

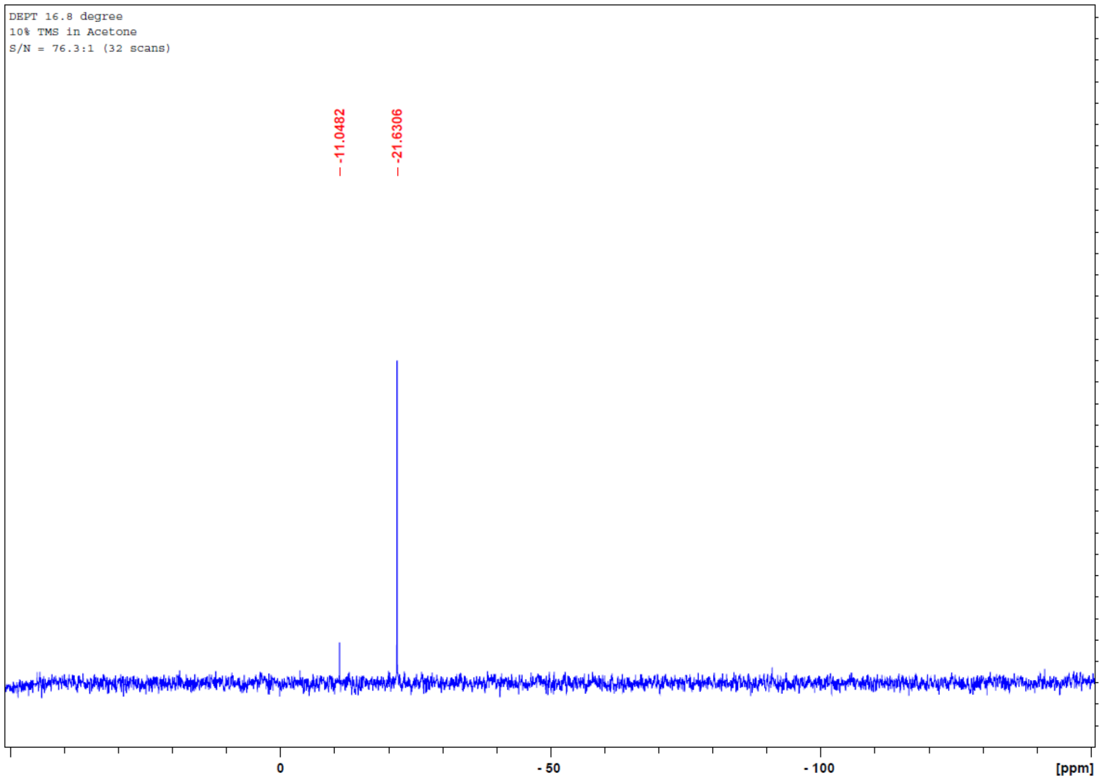

Supplementary Figure 37. ^{29}Si NMR spectrum of **7b** in $CDCl_3$.

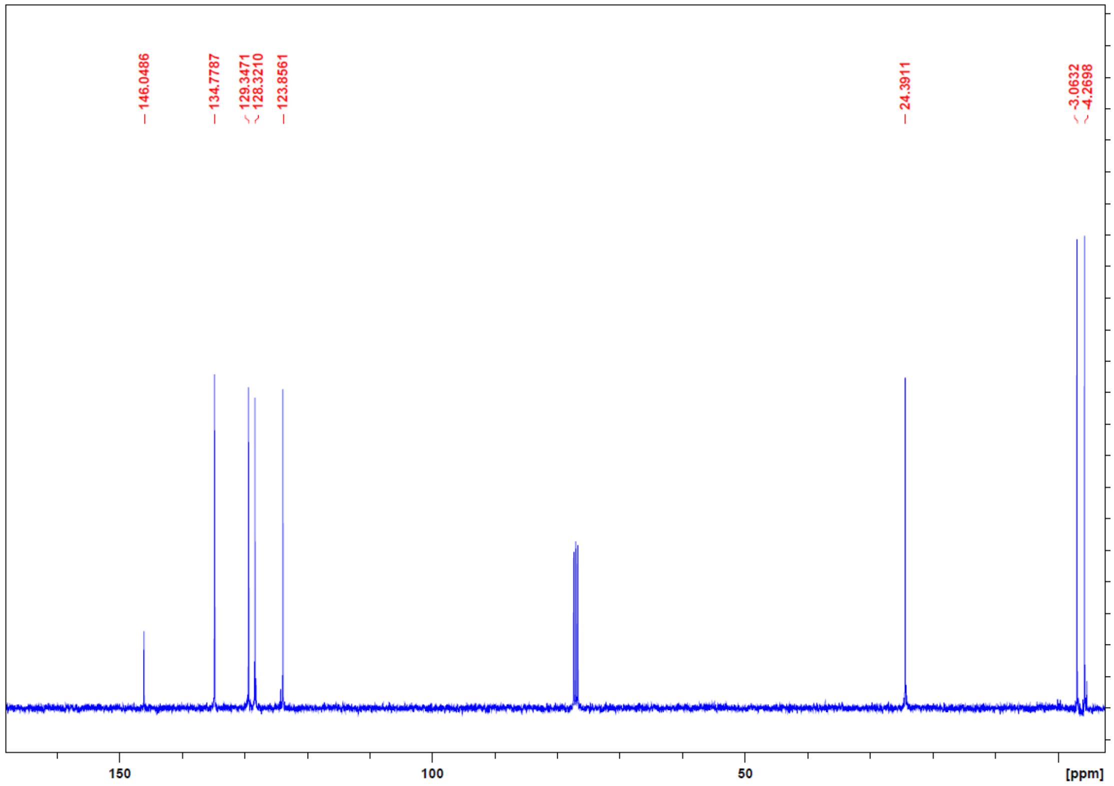

Supplementary Figure 38. ^{13}C NMR spectrum of **7b** in $CDCl_3$.

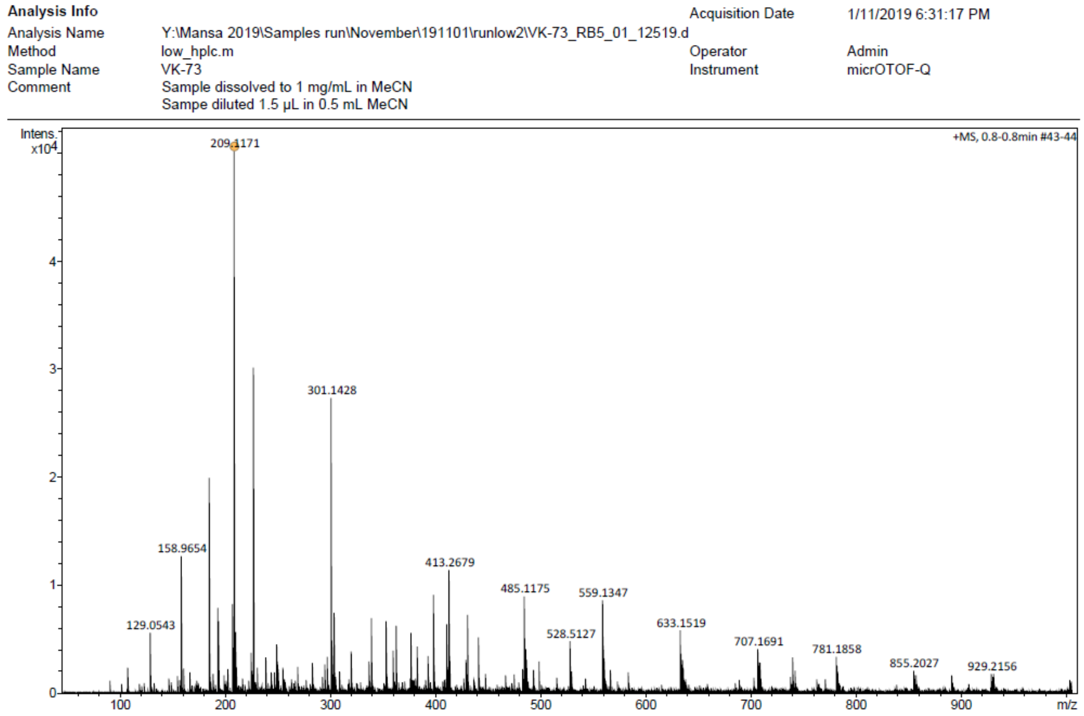

Supplementary Figure 39. ESI-MS of **7b**.

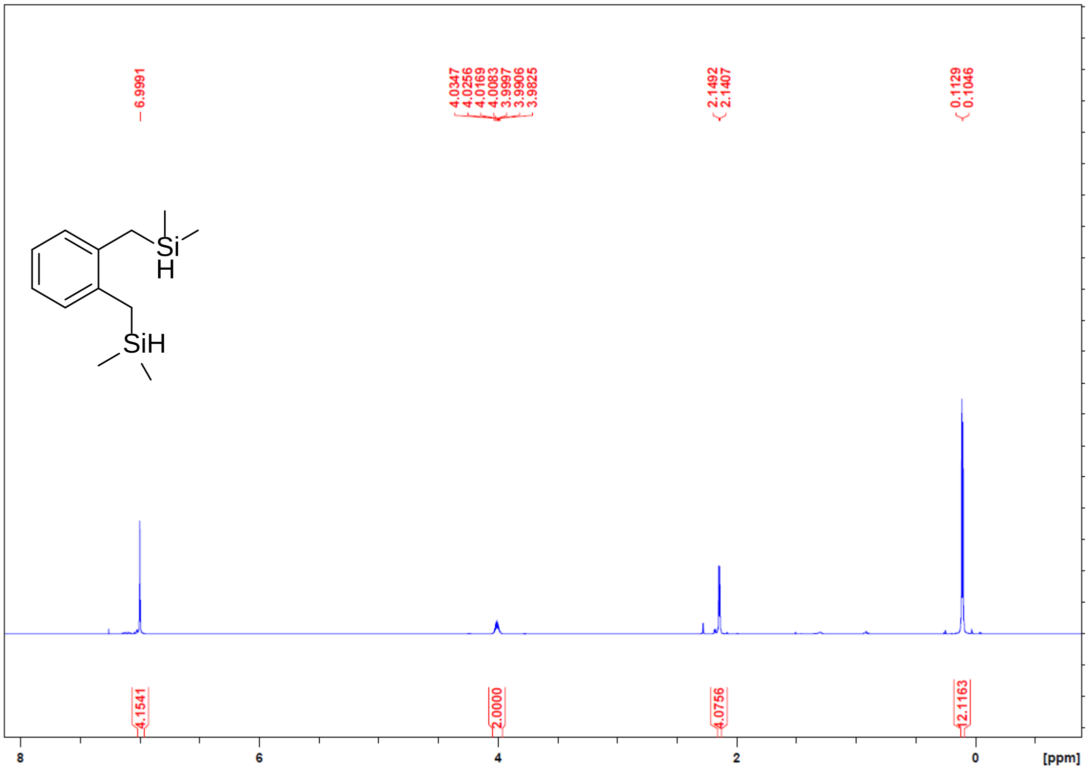

Supplementary Figure 40. ^{1}H NMR spectrum of **7c** in $CDCl_3$.

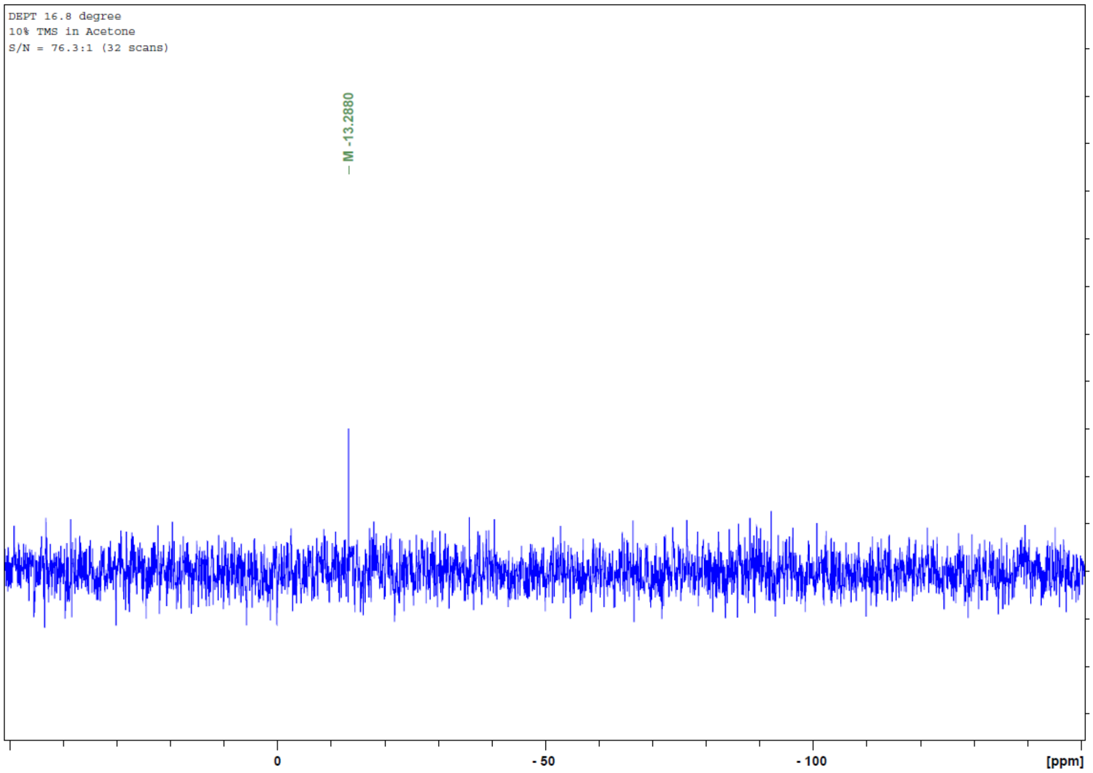

Supplementary Figure 41. ^{29}Si NMR spectrum of **7c** in $CDCl_3$.

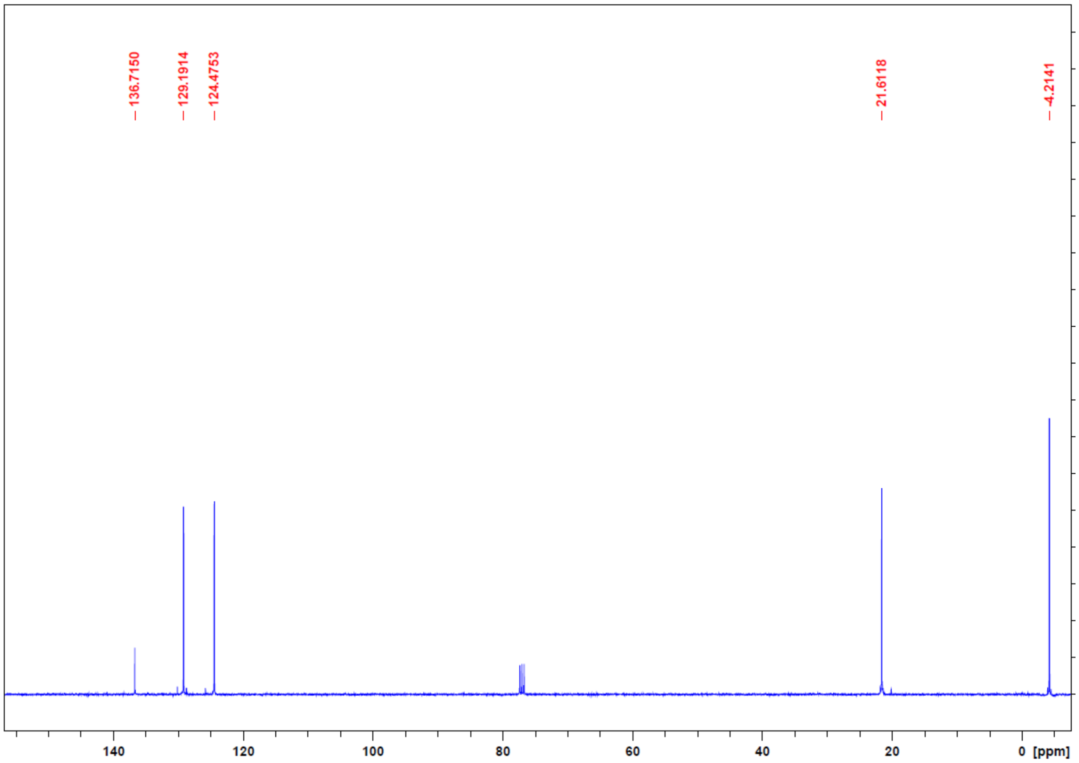

Supplementary Figure 42. ^{13}C NMR spectrum of **7c** in $CDCl_3$.

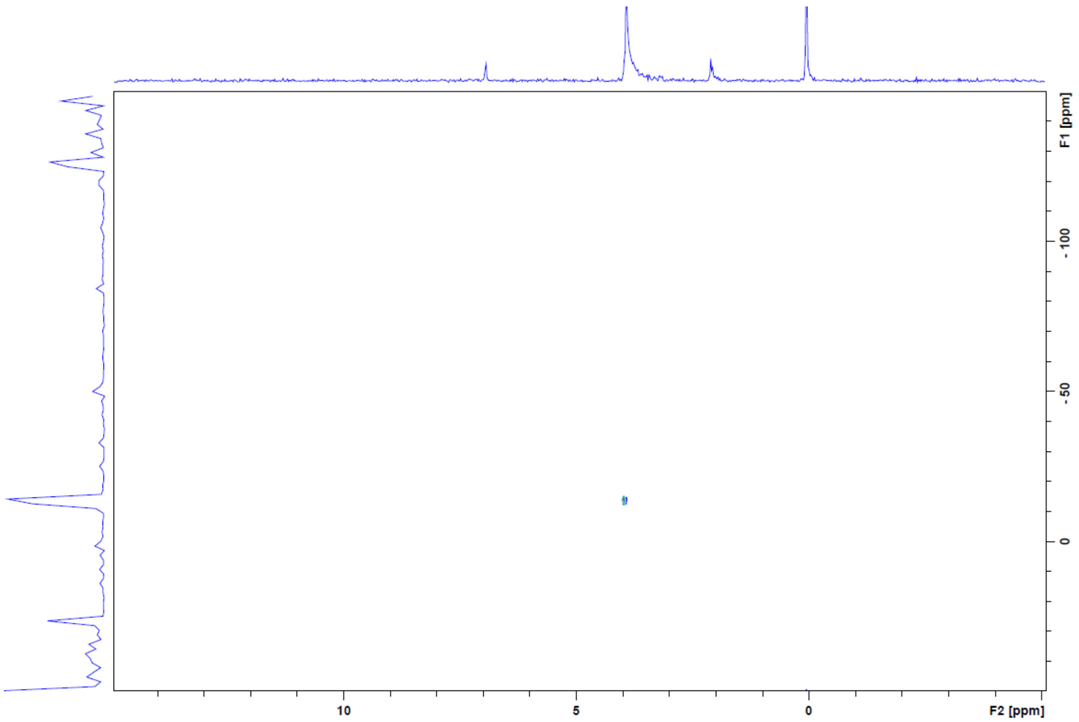

Supplementary Figure 43. $^{29}Si\{^{1}H\}$-HSQC NMR spectrum of **7c** in $CDCl_3$.

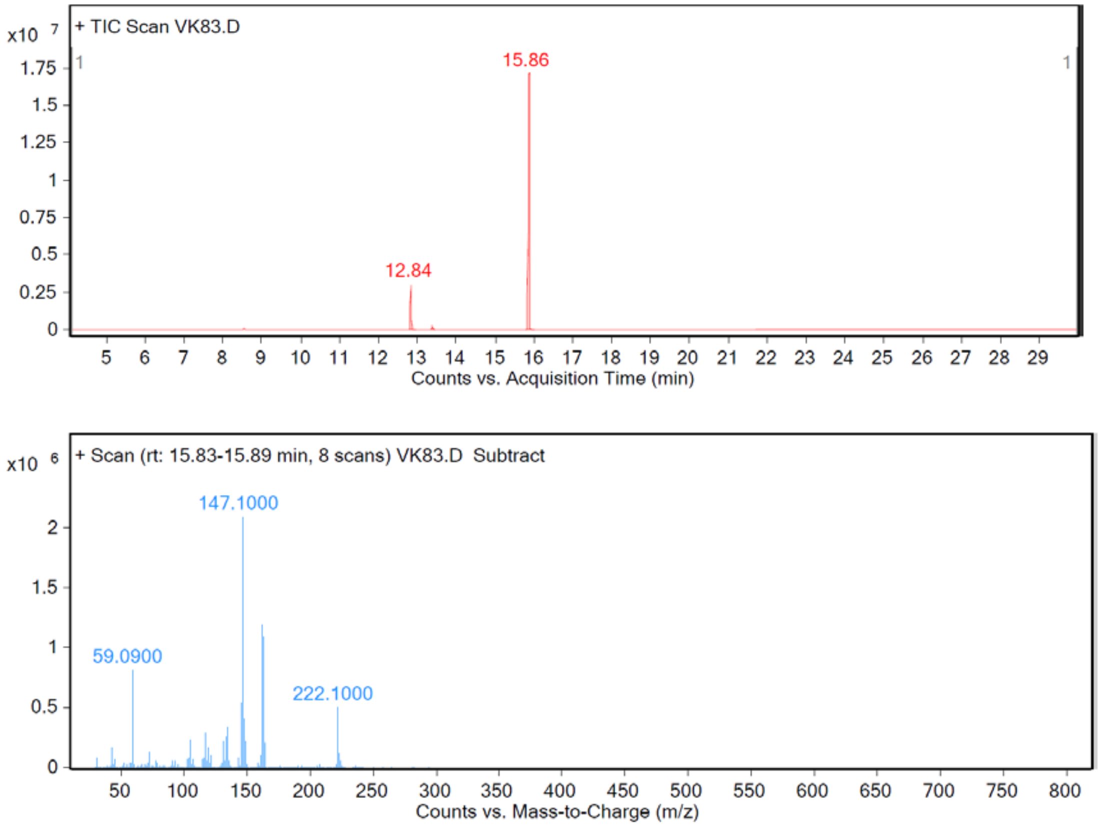

Supplementary Figure 44. GC-MS of **7c** in dichloromethane.

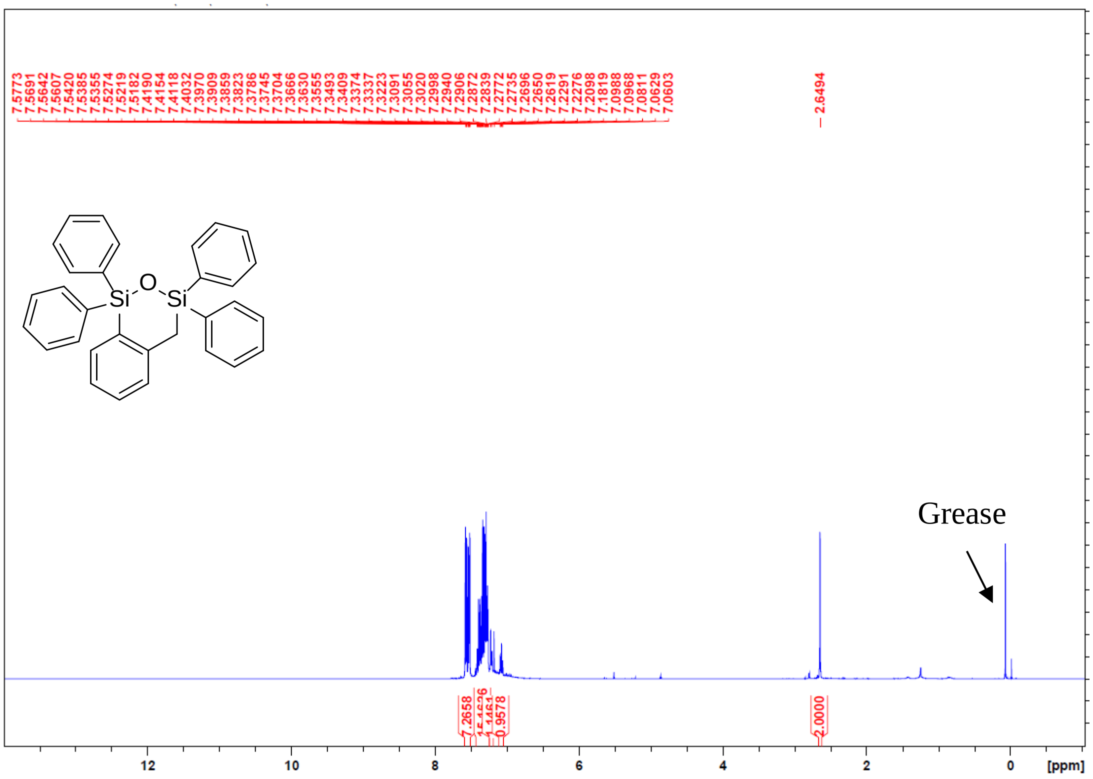

Supplementary Figure 45. ^{1}H NMR spectrum of **8a** in $CDCl_3$.

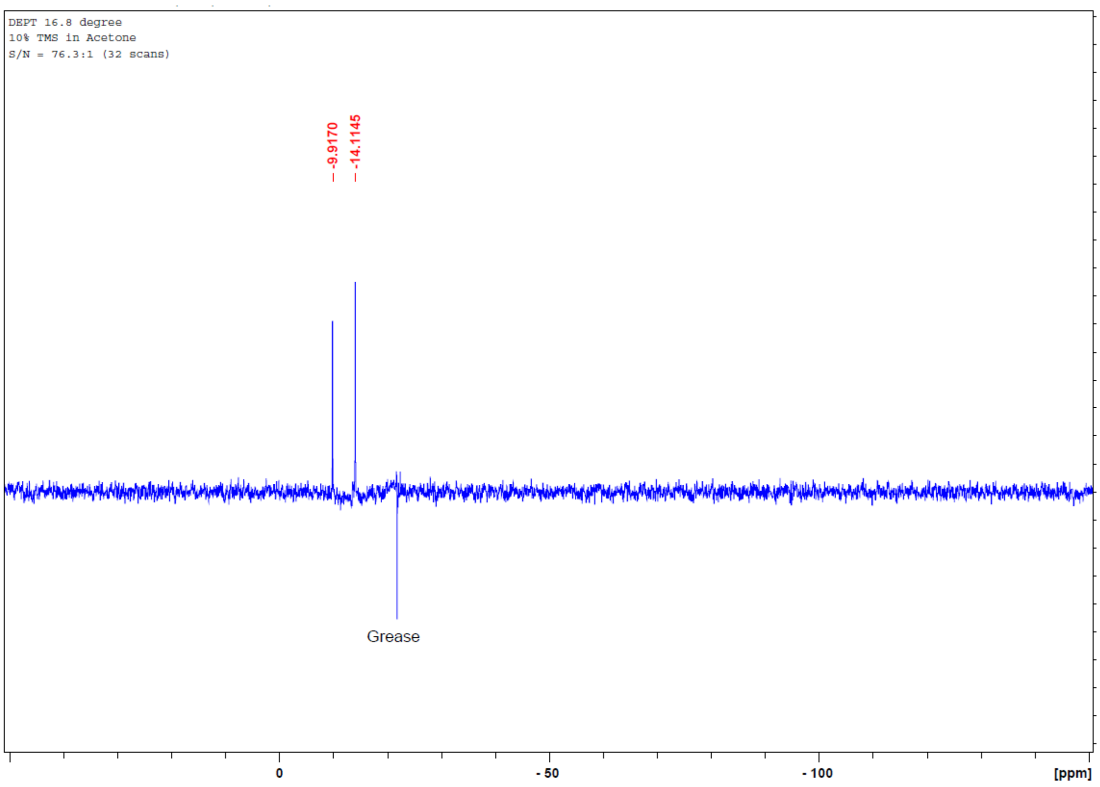

Supplementary Figure 46. ^{29}Si NMR spectrum of **8a** in $CDCl_3$.

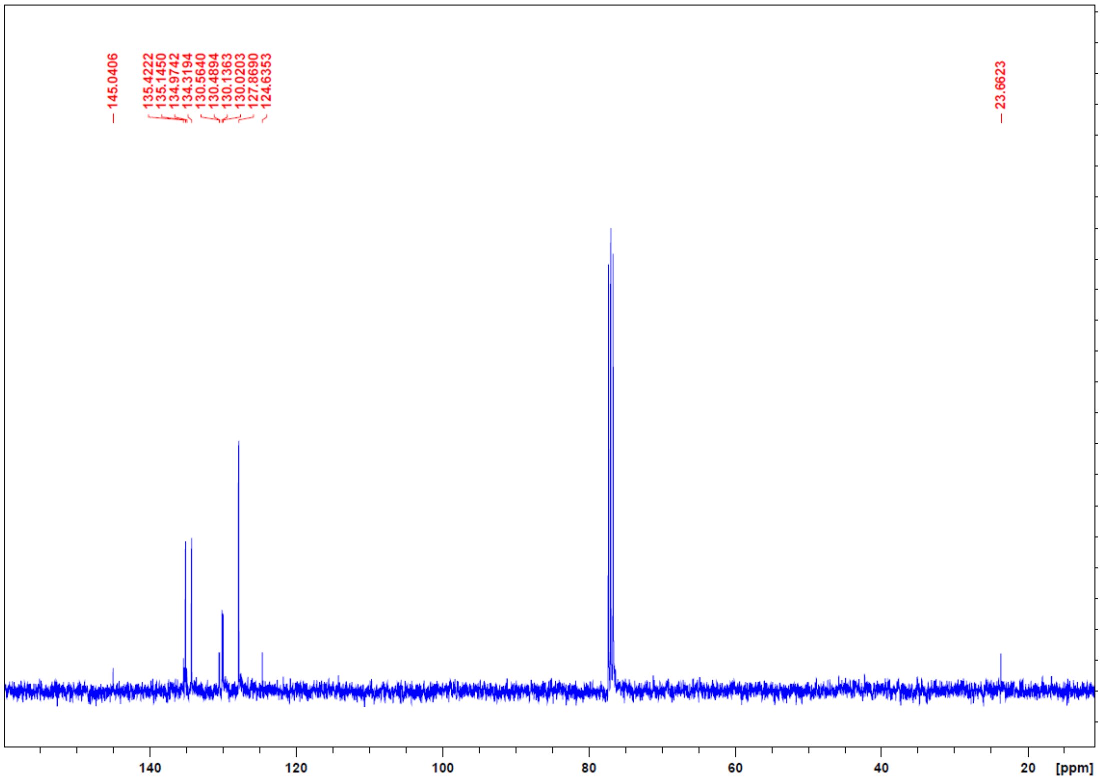

Supplementary Figure 47. ^{13}C NMR spectrum of **8a** in $CDCl_3$.

**Analysis Info**		Acquisition Date	1/11/2019 6:23:53 PM
Analysis Name	Y:\Mansa 2019\Samples run\November\191101\runlow2\VK19-18_RB4_01_12518.d		
Method	low_hplc.m	Operator	Admin
Sample Name	VK19-18	Instrument	micrOTOF-Q
Comment	Sample dissolved to 1 mg/mL in MeCN<br>Sampe diluted 1.5 µL in 0.5 mL MeCN		

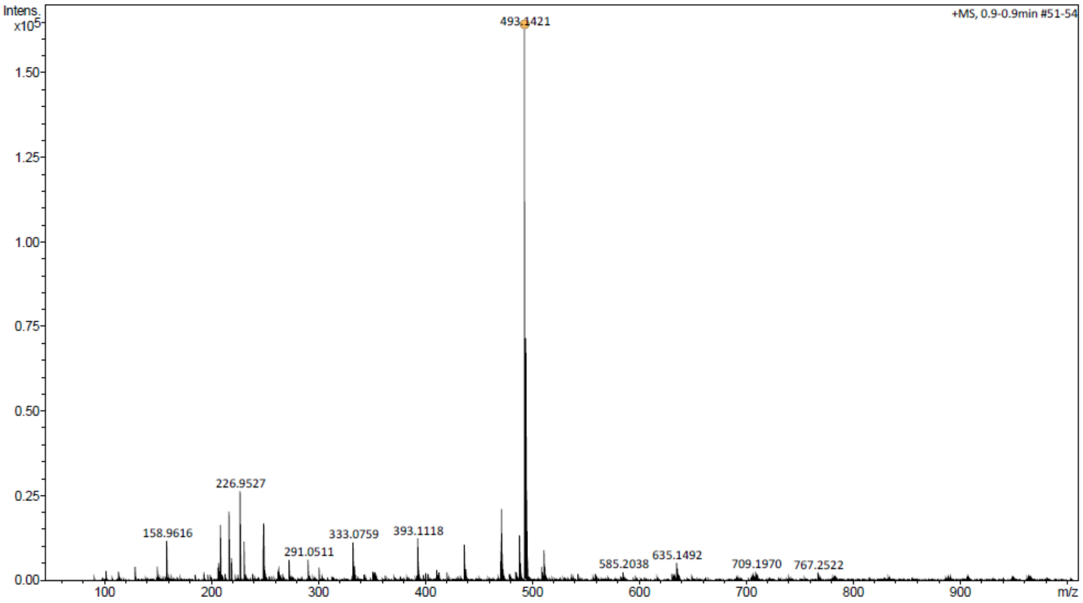

Supplementary Figure 48. ESI-MS of **8a**.

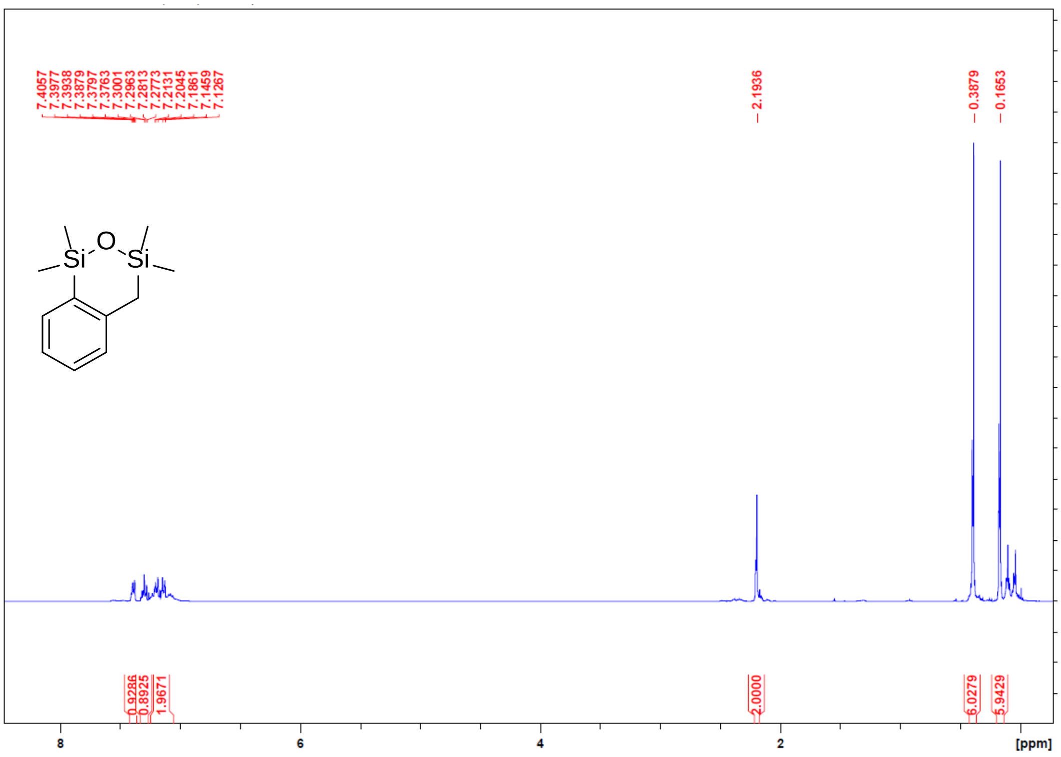

Supplementary Figure 49. ^{1}H NMR spectrum of **8b** in $CDCl_3$.

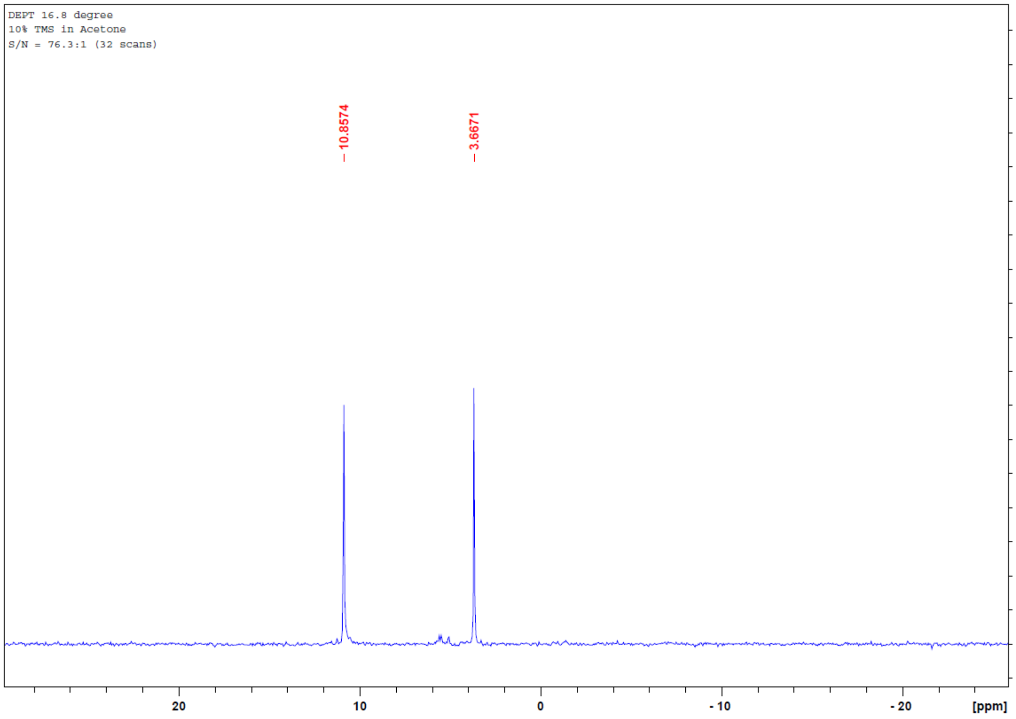

Supplementary Figure 50. ^{29}Si NMR spectrum of **8b** in $CDCl_3$.

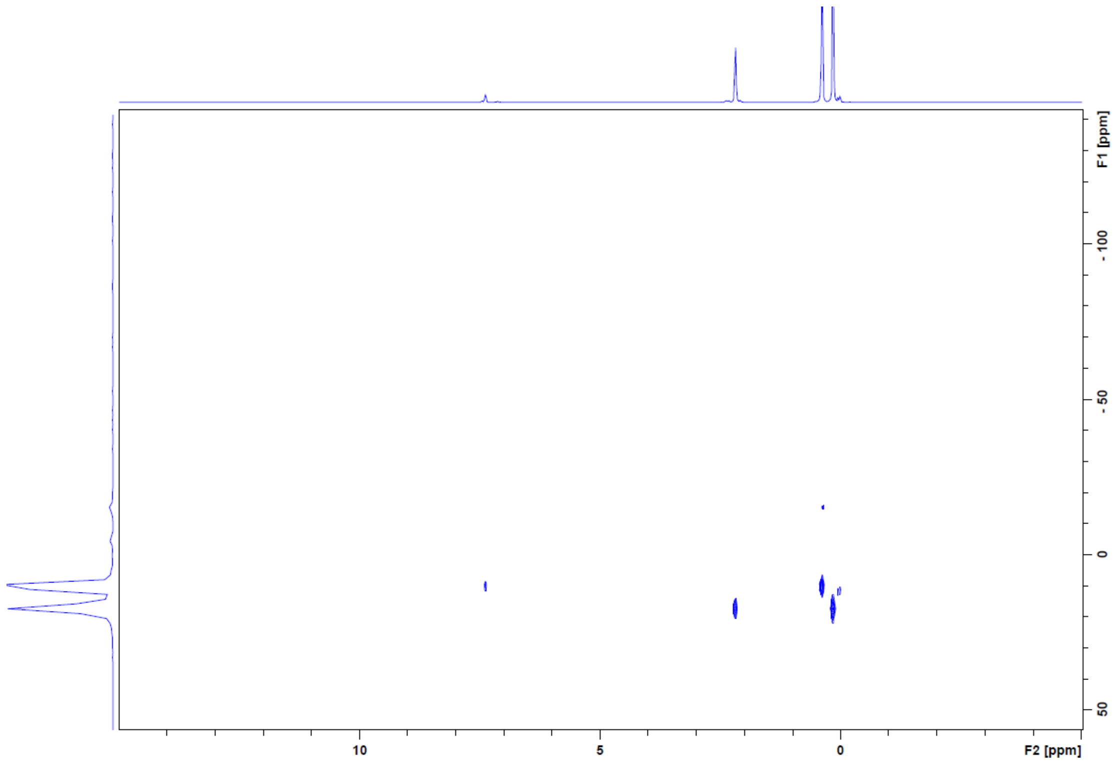

Supplementary Figure 51. $^{29}Si\{^{1}H\}$-HMBC NMR spectrum of **8b** in $CDCl_3$.

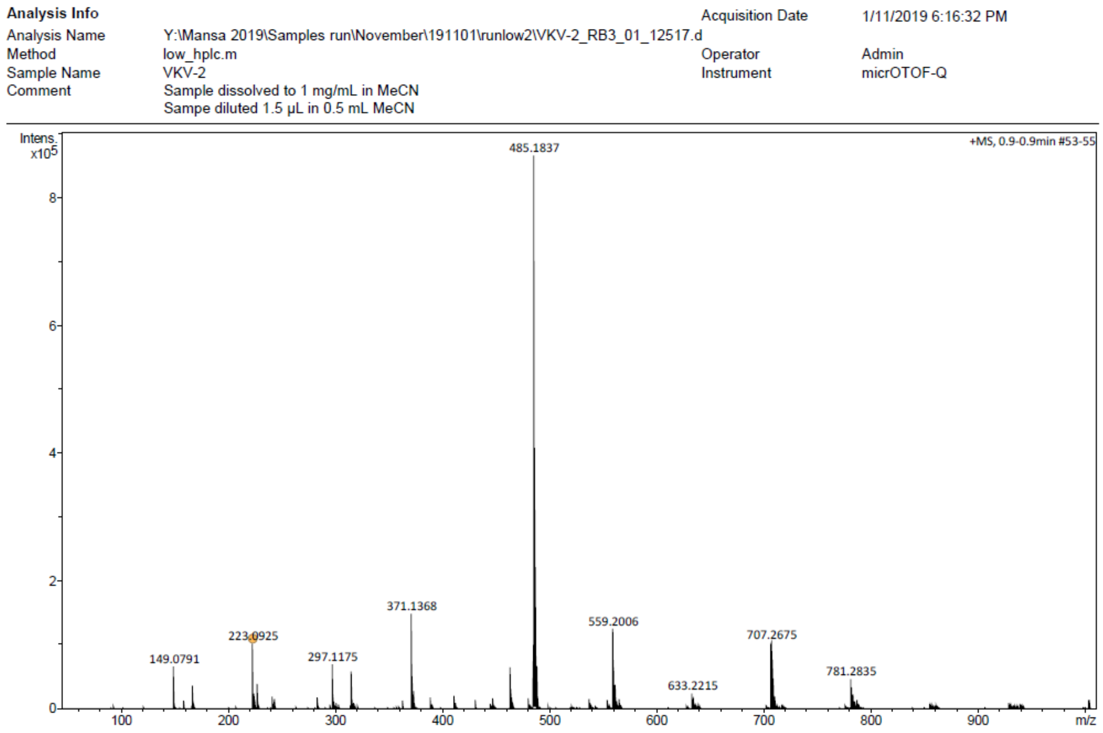

Supplementary Figure 52. ESI-MS of **8b**.

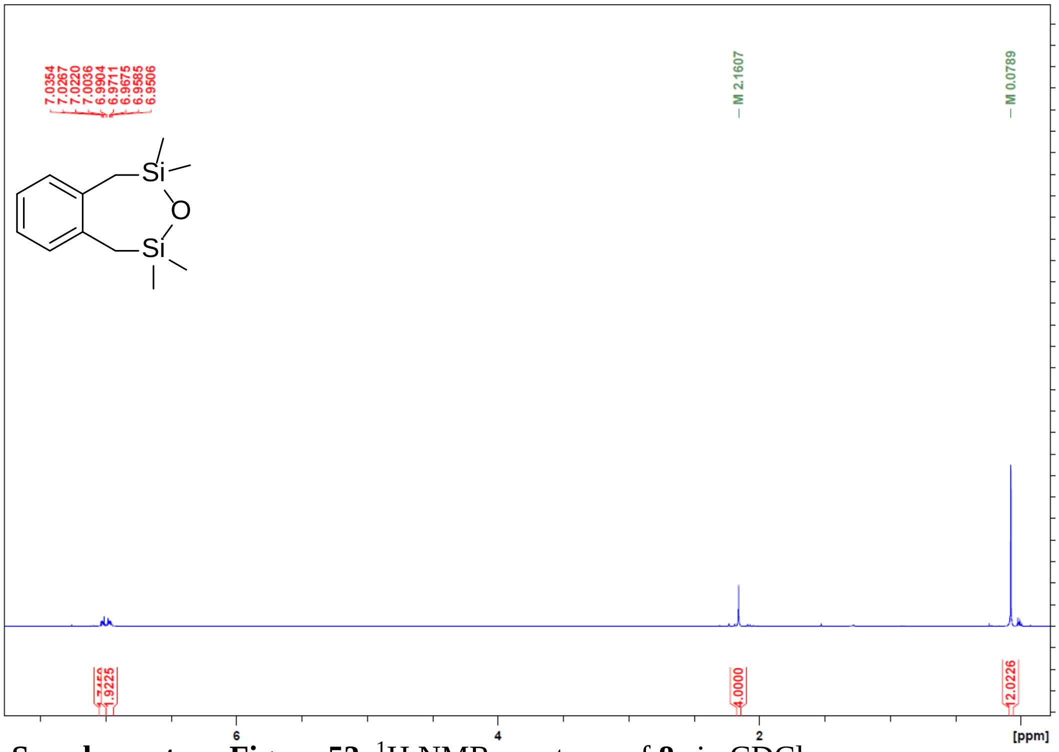

Supplementary Figure 53. ^{1}H NMR spectrum of **8c** in $CDCl_3$.

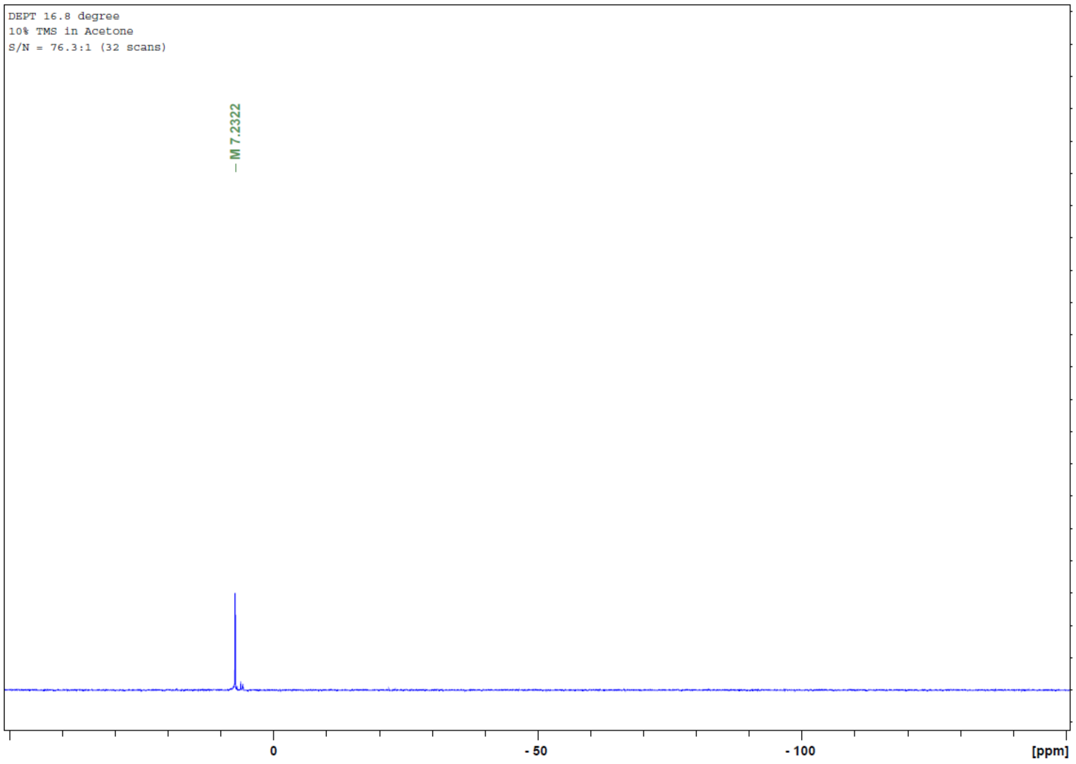

Supplementary Figure 54. ^{29}Si NMR spectrum of **8c** in $CDCl_3$.

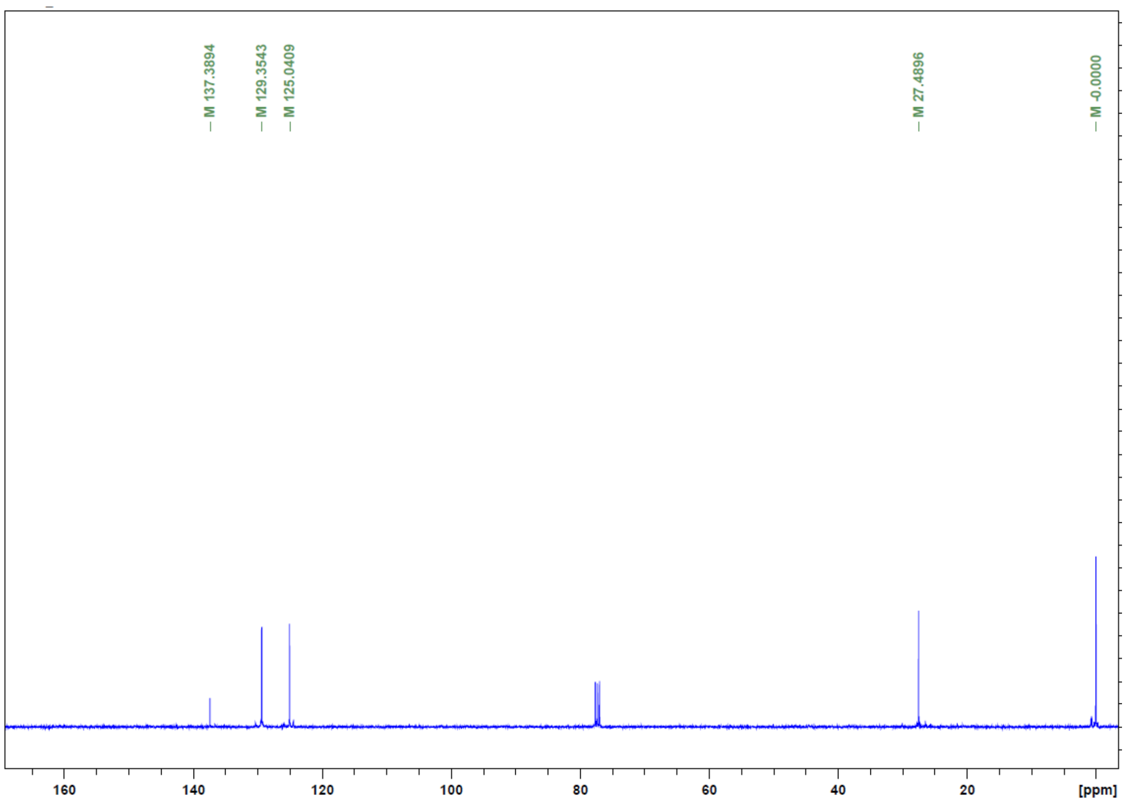

Supplementary Figure 55. ^{13}C NMR spectrum of **8c** in $CDCl_3$.

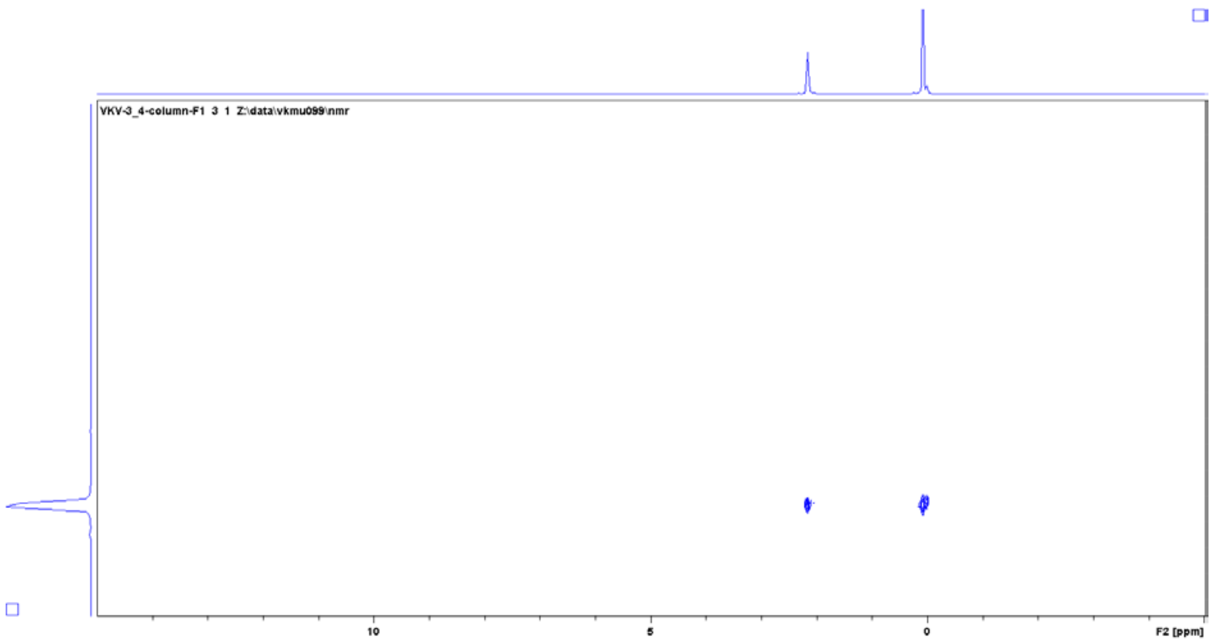

Supplementary Figure 56. $^{29}Si\{^{1}H\}$-HMBC NMR spectrum of **8c** in $CDCl_3$.

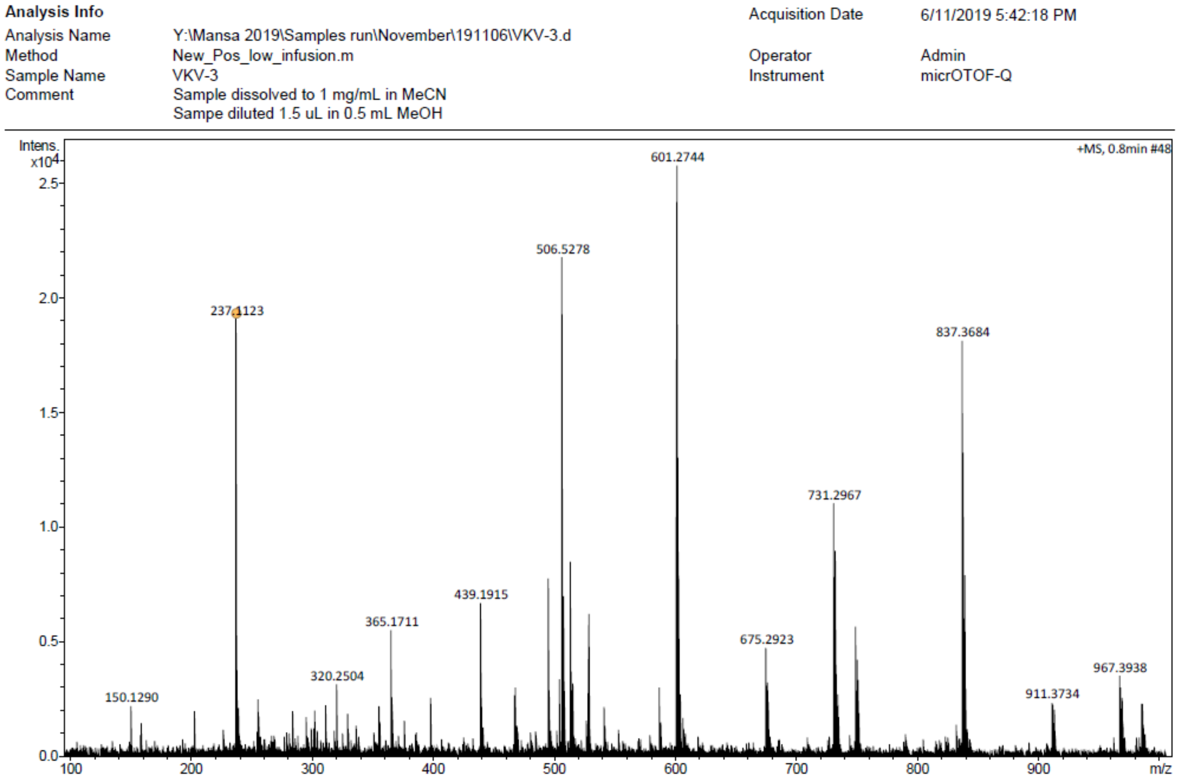

Supplementary Figure 57. ESI-MS of **8c**.

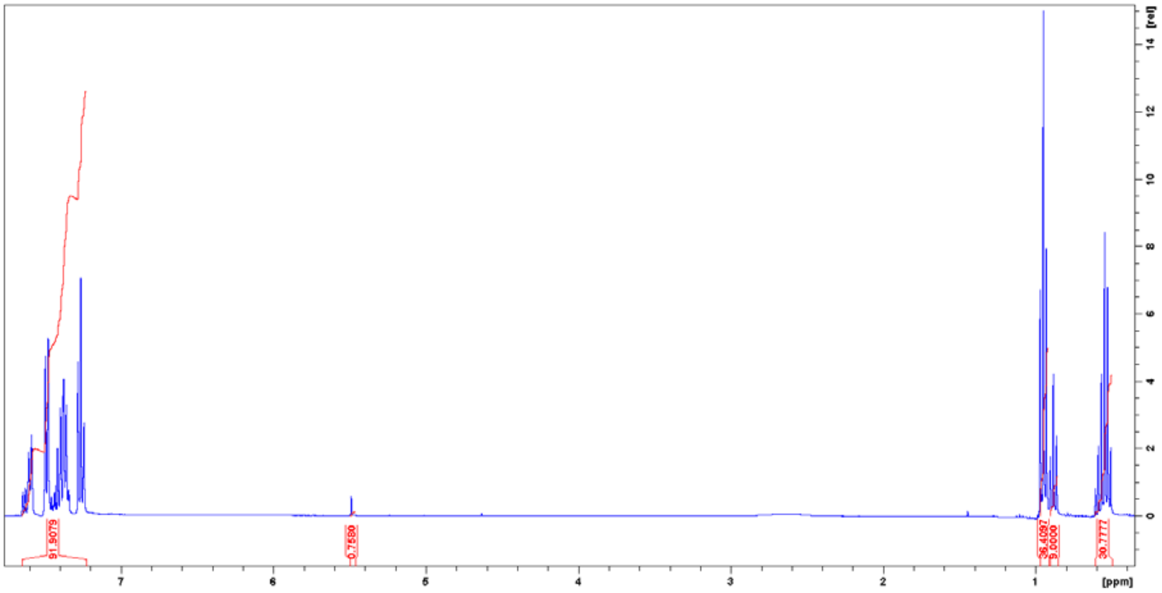

Supplementary Figure 58. ^{1}H NMR spectrum of 1:1 reaction of Ph_3SiH (**1c**) and Et_3SiH (**1a**) using 5.0 mol% $(C_6F_5)_3B(OH_2)$ and 1.0 eq H_2O in $CDCl_3$

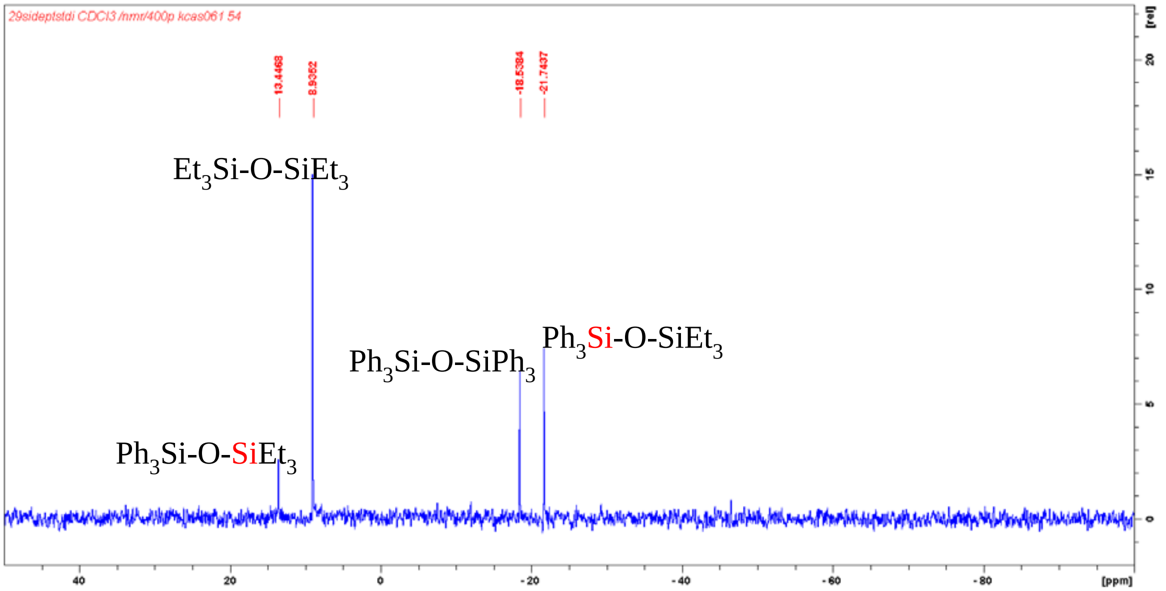

Supplementary Figure 59. $^{29}Si\{^1H\}$ NMR spectrum of 1:1 reaction of Ph_3SiH (**1c**) and Et_3SiH (**1a**) using 5.0 mol% $(C_6F_5)_3B(OH_2)$ and 1.0 eq H_2O in $CDCl_3$

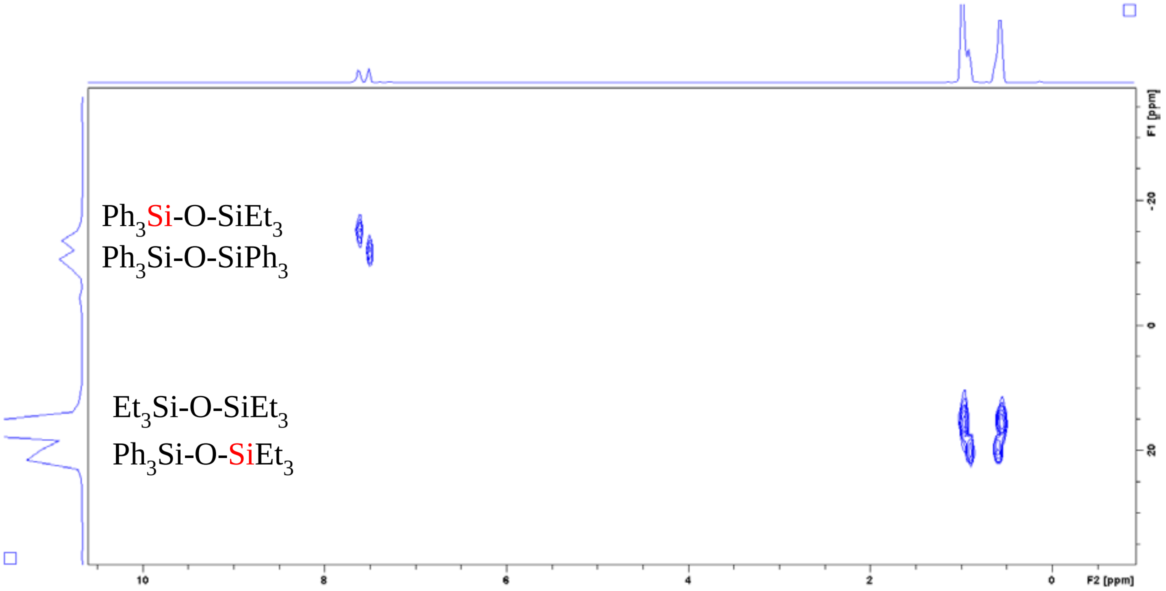

Supplementary Figure 60. $^{29}Si\{^1H\}$-HMBC NMR spectrum of 1:1 reaction of Ph_3SiH (**1c**) and Et_3SiH (**1a**) using 5.0 mol% $(C_6F_5)_3B(OH_2)$ and 1.0 eq H_2O in $CDCl_3$

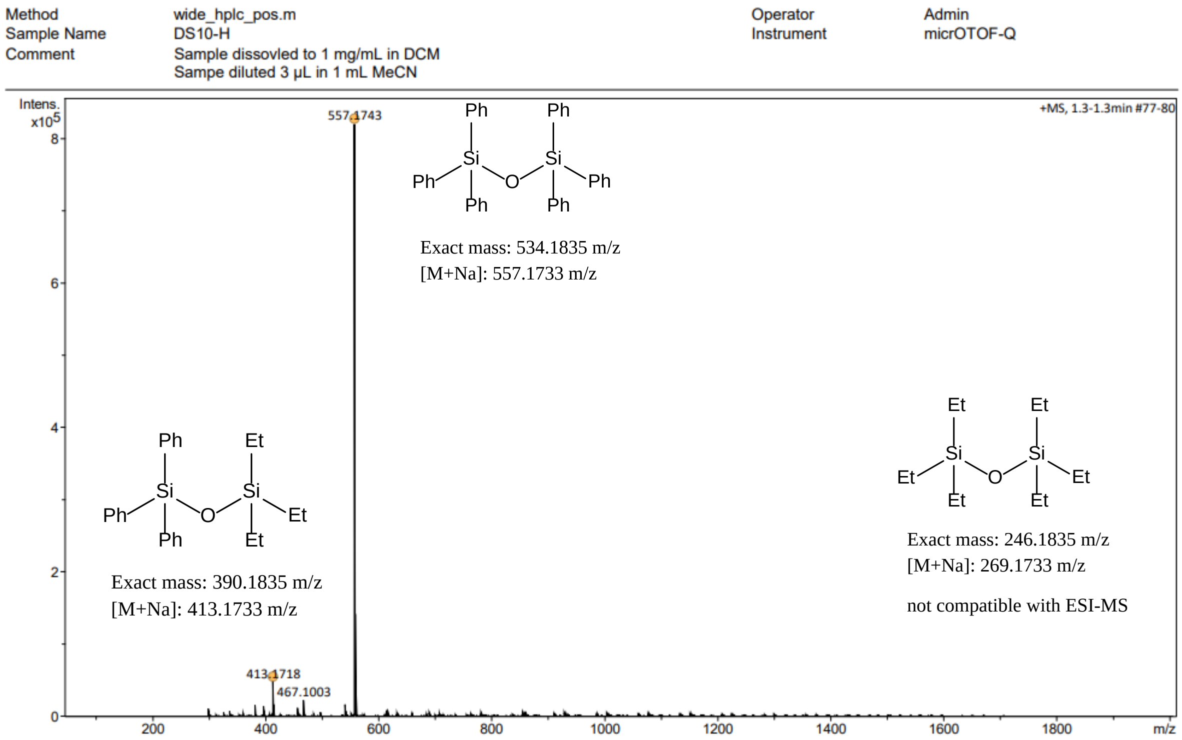

Supplementary Figure 61. ESI-MS of 1:1 reaction of Ph_3SiH (**1c**) and Et_3SiH (**1a**) using 5.0 mol% $(C_6F_5)_3B(OH_2)$ and 1.0 eq H_2O

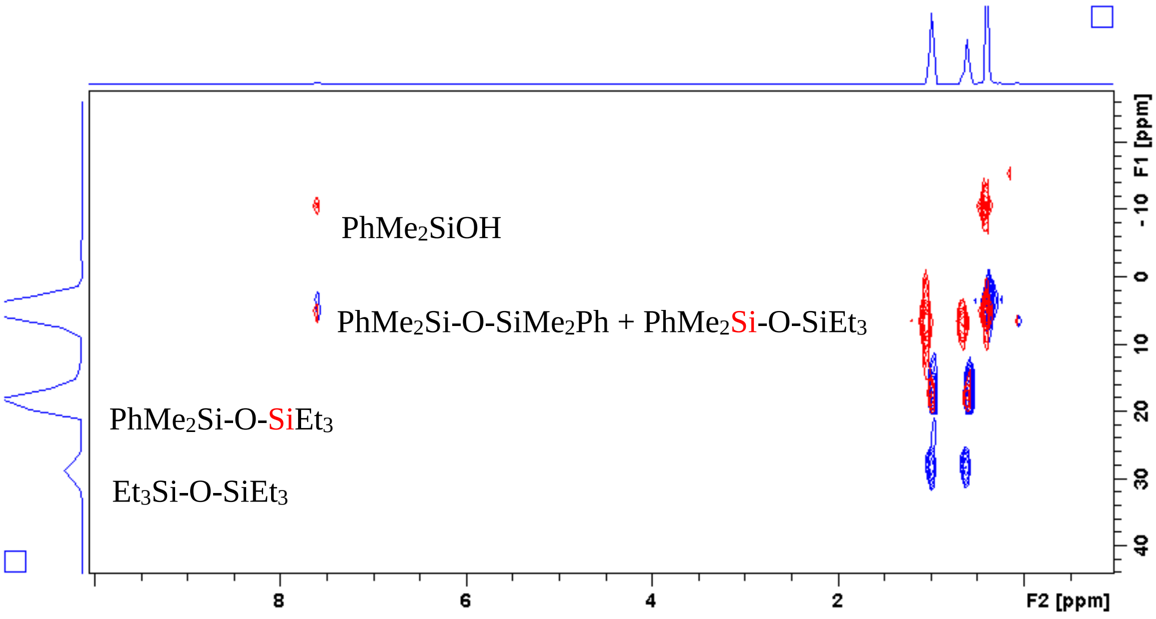

Supplementary Figure 62. $^{29}Si\{^1H\}$ HMBC NMR spectrum of 1:1 reaction of $PhMe_2SiH$ (**1b**) and Et_3SiOH (**2a**) (blue) and 1:1 reaction of $PhMe_2SiH$ (**1b**) and Et_3SiH (**1a**) (red) using 5.0 mol% $(C_6F_5)_3B(OH_2)$ after 1 h in $CDCl_3$

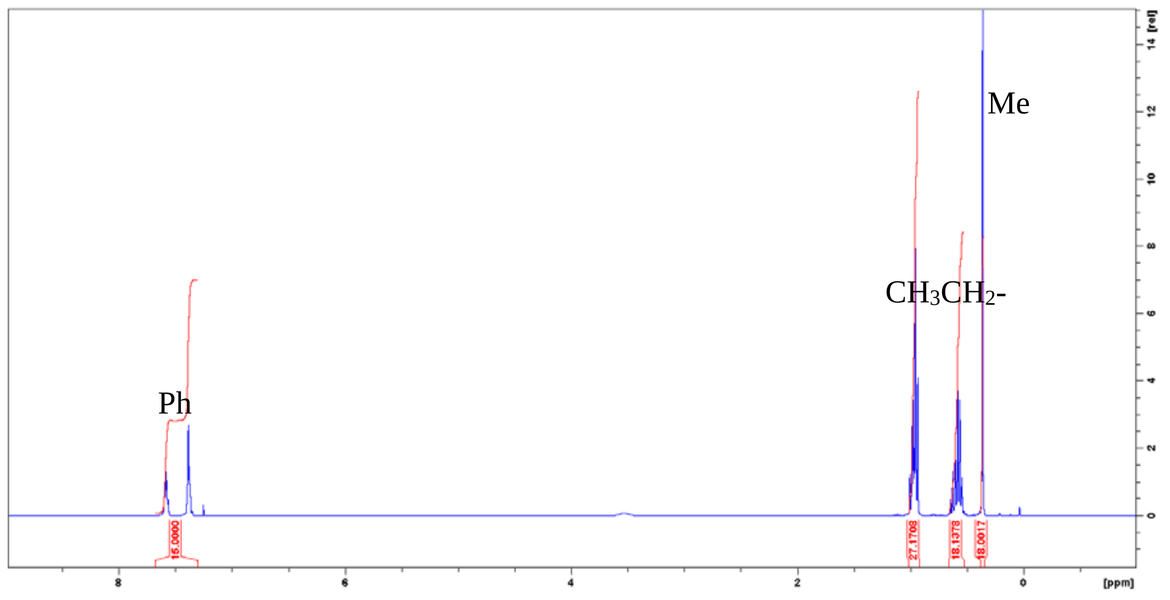

Supplementary Figure 63. ^{1}H NMR spectrum of 1:1 reaction of $PhMe_2SiH$ (**1b**) and Et_3SiOH (**2a**) using 5.0 mol% $(C_6F_5)_3B(OH_2)$ in $CDCl_3$

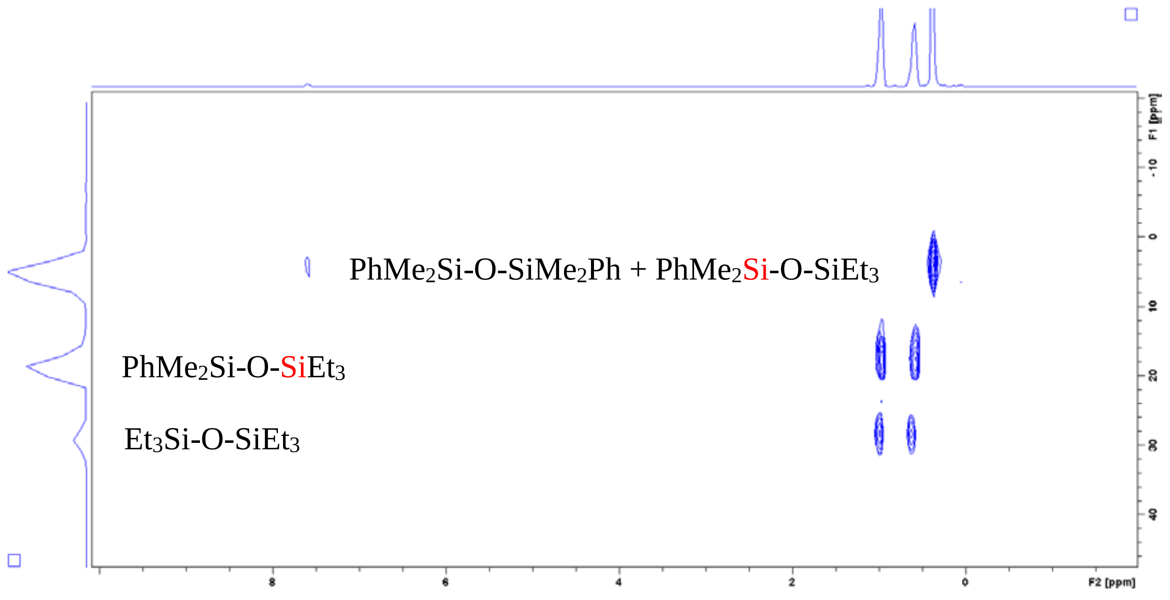

Supplementary Figure 64. ^{29}Si{^{1}H} HMBC NMR spectrum of 1:1 reaction of $PhMe_2SiH$ (**1b**) and Et_3SiOH (**2a**) using 5.0 mol% $(C_6F_5)_3B(OH_2)$ in $CDCl_3$

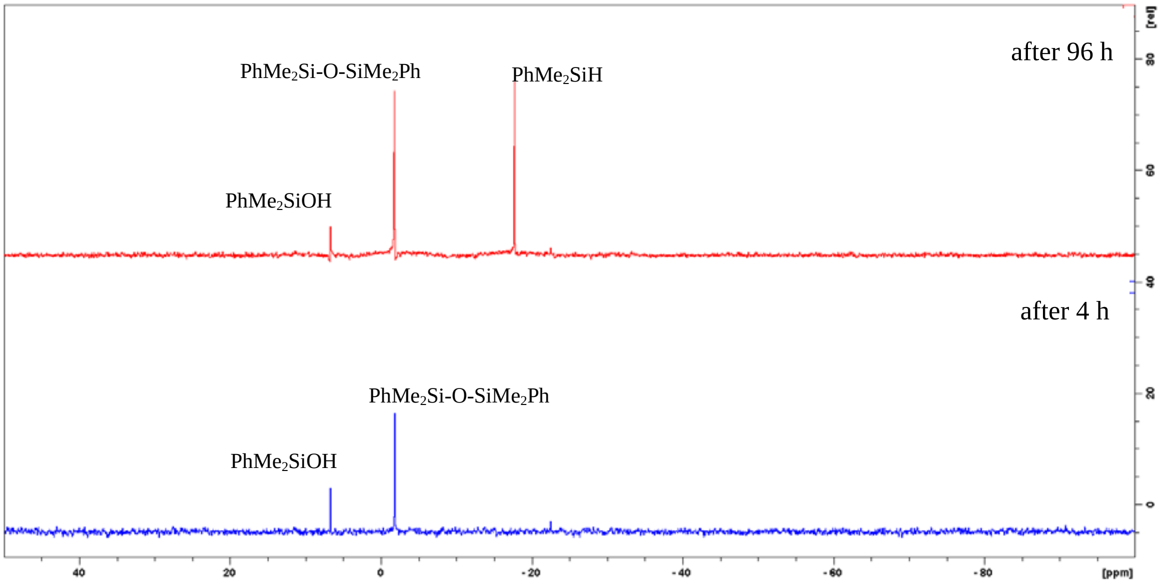

Supplementary Figure 65. ^{29}Si{^{1}H} NMR spectra of $PhMe_2SiH$ (**1b**) and 0.1 mol% $(C_6F_5)_3B(OH_2)$ in DMSO-d$_6$ (NMR tube reaction)

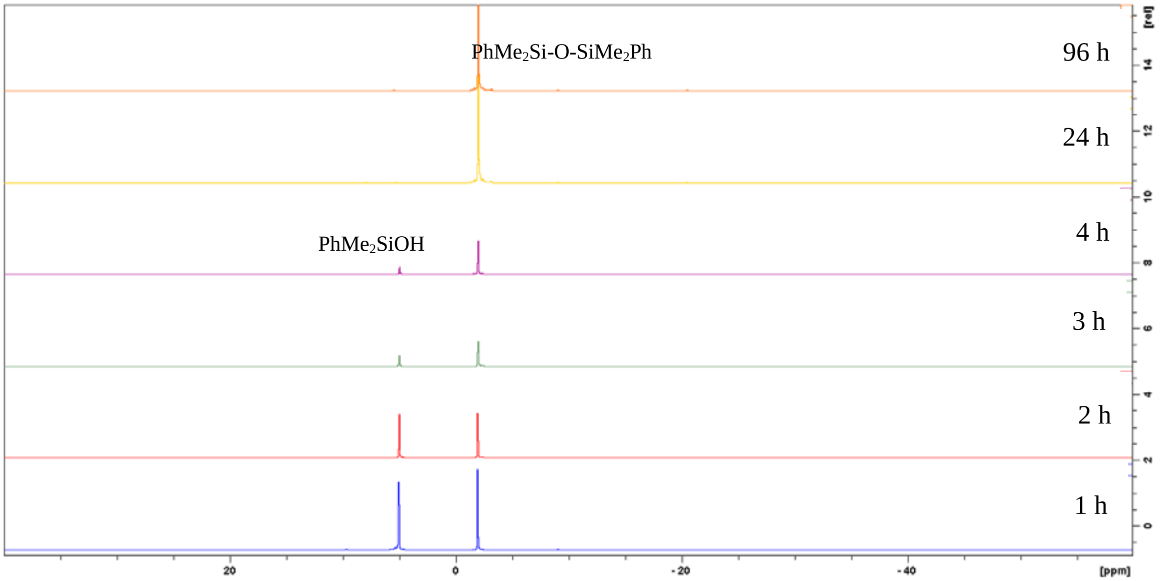

Supplementary Figure 66. ^{29}Si{^{1}H} NMR spectra of $PhMe_2SiOH$ (**2b**) and 0.1 mol% $(C_6F_5)_3B(OH_2)$ over time in DMSO-d$_6$ (NMR tube reaction)

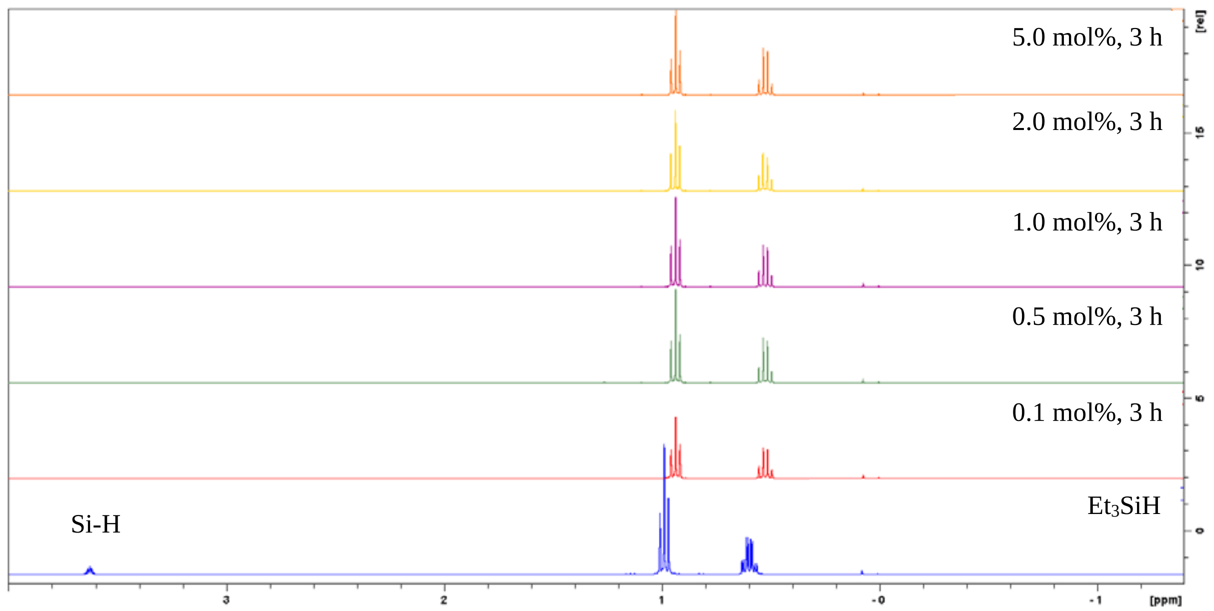

Supplementary Figure 67. ^{1}H NMR spectra in $CDCl_3$ of $Et_3SiOSiEt_3$ (**3a**) synthesized from Et_3SiH (**1a**) using different catalyst loadings for 3 h

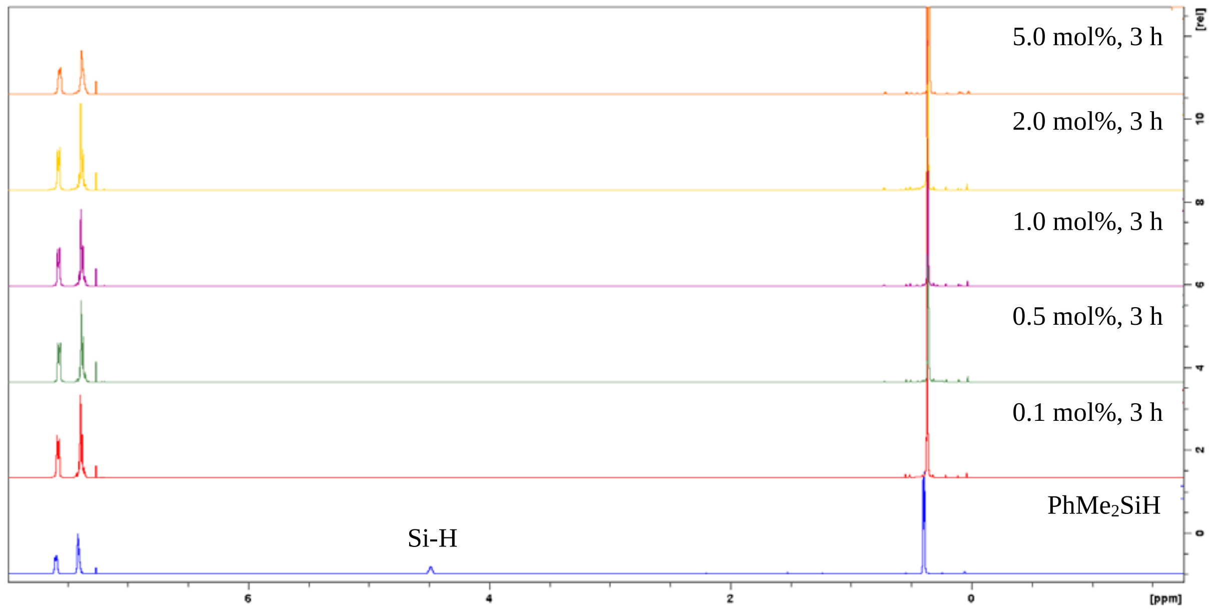

Supplementary Figure 68. ^{1}H NMR spectra in $CDCl_3$ of $PhMe_2SiOSiMe_2Ph$ (**3b**) synthesized from $PhMe_2SiH$ (**1b**) using different catalyst loadings for 1 h

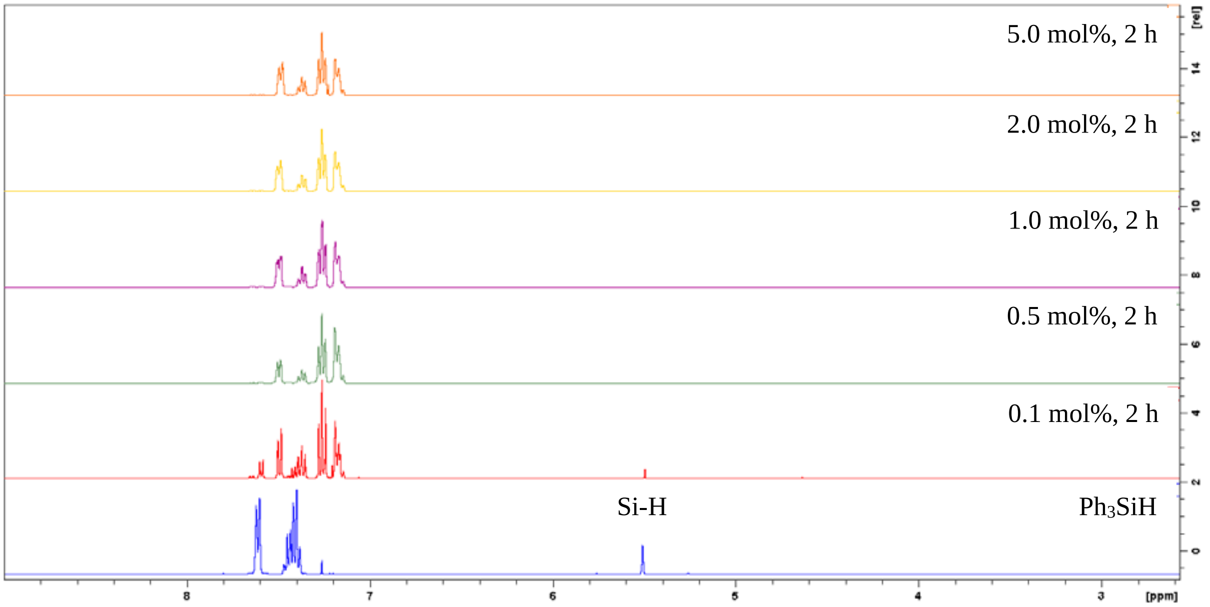

Supplementary Figure 69. ^{1}H NMR spectra in $CDCl_3$ of $Ph_3SiOSiPh_3$ (**3c**) synthesized from Ph_3SiH (**1c**) using different catalyst loadings for 2 h

Supplementary Table 1. X-ray crystallographic data for compounds 7a and 8a.

**Compound**	**7a**	**8a**
CCDC deposition number	1987431	1987430
Chemical Formula	$C_{31}H_{28}Si_2$	$C_{31}H_{26}OSi_2$
M / g mol^{-1}	456.71	470.70
Temperature / K	109.92(17)	103(5)
Crystal system	monoclinic	monoclinic
Space group	$I2/a$	$P2_1$
a /Å	25.1499(5)	16.9084(4)
b /Å	8.09860(10)	9.4869(2)
c /Å	26.7004(5)	17.5229(4)
β /°	113.552(2)	118.070(3)
Volume / Å^3	4985.29(16)	2480.19(11)
Z	8	4
ρ_{calc} /g/cm^3	1.217	1.261
μ/mm^{-1}	1.403	1.459
F(000)	1936.0	992.0
Crystal size /mm^3	0.15 × 0.12 × 0.1	0.22 × 0.18 × 0.1
Radiation	CuKα (λ=1.54184)	CuKα (λ=1.54184)
2Θ range /°	11.51 to 135.462	11.088 to 136.492
Index ranges	$-30\leq h\leq 30$, $-9\leq k\leq 9$, $-30\leq l\leq 32$	$-20\leq h\leq 17$, $-11\leq k\leq 11$, $-16\leq l\leq 21$
Reflections collected	37892	9005
Independent reflections (R_{int}) Restraints/parameters	4518(0.0436)/0/306	9005 (0.0399*)/1/614
Goodness-of-Fit on F^2	1.051	1.125
Final R indexes [I>=2σ(I)]	R_1= 0.0372, wR_2= 0.0948	R_1= 0.0489, wR_2 = 0.1385
Final R indexes [all data]	R_1= 0.0393, wR_2= 0.0961	R_1= 0.0505, wR_2= 0.1393
Largest diff. peak/hole/ e Å^{-3}	0.41/-0.37	0.84/-0.41
Flack parameter / twin component	-	0.002(10); 0.745(4)

*original twinned data set

Supplementary Data: catalyst recycling studies

Supplementary Table 2. Isolated yield of $Et_3SiOSiEt_3$ (**3a**) at different catalyst loadings of $(C_6F_5)_3B(OH_2)$ after 3 h reaction time

Catalyst Loading, mol%	**3a**, % yield					TON (from 1st cycle)	TOF, h^{-1}
	1st cycle	2nd cycle	3rd cycle	4th cycle	5th cycle		
0.1	50	4	*	*	*	500	167
0.5	64	66	66	64	60	128	43
1.0	66	67	78	69	73	66	22
2.0	63	78	*	*	*	32	11
5.0	62	77	*	*	*	12	4

***** not performed

Supplementary Table 3. Isolated yield of $PhMe_2SiOSiMe_2Ph$ (**3b**) at different catalyst loadings of $(C_6F_5)_3B(OH_2)$ after 1 h reaction time

Catalyst Loading, mol%	**3b**, % yield	TON	TOF, h^{-1}
0.1	90	900	900
0.5	94	188	188
1.0	94[a)]	94	94
2.0	92	46	46
5.0	94	19	19

a) average % yield of 5 cycles

Supplementary Table 4. Isolated yield of $Ph_3SiOSiPh_3$ (**3c**) at different catalyst loadings of $(C_6F_5)_3B(OH_2)$ after 2 h reaction time

Catalyst Loading, mol%	**3c**, % yield	TON	TOF, h^{-1}
0.1	97	970	485
0.5	97	194	97
1.0	98[a)]	98	46
2.0	98	46	23
5.0	97	19	10

a) average % yield of 5 cycles
